# Origin of exon skipping-rich transcriptomes in animals driven by evolution of gene architecture

**DOI:** 10.1186/s13059-018-1499-9

**Published:** 2018-09-17

**Authors:** Xavier Grau-Bové, Iñaki Ruiz-Trillo, Manuel Irimia

**Affiliations:** 1 Institut de Biologia Evolutiva (CSIC-Universitat Pompeu Fabra), Passeig Marítim de la Barceloneta 37-49, 08003 Barcelona, Catalonia Spain; 2 Departament de Genètica, Microbiologia i Estadística, Universitat de Barelona, Avinguda Diagonal 643, 08028 Barcelona, Catalonia Spain; 3 ICREA, Passeig Lluís Companys 23, 08010 Barcelona, Catalonia Spain; 4 Centre de Regulació Genòmica, Barcelona Institute of Science and Technology, Dr. Aiguader 88, 08003 Barcelona, Catalonia Spain; 5 Universitat Pompeu Fabra (UPF), Plaça de la Mercè 10-12, 08002 Barcelona, Catalonia Spain

**Keywords:** Alternative splicing, Exon skipping, Intron retention, Ancestral reconstruction, Gene architecture, Evolution of transcriptome regulation

## Abstract

**Background:**

Alternative splicing, particularly through intron retention and exon skipping, is a major layer of pre-translational regulation in eukaryotes. While intron retention is believed to be the most prevalent mode across non-animal eukaryotes, animals have unusually high rates of exon skipping. However, when and how this high prevalence of exon skipping evolved is unknown. Since exon skipping can greatly expand proteomes, answering these questions sheds light on the evolution of higher organismal complexity in metazoans.

**Results:**

We used RNA-seq data to quantify exon skipping and intron retention frequencies across 65 eukaryotic species, with particular focus on early branching animals and unicellular holozoans. We found that only bilaterians have significantly increased their exon skipping frequencies compared to all other eukaryotic groups. Unlike in other eukaryotes, however, exon skipping in nearly all animals, including non-bilaterians, is strongly enriched for frame-preserving sequences, suggesting that exon skipping involvement in proteome expansion predated the increase in frequency. We also identified architectural features consistently associated with higher exon skipping rates within all studied eukaryotic genomes. Remarkably, these architectures became more prevalent during animal evolution, indicating co-evolution between genome architectures and exon skipping frequencies.

**Conclusion:**

We suggest that the increase of exon skipping rates in animals followed a two-step process. First, exon skipping in early animals became enriched for frame-preserving events. Second, bilaterian ancestors dramatically increased their exon skipping frequencies, likely driven by the interplay between a shift in their genome architectures towards more exon definition and recruitment of frame-preserving exon skipping events to functionally diversify their cell-specific proteomes.

**Electronic supplementary material:**

The online version of this article (10.1186/s13059-018-1499-9) contains supplementary material, which is available to authorized users.

## Background

Alternative splicing (AS) is a pre-translational process that allows the creation of multiple messenger RNA (mRNA) transcripts from a single gene by differentially selecting splice sites in multi-exonic sequences [1]. This phenomenon can contribute to the regulation of gene expression [2–7], or the creation of multiple protein isoforms per gene, increasing the proteomic repertoire of eukaryotic genomes [8, 9] and potentially leading to key evolutionary innovations [10–12].

The main forms of AS among eukaryotes are the exclusion of specific exons and the retention of introns in the final transcripts [1, 13], referred to as exon skipping (ES) and intron retention (IR), respectively. These sources of transcript variation are widespread in eukaryotes, but initial studies revealed that the prevalence of each AS mode varied across lineages: animals show higher rates of ES than other eukaryotes, whereas IR is frequent across all eukaryotic groups, including animals, fungi, plants, and various protist lineages [14–17]. This contrast led to the proposition that ES-rich AS profiles were a major contributor to the increased phenotypic complexity of animals, since richer proteomes can provide an expanded tool-kit needed to sustain multicellularity [8, 18]. Consistently, and although the extent to which ES transcripts are translated and functional is still under debate [19, 20], many ES-derived isoforms have been found to be physiologically relevant in animals (reviewed in [8, 21]), for example, by tuning protein–protein interaction networks [22–24]. In contrast, IR events have been linked to down-regulation of gene expression via the nonsense-mediated decay (NMD) pathway [4–6, 25], nuclear retention [7] or intron detention [3] in a wide variety of eukaryotes.

The evolutionary origin of AS can be tracked to the last eukaryotic common ancestor (LECA), which already had an intron-dense genome [26, 27] with heterogeneous splice sites [28–30] and all the essential splicing machinery (the spliceosome, a complex of small nuclear RNAs and dozens of assisting protein factors) [27, 31, 32]. These observations have allowed inferring that the earliest eukaryotes already exhibited splicing-rich transcriptomes yielding multiple mRNA variants per gene, mostly by IR [18, 33, 34]. However, it remains unclear when and how animal transcriptomes shifted towards higher frequencies of ES and recruited this mode of AS as a mechanism to expand their proteomes. First, the sampling of early-branching species—poriferans, cnidarians, placozoans, and ctenophores—is scarce. Second, no comprehensive comparative study using high-throughput RNA sequencing (RNA-seq) data has been performed to date. Third, relative increases in ES frequency have also been identified in other phylogenetically scattered eukaryotes—e.g. in plants, *Volvox carteri*, or the chlorarachniophyte *Bigelowiella natans* [10, 35–38].

**Fig. 1:**
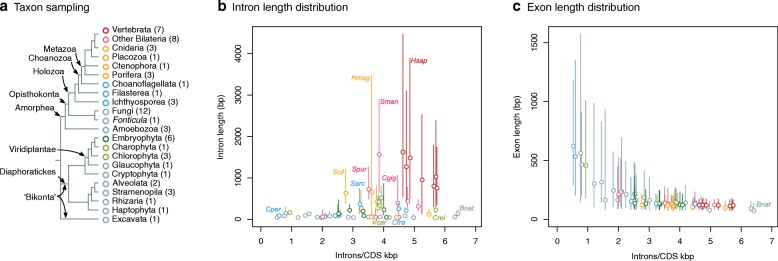
Intron/exon structure across eukaryotes. **a** Summary of the taxon sampling used in this study and the phylogenetic relationships between species (Additional file 1: Figure S1). **b, c** Comparison of intron density (introns/CDS kbp) and intron (**b**) and exon (**c**) length distribution (in bp) in 65 eukaryotic genomes. *Dots* represent median intron/exon lengths and *vertical lines* delimit the first and third quartiles (Additional file 1: Figure S2). Color-coded according to taxonomy

Here, we address these questions by analyzing RNA-seq-derived AS profiles for 65 eukaryotic species. Using this comprehensive dataset of joint transcriptomic and genomic data, we track the frequency of both main modes of AS (IR and ES) across all major eukaryotic lineages (Fig. 1a, Additional file 1: Figure S1), uncovering the phylogenetic patterns behind AS evolution. Specifically, we investigate the transition towards high ES frequencies in multicellular lineages (animals and plants) by comparing their AS profiles and genome architectures with their closest unicellular relatives. We find that the frequency of ES rose mainly in bilaterians, with only mild increases in non-bilaterians. However, we show that recruitment of ES for proteome expansion predated the bilaterian increase in ES and occurred early in metazoan evolution. Furthermore, we uncover a set of sequence and architectural features that influence the frequency of ES and IR in transcripts across eukaryotes, suggesting the existence of a soft pan-eukaryotic *cis-*regulatory code for AS determination. Using this code and reconstruction of ancestral intron–exon architectures we evaluated the step-wise increase of ES along animal evolution.

## Results

### ES frequency increased largely in bilaterian ancestors

We quantified the frequencies of ES and IR at the single exon and intron levels for 65 eukaryotic species (Fig. 1a), including a wide range of intron–exon architectures (Fig. 1b, c; Additional file 1: Figures S1 and S2). For this purpose, we compiled a large dataset of available RNA-seq data (Additional file 1: Figure S1) and performed de novo RNA-seq for three phylogenetically key species: the placozoan *Trichoplax adhaerens*, the holozoan *Sphaeroforma arctica*, and the intron-rich excavate *Naegleria gruberi*. For the analysis of ES events (Additional file 1: Figure S3), we compiled a dataset of exon triplets from 2.93 × 10^6^ internal exons from 5.08 × 10^5^ multi-exonic genes with transcriptomic support. Each internal exon was classified as ES-negative (0–10% exon skipping rate [*r*_*ES*_] and sufficient read coverage; 89.29% of the dataset), ES-positive (*r*_*ES*_ = 10–90% and sufficient read coverage; 0.74% of the data), or undetermined (all other cases; Additional file 1: Figure S4). For the analysis of IR (Additional file 1: Figure S3), an analogous dataset was built with 1.98 × 10^6^ introns from 3.88 × 10^5^ multi-exonic genes, which were classified as IR-negative (0–10% inclusion rate [*r*_*IR*_] and sufficient read coverage; 71.09% of the data), IR-positive (*r*_*IR*_ = 10–90%, and sufficient read coverage; 5.27% of the data), or undetermined (Additional file 1: Figure S4).

**Fig. 2:**
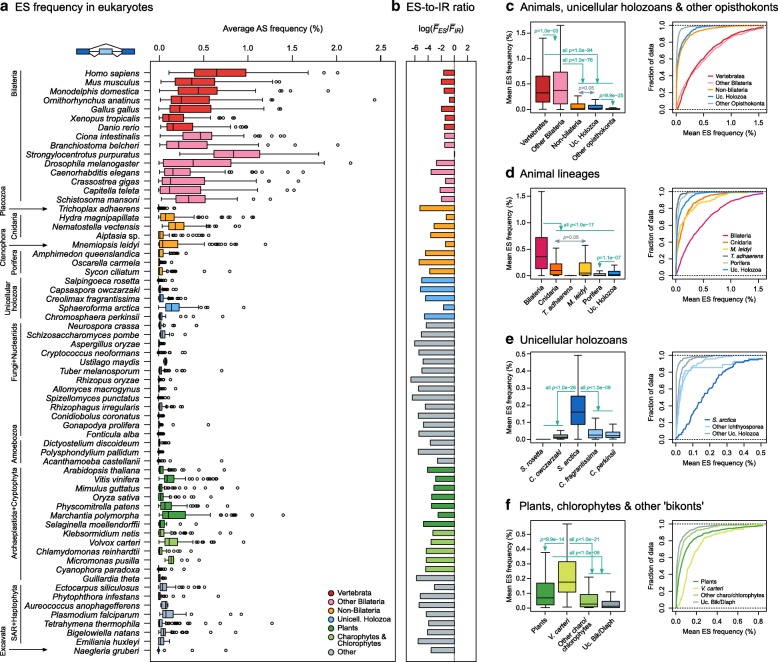
Frequency of AS in eukaryotes. **a** Distribution of ES frequencies across 65 eukaryotic transcriptomes. Each box plot represents the distribution of ES frequencies (*F*_*ES*,*sp*_) in a given species (as percentage), drawn from mapping up to 10,000 RNA-seq reads to junctions from 100 exon triplets, in up to 100 independent subsets (see “Methods” and Additional file 1: Figure S3). **b** Log-ratio of mean *F*_*ES*,*sp*_ (for ES) over mean *F*_*IR*,*sp*_ (for IR) in each species. *F*_*IR*,*sp*_ distributions in Additional file 1: Figure S6. **c–f** Comparisons of ES frequencies among phylogenetically related eukaryotic groups with a focus on animals, holozoans, and plants. Group-level ES distributions obtained by pooling *F*_*ES*,*sp*_ of multiple species. For each set of comparisons, we report the pooled distributions’ box plots (*left*) and empirical cumulative distributions (*right*). Selected *p* values are reported, corresponding to pairwise one-sided Kolmogorov-Smirnov two-sample tests (full report in Additional file 1: Figure S9), and should be interpreted as follows: *green arrows* indicate that the group of origin has significantly higher *F*_*ES*,*sp*_ than others; *grey bidirectional arrows* indicate that no significant differences are observed in any direction at the given threshold

Next, we examined the frequency of each AS mode at the species level by averaging exon- and intron-specific ES and IR rates across 100 subsets of exons or introns with normalized RNA-seq coverage (*F*_*ES*,*sp*_ and *F*_*IR*,*sp*_, respectively; see “Methods”; Additional file 1: Figures S3 and S5). This analysis produced three major results. First, we found evidence of ES and IR in transcriptomes from all studied eukaryotic lineages—namely, animals, fungi, opisthokont protists, amoebozoans, Viridiplantae, the cryptophyte *Guillardia theta*, SAR, the haptophyte *Emiliania huxleyi*, and the excavate *Naegleria gruberi* (Fig. 2, Additional file 1: Figures S4 and S6). Second, IR frequencies exceeded ES in all but one species (Fig. 2b). This result is in line with previous reports highlighting the dominance of IR-based AS across eukaryotes, but challenges initial views of animal transcriptomes as being dominated by ES. A possible explanation for this disagreement is the association between high IR rates and low transcript expression levels (see below), which hinders the detection of retained introns (particularly in studies based on EST data [14, 15, 39]).

Third, we found a clear phylogenetic pattern behind ES frequencies (Fig. 2a): animals, particularly bilaterians, had the highest frequencies, followed by non-bilaterians, plants, and a handful of other scattered eukaryotes. The consistency of our quantifications at the species level was assessed with replicate transcriptomic datasets for six selected ES-rich taxa obtained from independent studies (Additional file 1: Figure S7). Species-level *F*_*ES*,*sp*_ values were highly consistent between pairs of multi-organ transcriptomic datasets of adult human, frog, and tale cress and a comparison of developmental time series with various growth conditions for fruit fly (*p* > 0.01, Wilcoxon rank-sum test). Mild significant differences were observed between two multi-organ sets of mouse (*p* = 0.0037, Wilcoxon rank-sum test) and a comparison of developmental series of the sea anemone *Nematostella vectensis* (*p* = 1.67e − 08, Wilcoxon rank-sum test), but in both cases the distributions were within their respective taxonomic ranges. Similarly, although pooled samples generally had higher ES levels than the individual tissues, the latter also fell within the taxonomic range (Additional file 1: Figure S8). This indicates that our approach for ES quantification yields robust results independently of the experimental approaches used in different RNA-seq experiments.

To further investigate when the shift towards ES-rich transcriptomes occurred in animal evolution, we next compared the aggregated ES frequencies of vertebrates, non-vertebrate bilaterians, non-bilaterians (cnidarians, poriferans, the ctenophore *Mnemiopsis leidyi*, and the placozoan *Trichoplax adhaerens*), and their closest unicellular relatives in the Holozoa (choanoflagellates, *Capsaspora owczarzaki*, and ichthyosporeans) (Fig. 2c–f; complete report of statistical comparisons in Additional file 1: Figure S9).

Taken together, bilaterian animals have significantly higher *F*_*ES*,*sp*_ than non-bilaterians, unicellular holozoans, and other opisthokonts (Fig. 2c; *p* < 1.0e − 76 in all comparisons, one-sided Kolmogorov-Smirnov test [oKS]). Furthermore, vertebrates exhibited an enrichment compared to other bilaterians (*p* = 1.0e − 03). The grouping of non-bilaterians did not show significantly higher *F*_*ES*,*sp*_ than unicellular holozoans (Fig. 2c), although different patterns were observed when comparing against individual groups (Fig. 2d): *F*_*ES*,*sp*_ values were higher in cnidarians and the ctenophore *M. leidyi* and lower in poriferans and *T. adhaerens*. On the other hand, the ichthyosporean *Sphaeroforma arctica* had the highest *F*_*ES*,*sp*_ among unicellular holozoans (Fig. 2e; *p* < 1e − 09, oKS), which indicates a clear lineage-specific departure from the low incidence of ES in other unicellular holozoans [40, 41]. Importantly, these patterns were robust to different levels of read depth downsampling across species (Additional file 1: Figure S10). Therefore, these data show that (i) bilaterians, and vertebrates in particular, have a consistently higher ES frequency than their close relatives and other eukaryotes, and (ii) some non-bilaterian animals and unicellular holozoans have experienced relative increases in ES frequency as well.

In parallel, multicellular land plants also exhibited higher ES rates than other ‘bikonts’ (Dipahoratickes and *N. gruberi*), including their colonial and unicellular relatives in Chlorophyta and Charophyta (Fig. 2f; all *p* < 1.0e − 06, oKS). However, the colonial chlorophyte *Volvox carteri* was a notable exception, with higher *F*_*ES*,*sp*_ than other algae (including its close unicellular relative *Chlamydomonas reinhardtii* [38]) and most land plants (*p* < 1e − 13 for all comparisons, oKS).

Finally, our analysis of *F*_*ES*,*sp*_ levels in the chlorarachniophyte *Bigelowiella natans* showed contradictory results. The analysis of two recent transcriptomic datasets [42, 43] showed significantly lower ES frequencies than those reported in the original genome paper [36] (Additional file 1: Figure S7; *p* < 1e − 16, Wilcoxon rank-sum test). The reasons behind these differences remain elusive. One possible explanation is that they were caused by differences in environmental conditions, such as abiotic stress, which have been shown to lead to increased levels of both spurious ES and IR in other species [44–46].

### Enrichment in frame-preserving ES is common to all animal groups

Next, we examined the potential global impact of AS events on each species’ proteome by assessing their effect on the coding capacity of the resulting transcripts. Following previous studies [47], we divided exons or introns between those that had lengths divisible by three (henceforth ‘3n’) and those that did not, under the assumption that 3n sequences would not normally disrupt the open reading frame (ORF) integrity when alternatively spliced, whereas non-3n sequence lengths would cause frame-shifts, usually resulting in unproductive transcripts. Indeed, maintenance of the ORF integrity is strongly associated with functional and conserved ES events in animals [8, 47, 48].

**Fig. 3:**
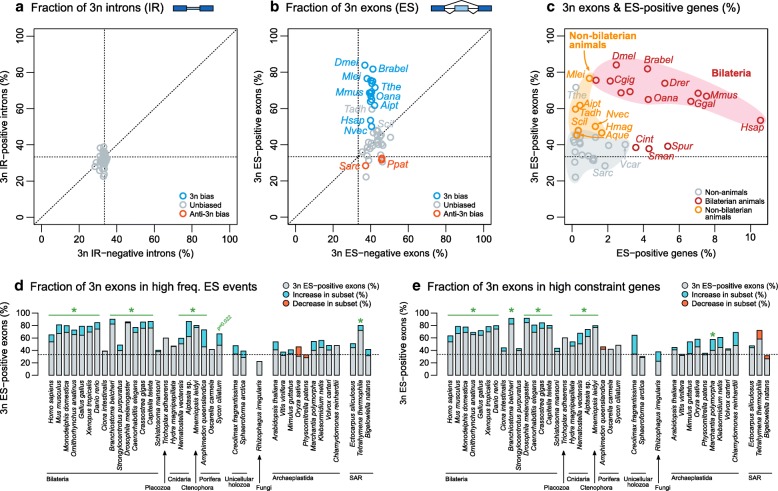
Fraction of 3n divisible exon and intron lengths. **a** Percentage of introns with 3n divisible lengths that were classified as IR-negative (*x-*axis) and IR-positive (*y*-axis) in each species with more than ten IR-positive introns. **b** Percentage of exons with 3n divisible lengths that were classified as ES-negative (*x*-axis) and ES-positive (*y*-axis) in each species with more than ten ES-positive exons. *Dots* are color-coded according to enrichments in either direction (3n bias in *blue*, anti-3n bias in *red*) as per Fisher’s exact test (*p* < 0.01; Additional file 1: Figure S9). **c** Percentage of ES-positive exons that are 3n (*y*-axis) with respect to the percentage of genes with ES-positive exons (*x*-axis), per species. Shading colors grouping the most representative species for certain eukaryotic groups (bilaterians, non-bilaterians, and other eukaryotes) are provided for reference. **d** Fraction of 3n exons in ES events (*r*_*ES*_ = 10–90%, *grey bar segment*) compared to a subset of high-frequency events (*r*_*ES*_ = 30–70%), highlighting increases (*blue*) or decreases (*red*) of the 3n fraction. In panels **d** and **e**, asterisks indicate significant 3n biases in ES-positive compared to ES-negative exons, as per Fisher’s exact test (*p* < 0.01; Additional file 1: Figure S9). In all panels, only species with more than ten exons in all categories are included. **e** Fraction of 3n exons in ES events (*grey bar segment*) compared to a subset of genes with above-median expression (cRPKM) and gene length (‘high constraint’), highlighting increases (*blue*) or decreases (*red*) of the 3n fraction

In the case of IR, virtually no significant biases towards or against 3n divisibility were observed (Fig. 3a and Additional file 1: Figure S11). In contrast, we found that alternatively spliced exons of most animals were significantly enriched in 3n divisible lengths (Fig. 3b, Additional file 1: Figure S12; *p* < 0.01, Fisher’s exact test). This includes all vertebrates, most bilaterians, the ctenophore *M. leidyi*, and the cnidarians *Aiptasia* sp. and *H. magnipapillata* (*p* < 0.05). For example, 37.8% of *M. leidyi’*s ES-negative exons were 3n divisible, but this percentage increased up to 76.9% in ES-positive exons*.* Overall, the fraction of 3n divisible exons was higher in ES-positive exons than in ES-negative ones for 20 out of 23 animal species, and at least half of the ES-positive exons were 3n divisible in 15 out of 23 animals (Fig. 3c), suggesting that exon 3n enrichment is largely an animal feature. This enrichment was also observed when individual, rather than pooled, tissues were analyzed (Additional file 1: Figure S13A) and at different levels of sequencing depth (Additional file 1: Figure S14). Moreover, this pattern was even stronger when we restricted the analysis to highly alternative exons (*r*_*ES*_ = 30–70%; Fig. 3d). Most animals showed higher 3n enrichment in this subset, including robust increases in some non-bilaterians that did not exhibit significant 3n biases in the whole transcriptome. For example, the 3n fraction of ES-negative exons of the sponge *Amphimedon queenslandica* is 43.2%, but increases to 73.1% in highly alternative ES-positive ones (*p* = 2.55e − 3, Fisher’s exact test), and a mild increase also occurs in another sponge, *Sycon ciliatum* (*p* = 0.022; Fisher’s exact test; Additional file 1: Figure S12).

On the other hand, lack of positive 3n biases was observed in nearly all other eukaryotes, including unicellular holozoans and plants, reaching negative enrichments (*S. arctica* and the plants *Vitis vinifera* and *Physcomitrella patens*) and/or reducing this enrichment in the highly alternative subset in some species (*Oryza sativa* and *P. patens*) (Fig. 3b–d). Only the ciliate *Tetrahymena thermophila* exhibited a 3n bias akin to that of animals (*p* = 2.18e − 5, Fisher’s exact test). The lack of 3n bias in ES has been previously reported (e.g., in *Creolimax fragrantissima* [41] and *B. natans* [36]), and such ES-caused ORF disruptions were proposed to be a consequence of noisy splicing and to produce non-functional isoforms. Consistent with this idea, we found a general robust increase in the fraction of 3n exons within long genes with high expression (Fig. 3e)—a subset of ‘high-constraint’ genes that are expected to be less prone to splicing errors due to the higher energetic cost of their production [49]. We observed a 3n exon enrichment increase in high constraint genes in animals (except in poriferans and *T. adhaerens*), plants, chlorophytes, and the multicellular phaeophyte *Ectocarpus siliculosus*. However, even if high-constraint genes in non-animals showed higher fractions of 3n exons, these were not significantly biased towards being ES-positive (Fishers’ exact test, significant if *p* < 0.01; Additional file 1: Figure S12).

Overall, the 3n bias in ES events recorded in animals suggests that the lengths of alternatively spliced exons are under selective pressure to avoid ORF disruptions, possibly due to an enrichment in functional protein isoform-producing ES events. In other eukaryotes, only high-constraint genes, in which non-3n ES would be more detrimental, showed enrichments in 3n exon lengths.

### Sequence and architectural intron–exon traits influence ES and IR frequencies

To investigate how increases in ES frequencies may have taken place during animal evolution, we next studied intra-species associations between ES and IR frequencies and different genomic architectural features. Previous studies have linked the level of ES and IR within a genome to differences in traits such as the length of exons and introns, intron density, sequence composition, splicing site homogeneity, or other *cis* signals [16, 50–53]. Therefore, these associations suggest that the evolutionary processes shaping genome architecture could contribute to the variations in AS frequency across species, including the increase in ES during animal evolution.

**Fig. 4:**
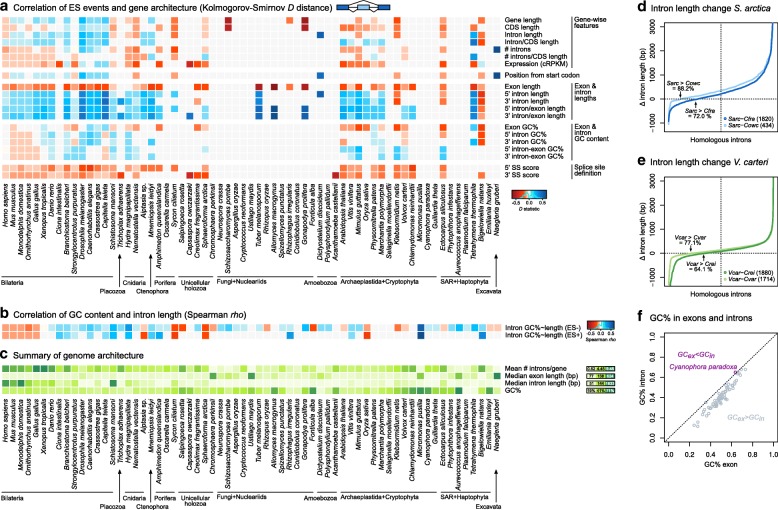
Relationship between gene structure and sequence composition and ES. **a** Heatmap representing the distance between distributions of gene architecture values for ES-positive and ES-negative exons, measured with the *D* statistic of the one-sided Kolmogorov-Smirnov two-sample test (significant if *p* < 0.01; otherwise grey). *D* values are recorded as positive/negative (*blue*/*red*) according to two one-sided Kolmogorov-Smirnov tests with complementary alternative hypotheses: positive/negative *D* (*blue*/*red*) reflects a positive/negative relationship between ES and the given feature. **b** Heatmap representing the correlation between intron length and GC content across 65 eukaryotes. Positive correlations (long, GC-rich introns, Spearman’s *rho* > 0 and *p* < 0.01) are shown in *blue*, negative correlations in *red*. **c** Heatmap representing genome-level gene architectural traits, for reference: intron density (introns/gene), median exon and intron length (bp), and GC content (percentage). **d** Difference in intron lengths (*y-*axis) between homologous sites of *S. arctica* compared to *C. fragrantissima* or *C. owczarzaki*, ranking introns according to their length differential with *S. arctica* (*x-*axis). *Black arrows* indicate the percentage of introns that are longer in *S. arctica* than in the other species (totals between brackets). **e** Difference in intron lengths (*y-*axis) between homologous sites of *V. carteri* compared to *C. reinhardtii* or *C. variabilis*, ranking introns according to their length differential with *V. carteri* (*x-*axis). *Black arrows* indicate the percentage of introns that are longer in *V. carteri* than in the other species (totals between brackets). **f** GC content in exons (*GC*_*ex*_) and introns (*GC*_*in*_) for each species in the dataset. Note that *GC*_*ex*_ > *GC*_*in*_ in all species except for the glaucophyte *Cyanophora paradoxa*

**Fig. 5:**
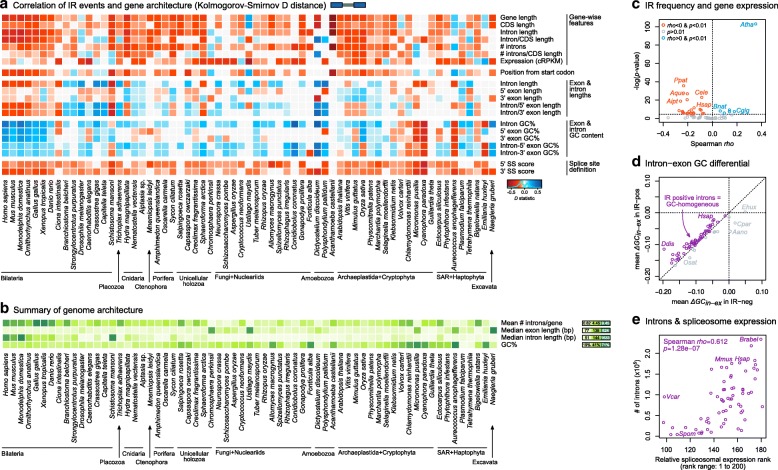
Relationship between gene structure and sequence composition and ES. **a** Heatmap representing the distance between distributions of gene architecture values for IR-positive and IR-negative introns, measured with the *D* statistic of the one-sided Kolmogorov-Smirnov two-sample test (significant if *p* < 0.01; otherwise grey). *D* values are recorded as positive (*blue*) or negative (*red*) according to two one-sided Kolmogorov-Smirnov tests with complementary alternative hypotheses. **b** Heatmap representing genome-level gene architectural traits, for reference: intron density (introns/gene), median exon and intron length (bp), and GC content (percentage). **c** Volcano plot representing the Spearman’s rank correlation coefficient (*rho*, *x*-axis) between IR frequency and transcript expression levels (cRPKM) in IR-positive genes. Significance threshold of *p* < 0.01 (*y*-axis, log-scale). **d** Mean intron–exon GC differential in IR-negative (*x*-axis) and IR-positive (*y-*axis) introns. The majority of eukaryotes lie above the diagonal (*purple*) as they have lower differentials in their IR-positive introns, i.e., IR occurs in GC-homogeneous introns. **e** Correlation between intron content (total number of introns per genome) and the relative rank of expression of the spliceosomal genes, per species (see “Methods”; Additional file 1: Figure S25)

To address this possibility, we analyzed the intron/exon structure and sequence composition of the genomic regions associated with the AS events, and compared the gene architecture of ES- and IR-positive and negative exons and introns across our set of 65 eukaryotic species. In particular, we investigated the effect of global length of genes and transcripts; the length of the alternatively spliced exons and its flanking introns (for ES) or vice versa (for IR); intronic and exonic GC content and the differential in GC content between introns and exons; the strength of the 5′ and 3′ splice site definition; the intron density (introns per gene and base pairs of introns per base pairs of coding sequence [CDS]); the relative position of the AS event along the gene (from the start codon); and the mean transcript expression level (using cRPKMs from pooled RNA-seq experiments). See “Methods” for precise definitions of each trait. Our analysis identified consistent relationships between ES, IR, and gene architecture across the eukaryotic tree of life, maintained across genomes from different lineages and robust to tissue pooling and sequencing depth (Figs. 4 and 5 and Additional file 1: Figures S13 and S15–S18).

As expected [54, 55], we identified a widespread relationship between positive cases of ES and IR and weak 5′ and 3′ splice sites (Figs. 4a and 5a; *p* < 0.01, one-sided Kolmogorov-Smirnov test with complementary hypotheses). In the case of ES, this association is significant and consistent for all species with a sufficient number of ES-positive exons, including animals, unicellular holozoans, plants, chlorophytes, the phaeophyte *E. siliculosus*, and *B. natans*. Thus, in most eukaryotes, heterogeneity in the splice sites influences ES frequencies at the intra-species level: exons with more poorly defined intron–exon boundaries are more subject to ES than those closer to the species consensus.

Another consistent association across eukaryotes was found between ES and shorter exon lengths (Fig. 4a), as previously reported for animals [47, 56]. Moreover, ES-positive exons are widely associated with longer flanking introns, both upstream and downstream. Exons with these features are expected to be spliced through ‘exon definition’, a model that proposes that the recognition of the 5′ and 3′ splice sites occurs across the exonic sequence (as intron ends are more distant); thus, interrupting this process is more likely to result in ES than in IR [50, 53]. The general positive relationship between ES and higher intron-to-exon length ratios also fits this principle, and shows a good correlation with the overall gene architecture at the species level, particularly in bilaterians (Fig. 4c), suggesting that their distinct architectures may have driven their increases in ES frequencies.

Interestingly, we observed similar patterns in the ichthyosporean *S. arctica* (with higher ES rates than other holozoans; Fig. 2e) and the chlorophyte *V. carteri* (also with higher ES rates than other chlorophytes; Fig. 2f). Their ES-positive exons had more heterogeneous splice sites than ES-negative ones, were shorter, and had higher intron-to-exon length ratios. Furthermore, the majority of *S. arctica* and *V. carteri* introns were longer than their cognates in their close unicellular holozoan (Fig. 4e) and chlorophyte relatives [38] (Fig. 4f), respectively, suggesting recent intron lengthening events. Moreover, both *S. arctica* and *V. carteri* have relatively high intron densities (3.22 and 3.88 introns/CDS kbp; Fig. 1b, c and Additional file 1: Figure S2) that derive from recent, lineage-specific intron gain processes at the root of ichthyophonid Ichthyosporea [27] and Chlorophyceae plus Trebouxiophyceae [26] (Table 1). As the median CDS length is relatively constant across eukaryotes (~ 1400 bp [57]) and is independent of intron content, higher intron densities at the species level usually imply the presence of shorter exons (Fig. 1c, Additional file 1: Figure S19). Thus, *V. carteri* and *S. arctica* seem to have independently acquired ES-conducive genome architectures (higher intron densities, shorter exons flanked by longer introns) that contribute to their ES-richer AS profiles when compared to their closest relatives (Fig. 2e, f). On the other hand, the most notable exceptions to these patterns were the multicellular phaeophyte *E. siliculosus* (which exhibited low ES frequencies despite having unusually long introns; Fig. 4c), the charophyte *Klebsormidium netis*, and *B. natans.* In these species, ES was associated with short exons, but, unusually, also with short introns (Fig. 4a).

**Table 1:** Intron mean lengths and densities in ancestral eukaryotic genomes

Ancestor	Ancestral mean intron length (bp)	Introns/CDS kbp	Introns/gene	Source intron densities
LECA	–	4.3	6.11	Csűrös et al. 2011 [26]
Uropisthokonta	328	5.1	7.25	Csűrös et al. 2011 [26]
Urholozoa	481	5.52	7.86	Grau-Bové et al. 2017 [27]
Urichthyosporea (*Chromosphaera*, *Creolimax*, *Sphaeroforma*)	263	5.53	7.86	Grau-Bové et al. 2017 [27]
Urichthyophonida (*Creolimax*, *Sphaeroforma*)	393	6.98	9.92	Grau-Bové et al. 2017 [27]
Urmetazoa (animals)	845	8.8/8.7	12.51/12.37	Csűrös et al. 2011 [26]/Grau-Bové et al. 2017 [27]
Urporifera (sponges)	451	8.63	12.27	Grau-Bové et al. 2017 [27]
Urcnidaria	1016	8.3	11.80	Csűrös et al. 2011 [26]
Urbilateria	1342	7.7/7.7	10.94/10.94	Csűrös et al. 2011 [26]/Grau-Bové et al. 2017 [27]
Urprotostomia	891	7.4	10.52	Csűrös et al. 2011 [26]
Urecdysozoa	623	7.4	10.52	Csűrös et al. 2011 [26]
Urdeuterostomia	1710	7.7	10.94	Csűrös et al. 2011 [26]
Urvertebrata	3117	7.2	10.23	Csűrös et al. 2011 [26]
Urembryophyta (land plants)	313	6.4	9.10	Csűrös et al. 2011 [26]
Urchlorophyta (all unicellular/colonial green algae)	299	3.4	4.83	Csűrös et al. 2011 [26]
Trebouxiophyceae + Chlorophyceae (*Chlorella*, *Volvox*, *Chlamydomonas*)	–	6.2	8.82	Csűrös et al. 2011 [26]

Mean intron lengths estimated from phylogenetically independent comparisons of descendant species (see “Methods”; Additional file 1: Figure S24). Intron densities (taken from [26, 27]) are reported as introns/CDS kbp and introns/gene (by multiplying by the average CDS length [1422 kbp] of all organisms in our dataset; Additional file 1: Figures S1 and S2)

Regarding IR, the ‘intron definition’ splicing model proposes the opposite scenario: impediments to across-intron recognition of splice sites can lead to IR, and this mode of splicing typically happens for short introns flanked by long exons [50, 53]. Surprisingly, however, our analysis revealed that the influence of intron and flanking exons’ length on IR is not homogeneous across eukaryotes (Fig. 5a): retained introns are indeed shorter than constitutively excluded ones in chordates (vertebrates and the tunicate *Ciona intestinalis*), *S. arctica*, *Vitis vinifera*, and unicellular algae (*Micromonas pusilla*, *Cyanophora paradoxa*, *Emiliania huxleyi*, *B. natans*, or *Guillardia theta*); but not in most other animals, unicellular holozoans, fungi, or other protists that also exhibit high IR frequencies (Additional file 1: Figure S6). The ratio of intron-to-exon length has a similarly uneven relationship with IR. Across most eukaryotes, however, introns in genes with lower intron densities (introns/gene) were generally more prone to IR, as expected. Overall, the dominance of IR in a given genome does not seem to be determined by a straightforward relationship between intron length and density. Instead, positive or negative associations can be found in a lineage- or species-specific manner.

We also used the GC content of introns (*GC*_*in*_) and exons (*GC*_*ex*_) and its differential (*ΔGC*_*in–ex*_ = *GC*_*in*_ *− GC*_*ex*_) to examine the effect of global sequence composition in AS within each species. It has been proposed that, in species with extremely long introns (e.g., mammals and *G. gallus*), a differential in GC content between exons (GC-richer) and flanking introns (AT-richer) can act as a compositional mark to assist the recognition of splice sites [52]. Considering that *GC*_*in*_ is lower than *GC*_*ex*_ in all but one eukaryote in our dataset (Fig. 4f), GC differentials in ES-positive exons could be expected to take negative values (*ΔGC*_*in-ex*_ < < 0), particularly for long introns. However, we found that this is far from being a general rule, as we observed multiple intricate associations between *ΔGC*_*in-ex*_, ES frequency, and intron length that vary across eukaryotes.

**Fig. 6:**
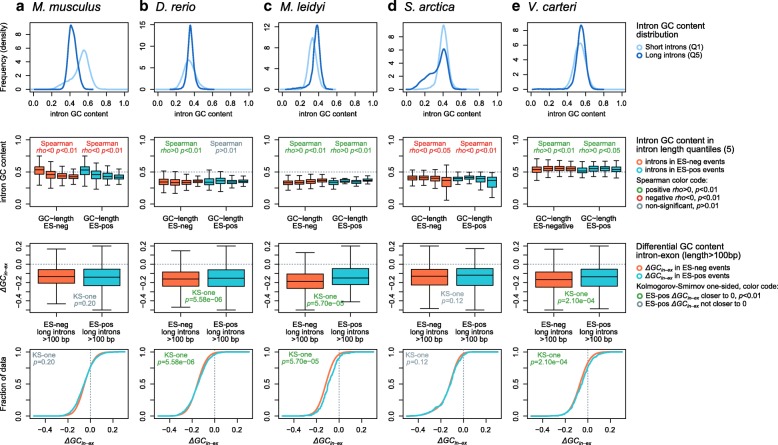
Representative patterns of the inter-relationships between GC content and AS. For selected eukaryotes (**a–e**), we plotted the distribution of GC content (percentage) in long and short introns (*first row*); the distribution of GC content in introns sorted according to their length (five quantiles, Q1–5), and ES status (negative and positive; *second row*); and the differential between intron and exon GC content in long introns (Q5) of ES-positive and ES-negative events, using box plots (*third row*) and empirical cumulative distribution functions (*fourth row*). In the *second row*, we used Spearman’s rank correlation coefficient to test the association between intron length and GC content (significant at *p* < 0.01). In the *third* and *fourth rows*, we used Kolmogorov-Smirnov one-sided test to test whether ES-positive introns had higher *ΔGC*_*in–ex*_ than ES-negative ones (i.e., *ΔGC*_*in–ex*_ ~ 0), and thus GC-homogeneous across introns and exons (significant *p* < 0.01)

First, we found that the overabundance of ES-positive exons in genic environments where *ΔGC*_*in–ex*_ < < 0 (i.e., GC-rich exons flanked by AT-richer introns) occurs only in vertebrates and the annelid *C. teleta* (Fig. 4a). In these animals, ES-positive exons were also found to be preferentially flanked by long introns (see above). However, long introns flanking ES-positive exons were only found to be AT-enriched in mammals and *G. gallus* (Spearman *rho* < 0 and *p* < 0.01; Figs. 4b and 6a, b). Second, a number of other eukaryotes (e.g., *S. ciliatum*, *S. arctica*, or *O. sativa*) exhibited a similar correlation between long and AT-rich introns genome-wide (red in Fig. 4b), but not the concomitant association between strong GC differentials (*ΔGC*_*in–ex*_ < < 0) and ES (Figs. 4a and 6e). Third, ES-positive exons in nearly all non-vertebrate eukaryotes were surprisingly biased towards regions where introns and exons have similar GC content (i.e., *ΔGC*_*in–ex*_ ~ 0; Fig. 6b–e). This latter pattern was highly unexpected, as it matches that described for IR events in human [52], which we also observed for IR for most (yet not all) eukaryotes in our dataset (Fig. 5a, d). Altogether, these results reveal complex lineage-specific interplays among GC content, intron length, and ES frequency, which cannot be generalized among eukaryotes (Fig. 6).

We have also examined the effect of whole-transcript expression levels on AS. In the majority of eukaryotes, IR-positive introns are preferentially found in lowly expressed transcripts (Fig. 5a). This result is predicted by two alternative and non-mutually exclusive hypotheses. On the one hand, IR has been widely associated with down-regulation of gene expression via NMD [16]. On the other hand, random splicing errors are more prone to affect lowly expressed genes, given its reduced fitness cost [49]. A closer inspection of IR-positive introns alone also recovered a widespread correlation between high IR rates (*r*_*IR*,*in*_) and lower expression levels in 28 out 37 species where the relationship was significant (Fig. 5c, Additional file 1: Figure S20; *p* < 0.01 and *rho* < 0 in Spearman’s rank correlation test). This result had been previously described in mammalian transcriptomes [16] and can thus be extended to all eukaryotes.

Finally, we investigated the relationship between the relative expression of core spliceosomal components and IR and ES frequency genome-wide. Since efficient splicing depends, in principle, on the sufficient expression of spliceosomal components [58, 59], we asked whether low relative expression of core factors correlated with higher ES and/or IR frequencies at the species level (Additional file 1: Figure S21A–C). Using a rank-based score to measure relative spliceosome expression (see “Methods”), we identified a negative association between spliceosome expression and IR frequency (Additional file 1: Figure S21B). This result suggests that species-wide IR levels, which do not follow a phylogenetic pattern (Additional file 1: Figure S6), could be at least partly explained by the competition among unspliced transcripts for access to the available spliceosomal machinery [58, 59] in a species- and/or sample-dependent manner. On the other hand, we found a mild, unexpectedly positive association of core spliceosomal expression with ES frequency (Additional file 1: Figure S21C). However, this result is likely due to the strong correlation found between relative spliceosomal expression and the total number of introns (Spearman’s *rho* = 0.612, *p* = 1.28e − 7; Fig. 5e) and intron density (Additional file 1: Figure S21D), which were also positively correlated with ES frequencies (Fig. 4). Thus, relative expression of core spliceosomal components seems adjusted to the number of introns to be spliced in each species.

In summary, ES events across eukaryotes were globally associated with short exons flanked by longer introns, and with weak 5′ and 3′ splice sites. Inasmuch as these features are more common in animals and plants than in most eukaryotes, we can expect higher ES frequencies in these multicellular lineages.

### Dating ES transitions in ancestral Holozoa genomes

The relationships between ES and genomic features described above were highly consistent among Holozoa (animals and their closest unicellular relatives), which suggests that these architectural and sequence effects influenced ES frequencies not only in extant organisms but in their extinct ancestors as well. We thus reasoned that these relationships could be used to predict the incidence of ES in ancestral genomes, provided that their genome architectures could be approximated.

**Fig. 7:**
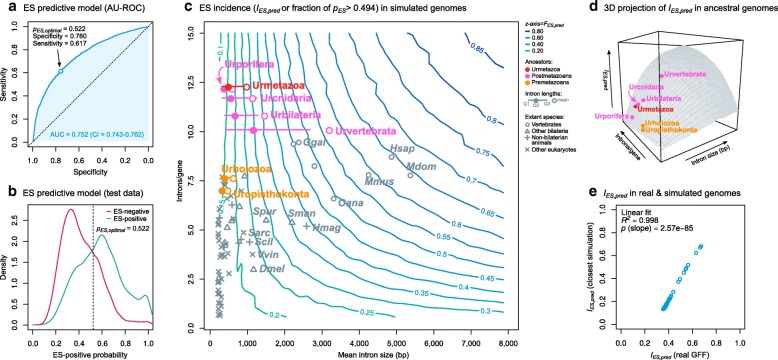
Prediction of ES incidences in ancestral genomes. **a** Receiver operating characteristic (ROC) curve of a binomial logistic regression model of ES prediction derived from 11 gene architectural features, 22 species, and a dataset of 18,678 exon skipping events for training and testing (see “Methods”). AU-ROC value (0.752) is reported with 95% confidence interval. The non-*pale blue dot* marks the optimal probability threshold (0.522) corresponding to the maximum sum of sensitivity (0.617) and specificity (0.760). **b** ES-positive probability distributions of the test dataset, containing 9318 exons with known ES status (50% positive, in *green*; 50% negative, in *maroon*). The *dotted line* marks the optimal probability threshold (0.522; see above). **c** Contour plot representing ES incidences (*I*_*ES*_, *z* axis) across 1600 simulated genomes of varying mean intron sizes (*horizontal*) and intron densities (*vertical*). *I*_*ES*_ is defined as the fraction of exons in a given simulation with *p*_*ES*_ > 0.522. *Grey marks* represent extant species positions in the intron size and density dimensions. *Red*/*pink*/*orange*-*colored dots* represent estimates of ancestral premetazoan and metazoan genomes. Ancestral intron densities from [26, 27] (Table 1); ancestral intron length distributions are represented by the mean, median, and first and third quartiles (Table 1, Additional file 1: Figure S24; see “Methods”). **d** 3D projection of **c**, with *I*_*ES*_, (*z-*axis), intron sizes (*x-*axis), and intron densities (*y*-axis). Each *dot* represents an estimation of a genome (intron density and mean intron length). **e** Correlation between the *I*_*ES*_ values obtained from analyzing real genome architectures with the predictive classifier (*x*-axis) and its closest equivalent from the spectrum of simulated genomes (*y*-axis) in terms of intron density and mean length

For this purpose, we first trained a binomial logistic regression model (see “Methods”) that classifies arbitrary exons as either ES-positive or -negative according to their overall gene architecture, assigning them an ES-positive probability (*p*_*ES*_). We used data from a selection of 24 eukaryotes with multiple animals and holozoans (Additional file 1: Figures S22 and S23) and ascertained their sensitivity and specificity by calculating the ROC curve of the classifier (Fig. 7a) and the area underneath (AU-ROC = 0.752, 95% CI = 0.743–0.762). This AU-ROC value indicates a clearly better-than-random classifier and compares with similar AS predictors previously developed for more taxonomically restricted contexts, such as IR in mammals (AU-ROC = 0.79) [16] and ES in vertebrates (AU-ROC = 0.79–0.87 in individual species) [51]. In addition, it shows that gene architecture is a consistent predictor of ES events. The highest combined sensitivity and specificity were attained when the ES-positive probability threshold (*p*_*ES*_) was set at 0.522 (Fig. 7b).

Next, we used this classifier to compare ES levels from ancestral genomes, using a topographic map of the predicted ES incidence based on genomic architectural traits (Fig. 7) from 1600 genomes simulated by combining initial assumptions of mean intron density (range 0.5–15 introns/gene) and mean intron length (range 10–8000 bp). For each simulation, we generated 10,000 internal exons with their corresponding gene architectures (see “Methods” for details) that were analyzed with the classifier to assign them an ES-positive probability (*p*_*ES*_). Then, we approximated the incidence of ES in each simulated genome by calculating the fraction of exons with *p*_*ES*_ over the optimal threshold (*p*_*ES*_ > 0.522; henceforth *I*_*ES*_). An examination of ES incidences across the spectrum of simulated ancestral genomes revealed that genomes with higher intron densities and lengths have higher *I*_*ES*_ values (Fig. 7c, d), as expected from transcriptomic analyses of extant species (Fig. 4a, b). To assess the accuracy of our simulated genomes, we compared their *I*_*ES*_ values with those obtained from the most similar real genome in our dataset (in terms of intron density and mean length), finding a linear correlation (linear fit *R*^*2*^ = 0.996, slope *p* = 1.25e − 76; Fig. 7e).

Then, we used this predictive framework to analyze ES transitions in animals by comparing the incidence of ES in reconstructed premetazoan and postmetazoan ancestors (Fig. 7c). To do so, we used previously published estimates of intron density in ancestral genomes obtained by comparative genomic analyses [26, 27], which reported an increase in intron density from 7.85 to 12.37 introns/gene between the origin of Holozoa (Urholozoa) and animals (Urmetazoa) (Table 1). As a proxy for ancestral intron lengths, we used means obtained from extant species’ phylogenetically independent contrasts [60] (see “Methods”; Additional file 1: Figure S24). Under these assumptions, the Urmetazoa (12.37 introns/gene of ~ 850 bp mean length) would have an *I*_*ES*_ ~ 0.30 (Fig. 7c). Conversely, the intron-poorer Urholozoa would reach *I*_*ES*_ ~ 0.30 only if it had an average intron of > 1000 bp, which exceeds by an order of magnitude the average intron lengths of extant unicellular holozoans (120–571 bp; Additional file 1: Figure S2) [27] and our ancestral estimation (~ 481 bp). Therefore, under reasonable assumptions for ancestral genome architecture, the unicellular ancestors of animals likely had lower ES levels than the Urmetazoa (Fig. 7c, d).

This same line of reasoning can be applied within animals. Since the Urmetazoa (12.36 introns/gene, ~ 850 bp), intron loss processes occurred in the ancestral cnidarians, poriferans, bilaterians, and vertebrates (Table 1) [26, 27]. Therefore, to sustain an increase in ES levels relative to the Urmetazoan, these lineages should have undergone intron lengthening processes. This was probably the case already in early bilaterians (*I*_*ES*_ ~ 0.40 if mean intron length ~ 1300 bp) and, with higher certainty, in vertebrates (*I*_*ES*_ ~ 0.60 if mean intron length ~ 3100 bp; Fig. 7c, d). On the other hand, our results do not support bilaterian-like ES enrichments in the early branching animal ancestors Urcnidaria and Urporifera: although they had high intron densities, they likely had shorter introns compared to the Urbilaterian (Table 1), thus yielding lower *I*_*ES*_ values (Fig. 7c, d).

Therefore, we hypothesize that the moderately high ES in certain non-bilaterian eukaryotes appear concomitantly with recent, species-specific changes in their gene architecture. One possible example is *H. magnipapillata*, which has undergone a recent, intra-genus genome size expansion [61, 62] that could help explain its enlarged introns (Fig. 1b). Similarly, ichthyosporeans attained high intron densities independently of animals [27] (Table 1), and the longer introns found in species like *S. arctica* appear to have been recently acquired too (Fig. 4d). Altogether, prediction of ES incidences from ancestral reconstructions and comparison with extant species are consistent with the increase in ES frequency observed in metazoans, particularly from bilaterian ancestors.

## Discussion

We performed a comparative survey of AS frequencies in 65 eukaryotes to understand the evolutionary dynamics of this layer of gene regulation. Our analysis revealed that ES and IR events can be found, at varying frequencies, in transcriptomes from all major eukaryotic groups (Opisthokonta, Amoebozoa, Virdiplantae and Cryptophyta, SAR and Haptophyta, and Excavata). This result thus provides further support for an early emergence of dual AS—i.e., involving both ES and IR—in eukaryotes [18, 33, 34]. Given the generally higher prevalence of IR in all eukaryotic super-groups (Fig. 2a, b, Additional file 1: Figure S6), LECA likely exhibited an IR-dominated AS profile as well.

Since its early origin in LECA, the incidence of ES has varied between different eukaryotic groups. Our analysis of ES frequencies across multiple species revealed higher ES frequencies in multicellular animals (particularly vertebrates and other bilaterians) and, to a lesser extent, plants. While most other eukaryotes maintain lower ES levels, a number of punctual exceptions have surfaced—e.g., the ichthyosporean *S. arctica* or the colonial chlorophyte *V. carteri* [38] (Fig. 2). Conversely, other major lineages like fungi or amoebozoans exhibit comparatively low ES levels.

In most ES-rich eukaryotes, we identified a set of gene architectural features that influence the frequency of AS at the gene level: the heterogeneity of 5′ and 3′ splice sites, the length of exons and their flanking introns, and its correlate regarding intron density. These architectural traits were globally coherent across eukaryotic lineages, especially in animals and plants (Fig. 4a). In particular, the mode of splicing by ‘exon definition’, which posits that short exons flanked by long introns are enriched in ES events [50, 53], is relevant in most eukaryotic transcriptomes with sufficient ES levels analyzed here, except for *E. siliculosus* and *B. natans*. These properties are more common in intron-rich organisms with heterogeneous splice sites [18, 34]. Overall, this result suggests a non-deterministic ‘soft code’ that influences ES rates across eukaryotic lineages.

The existence of a pan-eukaryotic ES ‘soft code’ implies that inter-specific changes in ES levels can be associated with the evolutionary histories of their underlying genomic traits. Since the origin of intron-rich genomes in LECA, the evolutionary lineages leading up to ES-rich animals and plants always maintained high intron densities, and later underwent secondary intron gain processes [26, 27, 63] concomitant with ES transitions (Fig. 2). Such intron gain episodes had a direct effect on exon length: given that the mean CDS length is relatively constant across eukaryotes [57] and that intron content is independent of CDS length (Additional file 1: Figure S24), genomes affected by long-term intron gain processes have shorter exons (Fig. 1c, Additional file 1: Figure S24). This is likely a direct consequence of the most common mechanisms of de novo intron creation, which involve the insertion of new intronic sequences splitting pre-existing exons [64–68]. Furthermore, most extant intron-bearing eukaryotic genomes maintain heterogeneous splice sites [18, 34], a conserved core spliceosomal machinery [31] (Additional file 1: Figure S21), and a diverse complement of splicing factors [27, 32], all of which were already present in LECA. Globally, the evolutionary dynamics of these genomic traits are consistent with our results: the early origin of the genetic machinery (core spliceosome) and structure (intron-rich genomes with diverse splice sites) fits the ancestral emergence of ES in LECA and its widespread incidence, and subsequent lineage-specific changes in genome architectures would have paved the way for the evolution of higher ES frequencies.

Furthermore, if the effect of gene architecture on ES is widespread and coherent across eukaryotes, we can infer that it was also relevant in their ancestors. Thus, we took advantage of the pan-eukaryotic ‘soft code’ of ES determination to investigate the timing of ES transitions in animals and their unicellular ancestry. Specifically, we quantified the relationship between gene architecture and ES on extant eukaryotes and projected these effects into the past with a predictive framework (Fig. 7c, d). We propose an early ES enrichment in the Urmetazoa concomitant with the origin of multicellularity, followed by further enrichment in bilaterians and vertebrates. The ES transition in early animals would come as a consequence of changes in genome architecture, as large genomes with high intron densities and long intronic segments became more common in animals [26, 27, 69] than in their unicellular holozoan relatives [27, 57, 70–72].

Remarkably, most animals showed high fractions of 3n exons among their ES events, but this was not observed in plants or other eukaryotes (Fig. 3b, c). Thus, the pressure to maintain ORFs in the event of ES seems an animal-specific trait. Interestingly, even animals with lower ES frequencies (such as non-bilaterians) or that have secondarily simplified intron–exon architectures (e.g., *C. elegans* and *D. melanogaster*) often have strong 3n biases in their ES profiles. Thus, it is conceivable that a protein isoform-enabling 3n bias already existed in the Urmetazoa, before the major increase in ES frequency occurred in bilaterians (Figs. 2 and 7). If so, these increases were likely more easily recruited to functionally diversify their proteomes, perhaps contributing to the selection of more ES-prone genome architectures, establishing a positive feedback loop. Moreover, it is possible that other regulatory features that are characteristic of bilaterians, such as the high prevalence of long-range enhancer–promoter interactions [73], may have also contributed to longer intron sequences and thus to more ES-prone genome architectures.

On the other hand, neither unicellular holozoans (Fig. 2) nor the unicellular ancestors of animals (Fig. 7) seem to have had ES-rich transcriptomes with 3n exon biases. Therefore, AS-mediated isoform production was a largely irrelevant phenomenon during the unicellular ancestry of animals (since the Urholozoa to the origin of multicellularity), an evolutionary period that was otherwise fecund in other sources of gene innovation [27].

## Conclusions

We find that the influence of gene architectural traits in the frequencies of IR and ES is globally conserved across all eukaryotic lineages. Thus, gene architecture (i.e. the lengths of introns and exons, splice site definition, intron density, etc.) is the basis of a ‘soft’ pan-eukaryotic cis-regulatory code for AS determination that affects both extant and ancestral genomes. This result emphasizes the effect of long-term genome evolutionary patterns in shaping AS, a fast-changing transcriptome regulatory layer. In that regard, we identify multiple ES transitions coinciding with the evolution of ES-favourable genome architectures – e.g. in animals and plants, but also in more restricted taxonomic contexts such as the ichthyosporean *S*. *arctica*.

Our taxon-rich analysis confirms that animal transcriptomes have a unique AS profile. Quantitatively, extant animals exhibit the highest ES frequencies among eukaryotes as a consequence of cumulative ES enrichments in the Urmetazoa and, above all, the Urbilateria. Furthermore, from a qualitative perspective, the earliest animals became enriched in frame-preserving ES events, which is essential for widespread isoform-mediated proteome diversification. Thus, our observations suggest that the unparalleled increase in ES frequencies of modern bilaterians (including vertebrates) is a consequence of the interplay between the complexification of animal genome architectures, on one hand, and the co-option of ES events for regulated proteome expansion, on the other.

## Methods

### Sources of genome and transcriptome data

We assembled a dataset consisting of genome assemblies and annotations from 65 eukaryotic species for which high-coverage Illumina RNA-seq data were already available or for which we generated de novo data (Additional file 1: Figure S1 and below). We retrieved the genomic coordinates of genes, transcripts, and exon sequences for each genome from associated GFF annotation files. If more than one isoform per gene was annotated, the longest CDS was considered to be the canonical transcript (a proxy with ~ 90% correspondence with proteomics-driven main isoform selection [74]). In order to homogenize the experimental procedures used to build each RNA-seq library, we used (i) poly(A)-selected libraries only, (ii) either single-end or the forward reads of paired-end data, and (iii) trimmed reads to a length of 50 bp if they were longer (using FASTX Toolkit [75]). In species where RNA-seq experiments included more than one sample (replicates, time series, growth conditions, etc.), all reads were pooled into a single FASTQ file.

In the case of *N. gruberi*, *S. arctica*, and *T. adhaerens*, new RNA-seq datasets were produced and deposited in the European Nucleotide Archive (ENA) under the accession codes PRJEB23822, PRJEB23831, and PRJEB23829, respectively. RNA extractions were performed from confluent axenic cultures of mixed cells (*N. gruberi*, ATCC 1034 medium, modified PYNFH at 30 °C; *S. arctica*, marine broth medium Difco 2216 at 12 °C) or whole organisms (*T. adhaerens*, artificial sea water at 25 °C). RNA was extracted using Trizol reagent (Life Technologies, Carlsbad, CA, USA) with a further step of Dnase I (Roche) to avoid contamination by genomic DNA, then purified using RNeasy columns (Qiagen). We sequenced paired-end libraries of 50 (*N. gruberi*, 276,095,412 reads; *S. arctica*, 218,701,756 reads) or 125 bp (*T. adhaerens*, 84,583,746 reads) with an insert size of 250 bp. Libraries were constructed using the Trueseq Sequencing Kit v4 (Illumina, San Diego, CA, USA). The libraries were sequenced in one lane of Illumina HiSeq 2000 (*N. gruberi*, *T. adhaerens*) or 2500 (*S. arctica*) at the CRG genomics facility (Barcelona).

### Detection and quantification of exon skipping and intron retention events

We adapted and expanded the computational framework previously developed [36, 51] to detect and quantify ES and IR (graphical summary in Additional file 1: Figure S3), as follows.

#### Exon skipping detection

For each group of three consecutive exons in the genome (exon triplet), we built a composite of exonic junctions consisting of (i) 42 bp from the 5′ end of the first exon and 42 bp from the 3′ end of the second exon (E1–E2 junction); (ii) 42-bp fragments from the 5′ end of the first exon and the 3′ end of the third exon (E1–E3); and (iii) 42-bp fragments from the 5′ end of the second exon and the 3′ end of the third exon (E2–E3). Hence, each triplet consisted of two inclusion junctions (E1–E2 and E2–E3) and one that skipped the middle exon (E1–E3). If any exon was shorter than 42 bp, the entire length of the exon was used, and the resulting junction sequence would be shorter than 84 bp.

Then, we computed the effective mappability of each junction in order to exclude exon–exon boundaries where RNA-seq mapping would be unreliable [76]. Specifically, we (i) built an artificial RNA-seq library consisting of all the possible reads derived from each junction in a 50-bp sliding window; (ii) mapped these reads to the original junctions using *bowtie* v1.1.2, allowing a maximum of two mismatches (*−v 2*) and no multiple alignments (*−m 1*) [77]; and (iii) removed all junction triplets for which at least one triplet had < 20 effectively mappable positions (maximum is 35 for 50 bp reads, and ≥ 8 positions mapped from each exon). Then, we aligned the pooled RNA-seq libraries to the remaining exon triplets with *bowtie* and the same parameters as above. We corrected the number of mapped reads by dividing the read counts by the ratio obtained from dividing the mappable positions of that junction (20–35 bp) and the maximum theoretical mappability (35 bp).

The ES rate of each middle exon (*r*_*ES*_) was then computed as follows:

$$ {r}_{ES}=\frac{m_{E1E3}}{\left({m}_{E1E2}+{m}_{E2E3}\right)/2+{m}_{E1E3}} $$

where *m* denotes the mappability-corrected number of reads mapping in the E1–E2, E2–E3, and E1–E3 junctions. We classified exon junctions into three categories: (i) ES-positive if *m*_*E1E2*_ + *m*_*E2E3*_ + *m*_*E1E3*_ > 10, *m*_*E1E2*_ > 2, *m*_*E2E3*_ > 2, *m*_*E1E3*_ > 1, and *r*_*ES*_ ≥ 10% but < 90%; (ii) ES-negative if *m*_*E1E2*_ + *m*_*E2E3*_ + *m*_*E1E3*_ > 10, *m*_*E1E2*_ > 2, *m*_*E2E3*_ > 2, *m*_*E1E3*_ ≥ 0 but *r*_*ES*_ < 10%; and (iii) non-classifiable if any condition was not fulfilled.

#### Intron retention detection

For each intron of the genome, we built three junction sequences consisting of (i) 42 bp from the 5′ end of the first exon and 42 bp from the 3′ end of adjoining intron (E–I junction); (ii) 42-bp fragments from the 5′ end of the first exon and the 3′ end of the second exon (E–E); and (iii) 42 bp fragments from the 5′ end of the intron and the 3′ end of the second exon (I–E). Hence, each intron triplet had one spliced junction (E–E) and two retention junctions that spanned the intron ends (E–I and I–E), all of them 84 bp long (or less, if any exon or intron was shorter than 42 bp).

The mappability of each exon–intron junction was computed as specified above for ES junctions, also discarding cases with < 20 effectively mappable positions. We then aligned the same pooled RNA-seq data to the remaining exon–intron junctions using *bowtie*, and corrected the number of mapped reads.

The IR rate of each intron (*r*_*IR*_) was computed as follows:

$$ {r}_{IR}=\frac{\left({m}_{IE}+{m}_{EI}\right)/2}{m_{EE}+\left({m}_{IE}+{m}_{EI}\right)/2} $$

where *m* denotes the mappability-corrected number of reads mapping in the I–E, E–I, and E–E junctions. Finally, we classified intron junctions in three categories: (i) IR-positive if *m*_*IE*_ + *m*_*EI*_ + *m*_*EE*_ > 10, *m*_*IE*_ > 1, *m*_*EI*_ > 1, *m*_*EE*_ > 2, and *r*_*IR*_ ≥ 10% but < 90%; (ii) IR-negative if *m*_*IE*_ + *m*_*EI*_ + *m*_*EE*_ > 10, *m*_*IE*_ ≥ 0, *m*_*EI*_ ≥ 0, *m*_*EE*_ > 2, and *r*_*IR.in*_ < 10%; and (iii) non-classifiable if any condition was not fulfilled.

#### ES and IR detection pipeline quality control

With the aim of identifying biases in our computational pipeline, we assessed the fraction of all potential exon–exon and exon–intron junctions that passed the mappability filters and were used in the ES and IR detection procedures (Additional file 1: Figure S25). Putative systematic biases derived from species-specific genome architectural traits (paralogy, exon/intron lengths, repetitive genomes) could affect RNA-seq mapping in certain species and thus bias downstream analyses.

We analyzed the fraction of junctions with > 20 effective mappable positions (mappability filter, Additional file 1: Figure S25A, B), finding no evidence of taxonomic biases in any particular eukaryotic group. A few phylogenetically unrelated species exhibited low survival rates (e.g., *Selaginella moellendorffi* and *E. huxleyi*). This general mappability filter was then decomposed in its different components. First, we assessed the fraction of exons and introns from each genome that had the minimal required length to survive the mappability filter (i.e., ≥ 27 bp), finding no bias in any species (Additional file 1: Figure S25C, D). Next, we examined which mapping filters (i.e., *bowtie* multiple mapping [−m 1] or excess of mismatches [−v 2]) caused ES or IR junction removal in each species (Additional file 1: Figure S25E, F), finding that peaks in *S. moellendorffi* and *E. huxleyi* were mostly due to multiple mapping. Multiple mapping is likely caused by abundant recent gene duplications in these two species. Indeed, both *S. moellendorffi* and *E. huxleyi* had a high fraction of intron-bearing genes with recent paralogous sequences (> 99% or > 95% amino acid sequence identity, and > 90% reciprocal alignment coverage, calculated with *diamond* [78]; Additional file 1: Figure S25G, H). Finally, we found that some species also had a relatively high number of uncalled bases (N) in their exon–exon or intron–exon junctions (most notably *O. sativa* but also *S. moellendorffi*; Additional file 1: Figure S25I, J), which can partly explain the lower survival rates in these species after the mappability filter (Additional file 1: Figure S25A, B).

Overall, these quality control analyses show that the compounded effect of genome architectural traits (recent duplication, uncalled bases, and repetitive sequences hindering RNA-seq mapping) only affected individual species and did not systematically affect ES or IR detection in any large group of eukaryotes.

### Transcriptome-wide quantification of AS levels

In order to measure the average frequency of ES at the species level, we divided each species’ set of classifiable exon triplets into 100 bins of 100 triplets, and calculated the per-triplet frequency of ES from 10,000 randomly chosen reads for each bin (selected among those mapping to the in bin’s exon–exon junctions, or a mean sequencing depth of ~ 20×). The average ES rate of each bin *i* (*r*_*ES*,*i*_) was recorded to obtain a species-level distribution of ES frequencies (*F*_*ES*,*sp*_). An analogous measurement was used to calculate the distribution of species-level IR frequencies (*F*_*IR*,*sp*_) from 100 bins, 100 triplets, and 10,000 reads per bin (mean sequencing depth ~ 20×). This process is summarized in Additional file 1: Figure S3C. It should be noted that comparisons among *F*_*IR*,*sp*_ are more susceptible to technical variability derived from library preparation than those among *F*_*ES*,*sp*_, since differences in the efficiency in poly(A)+ selection are only expected to significantly affect *F*_*IR*,*sp*_ estimates.

Statistical comparisons of poolings of species-level *F*_*ES*,*sp*_ distributions (Fig. 2c–f) were done using one-sided Kolmogorov-Smirnov tests. Differences between intra-species replicates (Additional file 1: Figure S7) were assessed with Wilcoxon rank-sum tests. All statistical contrasts were done with R *stats* [79].

ES frequency analysis was re-assessed by using (i) downsampled RNA-seq experiments (to 2×, 5×, 10×, and 15× sequencing depths, by random read selection; Additional file 1: Figure S10); and (ii) individual tissue-level samples from selected animal (*H. sapiens*, 2× *M. musculus*, *M. domestica*, *O. anatinus*, *G. gallus*, *X. tropicalis*, *D. rerio*, *S. ciliatum*) and plant species (*A. thaliana* and *M. polymorpha*) (Additional file 1: Figure S13A). Original sequencing depths in the ES and IR triplets junctions are available in Additional file 1: Figure S25K, L.

### Analysis of gene features: architecture, splice sites and expression levels

For each exon or intron analyzed, we recorded sequence and architectural parameters at the gene and AS event levels. At the gene level, we studied the following parameters: gene length, CDS length (all exons), total number of introns in the gene (intron density), position of the exon/intron within the gene sequence (base pairs from starting codon), and total length of all introns with respect to exons (ratio). At the AS event level, we recorded the length of the individual exon and flanking introns (for ES) or intron and flanking exons (for IR), and the ratio between them (intron/exon lengths); the GC content of exons and flanking introns (for ES) or introns and flanking exons (for IR), and the differential between them (*ΔGC*_*in–ex*_ = *GC*_*in*_ *–GC*_*ex*_), and a boolean variable describing whether the length of the alternative exon/intron was divisible by three (1 = true, 0 = false). These features were derived from the GFF annotation and genome sequence (data sources in Additional file 1: Figure S1).

In addition, we analyzed the conservation degree of 5′ and 3′ splice sites when compared to species-specific consensus. For each species, we built position-weighted matrices (PWM) from the alignments of all 3′ (23 bp, 20 from the intron and 3 from the exon) and 5′ (9 bp, 3 from the exon and 6 from the intron) splice sites using the consensus matrix function in the *Biostrings* R library [80]. Then, for each individual splice site in the genome, the distance from the PWM consensus was calculated. Splice sites were delimited as in [81].

Finally, we evaluated transcript expression levels using the mappability-corrected RPKM metric (cRPKM), aligning the pooled RNA-seq data for each species to the genome-predicted transcript sets using *bowtie* (longest transcript per gene only, see above) and calculating transcript-specific effective mappabilities as detailed above [76].

### Statistical analysis of AS frequency and gene features

For each species and for each of the quantitative sequence and architectural features listed above, significant differences between the values taken by the IR-/ES-positive triplets and the IR-/ES-negative triplets were evaluated using two independent one-sided Kolmogorov-Smirnov two-sample tests with complementary alternative hypotheses: first, we tested whether the empirical cumulative distribution of the IR-/ES-positive events lied above the IR-/ES-negative events’ values (signaling a positive relationship between IR/ES and the given feature); second, we tested whether it lied below (i.e., for a negative relationship). We used the Kolmogorov-Smirnov distance (*D* statistic) to measure the distance between distributions. *D* was recorded as positive if *p* < 0.01 in the first test, negative if *p* < 0.01 in the second one, or as NA if it was not significant in any test or contradictorily significant. The resulting matrix was plotted using the *heatmap.2* function in the R *gplots* v3.0.1 library [82].

To further investigate the relationship between gene expresion levels and IR, we also tested the significance of monotonic correlations between cRPKMs and AS rates using Spearman’s rank correlation coefficient (*rho*, significant for *p* < 0.01).

Finally, we tested if the frequency of 3n divisible lengths in IR-/ES-positive events significantly differed from that of IR-/ES-negative (i.e., constitutive) events using Fisher’s exact test (significant for *p* < 0.01, except if otherwise stated). All statistical analyses were done with R *stats* library [79].

The relationships between gene architectural traits and ES and IR events were re-assessed by using (i) downsampled RNA-seq experiments (to 2×, 5×, 10× and 15× sequencing depths, by random read selection; Additional file 1: Figures S17 and S18); and (ii) individual tissue-level samples from selected animal (*H. sapiens*, *M. musculus*, *M. domestica*, *O. anatinus*, *G. gallus*, *X. tropicalis*, *D. rerio*, *S. ciliatum*) and plant species (*A. thaliana* and *M. polymorpha*) (Additional file 1: Figure S13B, C). Original sequencing depths in the ES and IR triplets junctions are available in Additional file 1: Figure S25K, L.

### Prediction of ES incidence using gene architectural features

Using our binary classification of positive/negative ES events, we created a binomial logistic regression model for a selection of representative eukaryotes with high ES frequencies. First, we selected 18,678 events with known gene architecture (devised to include an equal number of positive and negative events) from 24 representative eukaryotic species (Additional file 1: Figure S22). We used (i) 12,452 positive and negative ES events (two-thirds of the dataset) as the binary-dependent variable, and (ii) 11 quantitative gene traits and a Boolean factor indicating 3n divisibility as independent predictors (Additional file 1: Figure S22). The binomial logistic regression was built using the generalized linear model function from R *stats* library [79]. The predictive performance of each model was estimated with the area under its corresponding ROC curve (AU-ROC), calculated using an independent test subset (6226 events, one-third of the dataset) with the *pROC* R library [83]. An optimal probability threshold was selected by maximizing the sum of specificity and sensitivity (*p*_*ES*,*optimal*_ = 0.522). We assessed the significance of the model’s coefficients with the Z-statistic significance according to the Wald test and its corresponding ANOVA deviance table with sequential Chi-square tests (Additional file 1: Figure S22).

The predictive model of ES was applied to a set of 1600 simulated genomes with varying intron densities (0.5–15 introns/gene range, 40 regular intervals) and mean intron sizes (10–8000 bp range, 40 intervals at cubic distances). Each simulated genome contained 20,000 genes of which at least 10,000 were multi-exonic (depending on its input intron density).

For each simulated genome, gene architectures were drawn from the empirical distributions derived from 10,000 randomly selected genes from each of the 30 representative eukaryotes. Specifically, we used log-normal distributions for the lengths of CDS (mean = 1422 bp), 5′ and 3′ introns (mean = input mean intron length), genes (mean = CDS length + input mean intron length × input mean intron density), and exons (mean = CDS length/number of introns per gene); and normal distributions of 5′ splice site (mean = empirical) and 3′ splice site (mean = empirical) scores. For all normal or log-normal distributions, the standard deviations were obtained from the empirical distributions. See Additional file 1: Figure S23 for a complete report of means and standard deviations for each distribution and a list of species employed. All variables were estimated using maximum-likelihood fitting of univariate distributions as implemented in the *fitdistr* utility of the R *MASS* library [84], and each set of simulated gene architectures was built using the normal or log-normal distributions implemented in R *stats* [79].

Then, from each simulated genome, we selected up to 10,000 random internal exons (using R *mosaic* library [85]) without replacement and analyzed them with the binomial logistic regression model to obtain exon-wise ES-positive probabilities (*p*_*ES*_). In simulated genomes with low intron densities, the number of internal exons (i.e., with two flanking introns) was sometimes less than 10,000 (for the lower bound of 0.5 introns/gene, 7864 internal exons were retrieved on average).

To estimate the incidence of ES across the spectrum of simulated genomes, we calculated the fraction of exons in each simulation with *p*_*ES*_ > 0.522 (the optimal probability threshold according to the ROC curve with real test data), or *I*_*ES*_. The relationship between *I*_*ES*_, mean intron size, and intron density was investigated using a contour map and its corresponding 3D projection, produced with the R *graphics* [79] and *akima* libraries [86], respectively.

Then, we overlaid the resulting contour map with estimations of ancestral genomes’ intron density and length distributions. Ancestral intron densities were obtained from [26, 27] (Table 1). Ancestral intron length distributions were estimated using phylogenetically independent contrasts (PIC) [60, 87] as implemented in the R *ape* library [88]. Specifically, we calculated mean, median, and first and third quartiles of the ancestral intron length distributions, using PIC analysis of the descendant nodes (e.g., Urcnidaria median value corresponds to the phylogeny-controlled medians of the three extant cnidarians in our dataset; available as Additional file 1: Figure S24). In order to account for the phylogenetic relationships as required in PIC analysis [60, 87], we built a phylogenetic tree of the 65 eukaryotes in our dataset with 429 single-copy pan-eukaryotic orthologs from the BUSCO database [89] with IQ-TREE v1.5.1 [90]. For each of the 429 BUSCO orthologs and 65 organisms, we searched the best-matching protein in each predicted proteome with *hmmsearch* [91], which were aligned with MAFFT v7.245 (L-INS-i algorithm with up to 1000 refinement iterations) [92] and trimmed with the trimAL automated algorithm [93]. Then, we concatenated all 429 trimmed alignments in a multi-gene alignment (149,809 amino acid positions) that was analyzed with IQ-TREE using the LG + G4 model and a constrained phylogenetic tree as a reference (manually built from previous phylogenomic analyses [27, 94–97]; see Additional file 1: Figure S24).

### Length distribution of homologous introns in holozoans and chlorophytes

To test whether *S. arctica* and *V. carteri* have lengthened or shortened their introns, we compared the length distributions of one-to-one homologous introns between them and their close relatives. First, we built two databases of orthologous genes: for unicellular holozoans (using predicted proteins of *S. arctica* and *C. fragrantissima* plus *C. owczarzaki* as outgroup) and chlorophytes (using *V. carteri* and *C. reinhardtii*, plus *Chlorella variabilis* as outgroup), using in both cases Orthofinder v2.1.2 [98] with MCL inflation = 2.1 [99]. Please note that, although *C. perkinsii* (ichthyosporean) is a closer outgroup for the holozoan analysis than *C. owczarzaki* (filasterean), the former species’ intron contents are heavily reduced, whereas the latter shows a remarkable level of intron site conservation despite the high phylogenetic distance with ichthyosporeans [27].

For each database of orthologous genes, we retrieved the transcript sequences of all single-copy orthologs present in the three species, onto which we mapped the in-transcript coordinates of all annotated introns and their length (bp). For each three-gene group, we retrieved 40-bp-long transcript segments around each intron site (20 bp upstream + 20 bp downstream) using Bedtools v2.24.0 [100], and aligned them locally with *blastn* [101] to identify introns inserted in homologous transcript regions (using *-task blastn-short*). We considered as homologous introns those alignments that fulfilled these conditions: (i) alignment length > 8 bp; and (ii) alignment spanned at least 4 ungapped bp up/downstream the intron insertion (20th position). Then, we analyzed the intron length distribution of homologous introns in pairwise species comparisons.

### Conservation and expression of the core spliceosomal components

We surveyed the translated proteomes of each eukaryotic species in our dataset to identify bona-fide orthologs of 82 core spliceosomal components. Specifically, we annotated the KEGG orthologous groups [102] of each species using eggNOG mapper [103, 104] and identified the KEGG orthologs (KO) corresponding to the spliceosomal snRNPs, U1, U2, U4/U6-U5 complexes, and the Prp19 complex (Additional file 1: Figure S21). The resulting matrix of KO presence/absence per species was plotted using the *heatmap.2* function of the R *gplots* v3.0.1 library [82], using the eggNOG annotation bitscore values (normalized to the 0–1 range within each KO) as a visual reference of sequence conservation. Then, we analyzed the relative level of expression of the spliceosomal components in each species. Specifically, we used a rank-based score that reflected whether the spliceosomal components were more or less expressed relative to other genes in that species’ RNA-seq sample. The species-level relative rank expression of the spliceosome was calculated as follows: (i) we selected 500 random subsets of 199 genes (sampling with replacement); (ii) we sequentially added each of the 82 spliceosomal genes to the subset (totaling 200 genes per subset); (iii) each gene was assigned a rank score ranging from 1 (lowly expressed within the subset) to 200 (highly expressed); (iv) we recorded the rank score of the 82 spliceosomal genes within each random gene subset; and (v) calculated the relative rank of spliceosome expression per species by averaging the ranks of its 82 components (or those present) across all 500 random gene subsets.

## Additional files

Additional file 1: **Figures S1–25.** Data sources, methods overview, complete reporting of statistical analyses, and replicate/technical analyses. (PDF 2453 kb)
