# Supplementary material for: Origin of exon skipping-rich transcriptomes in animals driven by evolution of gene architecture
Source: Genome Biol. 2018 Sep 17;19:135. doi: 10.1186/s13059-018-1499-9 (PMC6142364; doi:10.1186/s13059-018-1499-9)
Supplement: Supplementary file 1 — Figures S1–25. Data sources, methods overview, complete reporting of statistical analyses, and replicate/technical analyses. (PDF 2453 kb) [file 13059_2018_1499_MOESM1_ESM.pdf]

Species	Abbr.	Genome sequence and annotation	Transcriptomes	Taxonomy				
Homo sapiens	Hsap	Ensembl 78	SRP007412 / Brawand 2012	Vertebrata (chordate)	Metazoa	Holozoa	Opisthokonta	Amorphea
	Hsax		SRP056969 / Duff 2015					
Mus musculus	Mmus	Ensembl 78	SRP007412 / Brawand 2012	Vertebrata (chordate)	Metazoa	Holozoa	Opisthokonta	Amorphea
	Mmux		SRP015997 / Barbosa-Morais 2012					
Monodelphis domestica	Mdom	Ensembl 78	SRP007412 / Brawand 2012	Vertebrata (chordate)	Metazoa	Holozoa	Opisthokonta	Amorphea
Ornithorhynchus anatinus	Oana	Ensembl 78	SRP007412 / Brawand 2012	Vertebrata (chordate)	Metazoa	Holozoa	Opisthokonta	Amorphea
Gallus gallus	Ggal	Ensembl 78	SRP007412 / Brawand 2012	Vertebrata (chordate)	Metazoa	Holozoa	Opisthokonta	Amorphea
Danio rerio	Drex	Ensembl 78	SRP048807	Vertebrata (chordate)	Metazoa	Holozoa	Opisthokonta	Amorphea
Xenopus tropicalis	Xtro	Ensembl 78	SRP012375 / Tan 2012	Vertebrata (chordate)	Metazoa	Holozoa	Opisthokonta	Amorphea
	Xtrx		SRP015997 / Barbosa-Morais 2012					
Ciona intestinalis	Cint	Ensembl 78	SRP042651	Tunicata (chordate)	Metazoa	Holozoa	Opisthokonta	Amorphea
Branchiostoma belcheri	Brabel	PRJNA214454	SRP025148	Urochordata (chordate)	Metazoa	Holozoa	Opisthokonta	Amorphea
Strongylocentrotus purpuratus	Spur2	Ensembl Metazoa 27	GSE97267 / Burguera 2017	Echinodermata	Metazoa	Holozoa	Opisthokonta	Amorphea
Drosophila melanogaster	Dmel	Ensembl Metazoa 27	SRP001696 / modENCODE 2011	Ecdysozoa	Metazoa	Holozoa	Opisthokonta	Amorphea
	Dmel2		SRP082392 / Katzenberger 2016					
Caenorhabditis elegans	Cele	Ensembl 78	SRP000401	Ecdysozoa	Metazoa	Holozoa	Opisthokonta	Amorphea
Crassostrea gigas	Cgig	Ensembl Metazoa 27	SRP014559	Lophotrochozoa	Metazoa	Holozoa	Opisthokonta	Amorphea
Capitella teleta	Ctel	Ensembl Metazoa 27	SRP102138	Lophotrochozoa	Metazoa	Holozoa	Opisthokonta	Amorphea
Schistosoma mansoni	Sman	Ensembl Metazoa 27	ERP000427	Lophotrochozoa	Metazoa	Holozoa	Opisthokonta	Amorphea
Trichoplax adhaerens	Tadh	Ensembl Metazoa 27	**This study – PRJEB23829**	Placozoa	Metazoa	Holozoa	Opisthokonta	Amorphea
Hydra magnipapillata	Hmag	NCBI PRJNA12876	SRP051110	Cnidaria	Metazoa	Holozoa	Opisthokonta	Amorphea
Nematostella vectensis	Nvex	Ensembl Metazoa 27	SRP021895 / Schwaiger 2014	Cnidaria	Metazoa	Holozoa	Opisthokonta	Amorphea
	Nvec		SRP018739 / Helm 2013					
Aiptasia sp.	Aipt	(Baumgarten et al. 2015)	SRP047443	Cnidaria	Metazoa	Holozoa	Opisthokonta	Amorphea
Mnemiopsis leidyi	Mlei	Ensembl Metazoa 27	SRP014828	Ctenophora	Metazoa	Holozoa	Opisthokonta	Amorphea
Amphimedon queenslandica	Aque	Ensembl Metazoa 27	SRR1511618	Porifera	Metazoa	Holozoa	Opisthokonta	Amorphea
Oscarella carmela	Ocar	(Nichols et al. 2012)	SRR1042012	Porifera	Metazoa	Holozoa	Opisthokonta	Amorphea
Sycon ciliatum	Scil	(Fortunato et al. 2014)	ERP005418	Porifera	Metazoa	Holozoa	Opisthokonta	Amorphea
Salpingoeca rosetta	Sros	Ensembl Protist 27	SRP005692		Choanoflagellata	Holozoa	Opisthokonta	Amorphea
Capsaspora owczarzaki	Cowc	(Suga et al. 2013)	SRP022579 / Sebé-Pedrós et al. 2013		Filasterea	Holozoa	Opisthokonta	Amorphea
Creolimax fragrantissima	Cfra	(de Mendoza et al. 2015)	SRP058061 / de Mendoza et al. 2015		Ichthyosporea (Ichthyophonida)	Holozoa	Opisthokonta	Amorphea
Sphaeroforma arctica	Sar3	Broad Institute Multicellularity Initiative	**This study – PRJEB23831**		Ichthyosporea (Ichthyophonida)	Holozoa	Opisthokonta	Amorphea
Chromosphaera perkinsii	Cper	(Grau-Bové et al. 2017)	SRP097609		Ichthyosporea (Dermocystida)	Holozoa	Opisthokonta	Amorphea
Neurospora crassa	Ncrx	Ensembl Fungi 27	SRP016065		Fungi	Holomycota	Opisthokonta	Amorphea
Schizosaccharomyces pombe	Spom	Ensembl Fungi 27	ERP001483		Fungi	Holomycota	Opisthokonta	Amorphea
Aspergillus oryzae	Aory	Ensembl Fungi 27	SRP016952		Fungi	Holomycota	Opisthokonta	Amorphea
Cryptococcus neoformans	Cneo	Broad Institute Fungi Initiative	SRR847297		Fungi	Holomycota	Opisthokonta	Amorphea
Ustilago maydis	Umay	Ensembl Fungi 27	ERP001905		Fungi	Holomycota	Opisthokonta	Amorphea
Tuber melanosporum	Tmel	Ensembl Fungi 27	SRP028655		Fungi	Holomycota	Opisthokonta	Amorphea
Rhizopus oryzae	Rory	Broad Institute Fungi Initiative	SRP031602		Fungi	Holomycota	Opisthokonta	Amorphea
Allomyces macrogynus	Amac	Broad Institute Fungi Initiative	SRP022576		Fungi	Holomycota	Opisthokonta	Amorphea
Spizellomyces punctatus	Spun	Broad Institute Multicellularity Initiative	SRR343043		Fungi	Holomycota	Opisthokonta	Amorphea
Rhizophagus irregularis DAOM 181602	Rirr	Ensembl Fungi 27	DRP002784		Fungi	Holomycota	Opisthokonta	Amorphea
Conidiobolus coronatus	Ccor	JGI v1	SRR427173		Fungi	Holomycota	Opisthokonta	Amorphea
Gonapodya prolifera	Gpro	JGI v3	SRR427152		Fungi	Holomycota	Opisthokonta	Amorphea
Fonticula alba	Falb	Broad Institute Multicellularity Initiative	SRP022580		Nuclearida	Holomycota	Opisthokonta	Amorphea
Dictyostelium discoideum AX4	Ddis2	Ensembl Protist 27 / Dictybase	SRP060392		Amoebozoa			Amorphea
Polysphondylium pallidum	Ppal	Ensembl Protist 27	SRP004023		Amoebozoa			Amorphea
Acanthamoeba castellanii	Acas	Ensembl Protist 27	SRP028620		Amoebozoa			Amorphea
Arabidopsis thaliana	Atha	Ensembl Plants 27	SRP052858 / Max Planck MPP 2015 (Full nutrition samples only)		Embryophyta	Viridiplantae		Diaphoratickes
	Atha2		SRP074840 / Hsu 2016					
Vitis vinifera	Vvin	Ensembl Plants 27	SRP065417		Embryophyta	Viridiplantae		Diaphoratickes
Mimulus guttatus	Mgut	JGI GCF_000504015	SRP045683		Embryophyta	Viridiplantae		Diaphoratickes
Oryza sativa	Osat2	Ensembl Plants 27	DRP000315		Embryophyta	Viridiplantae		Diaphoratickes
Physcomitrella patens	Ppat	Ensembl Plants 27	SRP011279		Embryophyta	Viridiplantae		Diaphoratickes
Selaginella moellendorffii	Smoe	Ensembl Plants 27	SRP059539		Embryophyta	Viridiplantae		Diaphoratickes
Klebsormidium netis (formerly *flaccidum*)	Kfla	(Hori et al. 2014)	SRP048567		Charophyta	Viridiplantae		Diaphoratickes
Volvox carteri	Vcar	JGI 317_v2	SRP066714		Chlorophyta	Viridiplantae		Diaphoratickes
Chlorella variabilis	Cvar	JGI NC64A - 2014-08-18	**NOT ANALYSED**		Chlorophyta	Viridiplantae		Diaphoratickes
Chlamydomonas reinhardtii	Crei	Ensembl Plants 27	ERP001997		Chlorophyta	Viridiplantae		Diaphoratickes
Micromonas pusilla	Mpus	JGI 20110615	SRR847305		Chlorophyta	Viridiplantae		Diaphoratickes
Cyanophora paradoxa	Cpar	Assembly (Price et al. 2012) annotation in-home	SRR363339		Glaucophyta	Viridiplantae		Diaphoratickes
Ectocarpus siliculosus	Esil	(Cock et al. 2010)	SRP037532		Stramenopile	SAR+H		Diaphoratickes
Phytophthora infestans	Pinf	Ensembl Protist 27	SRR1640225		Stramenopile	SAR+H		Diaphoratickes
Aureococcus anophagefferens	Aano	Ensembl Protist 27	SRP045642		Stramenopile	SAR+H		Diaphoratickes
Plasmodium falciparum 3D7	Pfal2	Ensembl Protist 27	SRP069075		Alveolata	SAR+H		Diaphoratickes
Tetrahymena thermophila	Tthe	Ensembl Protist 27	SRP016619		Alveolata	SAR+H		Diaphoratickes
Bigelowiella natans CCMP2755	BnatMEA	Ensembl Protist 27	MMETSP / SRP042159 (Bnat samples only)		Rhizaria	SAR+H		Diaphoratickes
	Bnao		SeqBioproject PRJNA47111 / Curtis et al 2012					
	Bnat		DRP003230 / Suzuki 2016					
Emiliania huxleyi	Ehux	Ensembl Protist 27	SRR847300		Haptophyta	SAR+H		Diaphoratickes
Guillardia theta	Gthe	Ensembl Protist 27	SRR747855		Cryptophyta			Diaphoratickes
Naegleria gruberi	Ngru	Ensembl Protist 27	**This study – PRJEB23822**		Excavata			*see note*

Note: Unikonta is a deprecated name that included all amorphean species here included
Note: Bikonta is a deprecated name that included all Diaphoratiches species here included EXCEPT for the Excavate *Naegleria gruberi*
Species with replicates in blue

Fig S1. Summary of the eukaryotic species used in this study, including its a 4-letter abbreviation, the source of genome assemblies, GFF annotations, and RNA-seq experiments. Dataset accession numbers from SRA unless otherwise stated.

Sps	introns_per_kbpCDS	introns_per_gene	GCC	nu_gene	av_gene	sd_gene	q1_gene	q2_gene	q3_gene	nu_transcript	av_transcript
Hsap	4.86	8.40	0.40	20,346.00	49,576.62	108,719.49	4,364.00	16,412.50	48,309.25	20,346.00	1,680.98
Mmus	4.74	7.71	0.40	22,533.00	36,812.54	96,639.07	2,434.00	10,885.00	32,627.00	22,533.00	1,567.07
Mdom	4.63	7.74	0.40	21,327.00	51,246.38	100,271.47	2,352.50	15,435.00	53,702.50	21,327.00	1,577.76
Oana	5.70	6.58	0.46	21,698.00	20,127.15	41,448.09	2,095.25	6,956.50	18,924.50	21,698.00	1,133.76
Ggal	5.74	9.45	0.42	15,508.00	25,222.35	48,351.56	3,018.75	9,050.00	25,064.25	15,508.00	1,657.19
Xtro	5.63	9.38	0.40	18,442.00	20,546.01	31,989.99	3,616.50	10,262.00	23,813.00	18,442.00	1,618.08
Drex	5.25	8.20	0.36	26,459.00	24,095.14	42,961.53	3,835.00	10,421.00	25,126.00	26,459.00	1,593.47
Cint	5.13	5.37	0.36	16,671.00	3,458.44	4,448.11	975.00	2,113.00	4,312.00	16,671.00	959.30
Bflo	4.06	6.01	0.41	50,817.00	9,116.95	12,701.34	1,900.00	4,932.00	10,995.00	50,817.00	1,424.26
Brabel	4.78	7.71	0.41	30,392.00	8,779.18	12,075.68	1,958.00	4,776.50	10,638.00	30,392.00	1,559.53
Spur2	3.51	4.71	0.37	28,842.00	10,876.96	19,314.30	2,041.25	5,664.00	12,547.50	28,842.00	1,415.35
Dmel	2.00	2.91	0.41	13,917.00	4,481.42	19,384.04	794.00	1,541.00	3,163.00	13,917.00	1,603.38
Cele	4.49	5.07	0.36	20,447.00	2,787.55	3,492.21	1,040.00	1,785.00	3,188.50	20,447.00	1,223.94
Cgig	4.46	6.16	0.34	26,089.00	8,068.40	9,281.77	1,700.00	5,047.00	11,114.00	26,089.00	1,334.76
Ctel	3.73	4.33	0.40	32,175.00	2,631.28	3,220.57	812.00	1,632.00	3,326.50	32,175.00	1,060.68
Sman	3.85	5.34	0.35	10,772.00	14,644.48	19,071.54	1,505.00	7,870.50	20,153.00	10,772.00	1,430.76
Tadh	5.48	7.41	0.33	11,520.00	3,460.18	3,349.96	1,388.00	2,627.00	4,469.00	11,520.00	1,350.78
Hmag	3.61	5.17	0.28	20,047.00	15,036.94	18,415.39	1,457.00	7,656.00	22,497.50	20,047.00	1,406.59
Nvex	3.77	4.31	0.41	24,773.00	4,444.29	6,046.06	734.00	2,300.00	5,872.00	24,773.00	998.89
Aipt	3.80	5.02	0.36	29,271.00	5,548.84	6,546.66	1,644.50	3,463.00	6,960.50	29,271.00	1,543.98
Mlei	3.26	4.46	0.39	16,058.00	5,381.68	7,110.61	1,058.25	2,480.50	6,857.00	16,058.00	1,377.51
Aque	3.60	4.05	0.36	40,122.00	2,394.84	4,478.39	477.00	1,050.00	2,441.00	40,122.00	1,054.25
Ocar	4.41	5.61	0.43	11,152.00	1,963.70	2,056.62	648.00	1,294.00	2,509.75	11,152.00	1,240.86
Scil	2.76	4.22	0.47	26,105.00	6,459.26	9,220.71	635.00	2,596.00	8,744.00	26,105.00	1,274.78
Sros	4.73	7.44	0.56	11,624.00	3,872.65	3,467.72	1,653.75	2,876.50	4,909.00	11,624.00	1,987.00
Cowc	2.41	3.47	0.54	8,741.00	2,457.36	1,841.11	1,276.00	2,042.00	3,138.00	8,741.00	1,951.30
Cfra	4.49	6.58	0.40	8,694.00	3,581.92	3,335.94	1,487.25	2,614.00	4,552.75	8,694.00	1,570.28
Sar3	3.22	4.86	0.38	16,015.00	4,258.88	5,879.84	894.50	2,075.00	5,222.00	16,015.00	1,469.33
Nk52	0.59	0.63	0.43	12,463.00	1,724.22	1,682.00	731.50	1,322.00	2,103.50	12,463.00	1,626.03
Ncrx	2.10	1.74	0.48	9,820.00	1,694.61	1,242.95	895.50	1,422.50	2,142.25	9,820.00	1,456.12
Spom	1.20	0.98	0.36	5,144.00	1,474.80	1,113.16	755.00	1,216.00	1,843.25	5,144.00	1,392.25
Aory	1.83	1.93	0.48	12,074.00	1,574.64	1,143.95	830.00	1,301.50	1,920.75	12,074.00	1,343.44
Cneo	4.18	5.25	0.48	6,962.00	1,901.06	1,265.25	1,039.25	1,613.00	2,392.75	6,962.00	1,562.49
Umay	0.76	0.75	0.54	6,522.00	1,931.39	1,386.88	986.00	1,574.00	2,473.00	6,522.00	1,835.27
Tmel	2.84	2.87	0.45	7,496.00	1,622.05	1,608.21	746.75	1,301.00	2,065.25	7,496.00	1,310.49
Rory	3.01	2.32	0.36	17,459.00	1,211.60	1,068.55	527.00	938.00	1,511.00	17,459.00	1,025.73
Amac	2.07	2.33	0.62	18,773.00	1,680.50	1,296.14	830.00	1,383.00	2,121.00	18,773.00	1,436.38
Spun	3.50	4.81	0.47	8,952.00	1,888.89	1,533.10	942.75	1,484.50	2,324.00	8,952.00	1,546.91
Rirr	3.60	2.42	0.28	29,822.00	1,102.09	970.51	452.00	852.00	1,430.00	29,822.00	804.11
Ccor	1.97	1.74	0.29	10,635.00	1,192.77	954.93	584.50	973.00	1,481.00	10,635.00	1,041.10
Gpro	3.88	4.44	0.52	13,902.00	1,661.10	1,283.09	806.00	1,389.00	2,138.00	13,902.00	1,237.84
Falb	2.54	3.73	0.67	5,881.00	2,940.61	2,704.83	1,209.00	2,143.00	3,786.00	5,881.00	2,046.98
Ddis2	1.44	1.27	0.23	13,212.00	1,767.46	1,588.84	740.75	1,352.00	2,288.25	13,212.00	1,590.51
Ppal	2.51	2.70	0.32	12,367.00	2,046.08	1,750.07	925.00	1,590.00	2,596.00	12,367.00	1,546.27
Acas	4.69	6.06	0.58	14,973.00	1,966.90	1,648.05	846.00	1,526.00	2,534.00	14,973.00	1,308.52
Atha	3.35	4.12	0.35	27,416.00	1,865.96	1,513.37	895.00	1,555.00	2,400.00	27,416.00	1,210.28
Vvin	4.01	3.75	0.35	29,927.00	4,723.53	7,576.34	905.00	2,348.00	5,417.50	29,927.00	1,088.08
Mgut	2.51	4.25	0.36	27,232.00	3,129.65	2,978.29	1,501.00	2,443.00	3,802.50	27,232.00	1,658.85
Osat2	2.53	2.65	0.44	35,679.00	2,160.80	2,307.28	584.00	1,460.00	2,973.00	35,679.00	984.92
Ppat	3.33	3.64	0.33	32,273.00	2,029.13	2,190.49	491.00	1,403.00	2,775.00	32,273.00	1,051.97
Marpol	2.89	3.79	0.43	19,287.00	2,531.25	2,979.02	637.00	1,597.00	3,366.00	19,287.00	1,210.91
Smoe	4.08	4.67	0.46	34,799.00	1,639.01	1,523.35	829.00	1,335.00	2,008.00	34,799.00	1,174.36
Kfla	3.84	5.57	0.52	16,544.00	4,316.25	3,678.23	1,745.00	3,453.00	5,762.25	16,544.00	1,579.69
Vcar	3.88	6.72	0.56	14,247.00	4,758.95	4,613.33	1,614.00	3,557.00	6,324.50	14,247.00	1,954.42
Crei	5.69	7.26	0.64	14,416.00	4,073.64	4,786.97	1,318.00	2,710.00	4,992.50	14,416.00	1,353.75
Mpus	0.93	1.11	0.68	9,886.00	1,844.30	1,671.91	941.00	1,440.00	2,223.00	9,886.00	1,644.98
Cpar	6.31	5.50	0.64	11,011.00	1,505.13	2,062.16	506.00	899.00	1,679.00	11,011.00	902.62
Gthe	4.97	5.32	0.53	24,945.00	1,790.74	1,861.73	707.00	1,275.00	2,240.00	24,945.00	1,219.91
Esil	3.92	6.95	0.53	16,271.00	6,835.59	5,893.37	2,986.00	5,394.00	8,990.50	16,271.00	1,919.92
Pinf	2.00	1.76	0.52	17,785.00	1,477.43	1,385.64	680.00	1,126.00	1,798.00	17,785.00	1,257.98
Aano	0.80	1.28	0.72	11,520.00	2,098.56	2,865.41	662.00	1,172.00	2,375.25	11,520.00	1,567.13
Pfal2	1.56	1.57	0.20	5,349.00	2,555.58	2,668.21	1,021.00	1,691.00	3,071.00	5,349.00	2,291.89
Tthe	2.24	3.61	0.23	24,725.00	2,530.34	2,528.97	905.00	1,796.00	3,329.00	24,725.00	1,941.92
BnatMEA	6.41	7.70	0.45	21,706.00	2,729.27	2,601.02	1,077.00	1,932.00	3,517.00	21,706.00	1,316.16
Ehux	2.58	2.49	0.66	38,544.00	1,604.78	1,701.64	665.00	1,132.00	2,003.00	38,544.00	1,025.53
Ngru	0.53	0.68	0.33	15,709.00	1,639.98	1,596.23	788.00	1,232.00	1,937.00	15,709.00	1,501.18

Sps	sd_transcript	q1_transcript	q2_transcript	q3_transcript	nu_exon	av_exon	sd_exon	q1_exon	q2_exon	q3_exon	nu_intron
Hsap	1,808.68	760.00	1,253.00	2,021.75	198,066.00	172.68	292.53	86.00	123.00	171.00	170,876.00
Mmus	1,657.04	708.00	1,129.00	1,896.00	198,777.00	177.64	274.62	86.00	125.00	173.00	173,785.00
Mdom	1,507.03	777.00	1,158.00	1,895.00	187,581.00	179.38	264.22	86.00	124.00	174.00	164,984.00
Oana	1,202.28	441.00	815.00	1,374.00	165,174.00	148.94	193.51	75.00	117.00	166.00	142,860.00
Ggal	1,728.97	734.75	1,222.00	2,022.00	162,527.00	158.13	214.92	84.00	122.00	168.00	146,625.00
Xtro	1,571.47	812.00	1,218.00	1,962.00	191,723.00	155.64	211.76	75.00	117.00	167.00	173,035.00
Drex	1,656.86	752.00	1,180.00	1,912.00	245,365.00	171.83	266.71	83.00	122.00	173.00	217,089.00
Cint	1,036.57	427.50	703.00	1,236.00	107,347.00	148.98	160.87	88.00	127.00	169.00	89,508.00
Bflo	1,517.25	593.00	1,026.00	1,725.00	356,631.00	202.95	294.13	95.00	136.00	194.00	305,567.00
Brabel	1,739.71	607.00	1,096.00	1,863.00	265,004.00	178.85	243.50	89.00	130.00	184.00	234,318.00
Spur2	1,409.84	580.00	1,037.00	1,741.00	186,994.00	218.30	360.91	97.00	141.00	208.00	135,760.00
Dmel	1,845.01	649.00	1,175.00	1,921.00	55,106.00	404.93	568.63	128.00	216.00	476.00	40,544.00
Cele	1,241.04	587.00	985.00	1,448.50	124,639.00	200.79	224.78	98.00	144.00	232.00	103,679.00
Cgig	1,510.57	498.00	948.00	1,657.00	186,890.00	186.33	271.52	87.00	128.00	185.00	160,801.00
Ctel	1,056.28	484.00	806.00	1,284.00	171,418.00	199.09	251.17	89.00	134.00	204.00	139,228.00
Sman	1,520.16	475.00	971.00	1,826.00	68,476.00	225.07	266.29	95.00	159.00	266.00	57,502.00
Tadh	1,273.52	647.00	1,028.00	1,605.00	96,959.00	160.49	286.43	70.00	101.00	153.00	85,416.00
Hmag	1,212.84	704.00	1,092.00	1,696.00	124,041.00	227.33	322.14	80.00	129.00	231.00	103,671.00
Nvex	997.34	431.00	750.00	1,215.00	131,666.00	187.94	246.78	76.00	117.00	198.00	106,828.00
Aipt	1,663.20	638.00	1,102.00	1,876.00	212,739.00	212.44	358.36	72.00	116.00	207.00	146,958.00
Mlei	1,225.39	621.00	1,049.00	1,688.00	88,550.00	249.80	414.55	101.00	138.00	198.00	71,632.00
Aque	1,300.55	352.00	662.00	1,284.00	204,935.00	206.40	353.89	76.00	122.00	206.00	162,577.00
Ocar	1,232.90	506.00	899.00	1,548.00	73,725.00	187.70	347.37	73.00	109.00	176.00	62,573.00
Scil	1,611.41	443.00	914.00	1,590.00	140,348.00	237.11	444.73	80.00	125.00	219.00	110,121.00
Sros	2,149.36	819.00	1,400.00	2,418.00	98,139.00	235.35	552.61	74.00	108.00	178.00	86,511.00
Cowc	1,755.08	871.00	1,493.00	2,492.00	42,674.00	399.69	775.58	85.00	156.00	357.00	30,361.00
Cfra	1,365.71	744.00	1,218.50	1,935.00	65,960.00	206.97	300.77	94.00	134.00	195.00	57,245.00
Sar3	1,409.67	566.00	1,068.00	1,909.00	93,926.00	250.53	452.04	88.00	138.00	220.00	77,911.00
Nk52	1,627.01	656.00	1,226.00	2,006.00	20,267.00	999.91	1,324.24	205.00	537.00	1,352.00	7,804.00
Ncrx	1,198.03	664.00	1,208.50	1,868.25	26,926.00	531.05	746.81	91.00	239.00	691.00	17,104.00
Spom	1,114.38	667.75	1,133.00	1,750.00	10,210.00	701.44	963.11	105.00	306.00	986.00	5,060.00
Aory	1,039.10	709.00	1,112.00	1,633.75	35,404.00	458.16	633.20	103.00	249.00	574.00	23,330.00
Cneo	1,143.37	794.00	1,299.00	1,991.00	43,483.00	250.17	325.55	78.00	144.00	288.00	36,518.00
Umay	1,362.25	890.00	1,502.00	2,378.00	11,420.00	1,048.13	1,239.99	149.00	567.00	1,574.00	4,896.00
Tmel	1,096.05	578.00	1,074.00	1,728.00	29,021.00	338.49	450.74	87.00	190.00	416.00	21,525.00
Rory	981.54	403.00	769.00	1,288.50	57,974.00	308.90	448.91	79.00	165.00	358.00	40,515.00
Amac	1,178.66	657.00	1,155.00	1,834.00	62,549.00	431.11	595.66	107.00	233.00	516.00	43,771.00
Spun	1,328.12	756.00	1,206.00	1,890.25	51,990.00	266.36	406.27	85.00	144.00	277.00	43,038.00
Rirr	750.59	329.00	576.00	1,051.00	102,015.00	235.06	312.85	72.00	139.00	267.00	72,189.00
Ccor	818.30	509.00	860.00	1,334.00	29,185.00	379.38	537.61	87.00	168.00	446.00	18,543.00
Gpro	1,011.09	588.00	996.00	1,562.00	75,719.00	227.27	281.21	80.00	143.00	263.00	61,776.00
Falb	1,952.83	777.00	1,457.00	2,643.00	27,818.00	432.75	662.76	113.00	215.00	470.75	21,937.00
Ddis2	1,536.54	590.00	1,169.50	2,086.00	30,000.00	700.46	1,035.36	143.00	320.00	826.00	16,764.00
Ppal	1,439.19	615.00	1,183.00	1,952.00	45,714.00	418.31	607.08	93.00	219.00	484.00	33,347.00
Acas	1,099.78	593.00	1,014.00	1,642.00	105,758.00	185.26	225.24	87.00	126.00	195.00	90,702.00
Atha	908.36	587.00	1,040.00	1,559.00	140,573.00	236.04	319.83	83.00	132.00	245.00	112,932.00
Vvin	1,012.89	404.00	869.00	1,468.00	142,121.00	229.12	374.72	78.00	127.00	232.00	112,194.00
Mgut	1,012.40	975.00	1,444.00	2,059.00	143,472.00	314.86	424.34	92.00	161.00	362.00	115,718.00
Osat2	732.87	423.00	797.00	1,348.00	134,690.00	260.90	353.31	84.00	140.00	276.00	94,484.00
Ppat	1,050.31	316.00	746.00	1,440.00	149,804.00	226.63	297.01	83.00	138.00	245.00	117,483.00
Marpol	1,171.57	449.00	912.00	1,556.00	92,597.00	252.22	376.18	86.00	137.00	254.00	73,166.00
Smoe	909.65	591.00	979.00	1,477.00	197,233.00	207.20	276.50	80.00	125.00	215.00	162,417.00
Kfla	1,436.61	695.00	1,196.00	1,956.00	108,752.00	240.31	311.52	95.00	153.00	244.00	92,208.00
Vcar	1,946.60	712.00	1,364.00	2,437.00	110,101.00	252.90	391.38	95.00	147.00	246.00	95,712.00
Crei	1,595.46	518.00	924.00	1,587.00	119,091.00	163.87	208.50	73.00	115.00	179.00	104,641.00
Mpus	1,593.65	811.00	1,267.00	1,976.00	20,845.00	780.15	1,107.83	182.00	461.00	1,010.00	10,945.00
Cpar	1,296.77	341.00	557.00	975.50	71,602.00	138.80	192.03	62.00	96.00	153.00	60,591.00
Gthe	1,438.82	522.00	870.00	1,430.00	157,632.00	193.05	424.27	55.00	82.00	153.00	132,662.00
Esil	1,699.83	911.00	1,527.00	2,409.00	129,904.00	240.48	330.61	98.00	142.00	221.00	113,129.00
Pinf	1,273.34	520.00	929.00	1,538.00	49,144.00	455.26	729.39	89.00	209.00	529.00	31,355.00
Aano	1,785.25	611.00	1,033.00	1,820.00	26,321.00	685.89	788.08	197.00	469.00	908.00	14,797.00
Pfal2	2,622.42	789.00	1,382.00	2,815.00	13,766.00	890.55	1,800.02	78.00	167.00	935.75	8,412.00
Tthe	1,975.87	683.00	1,364.00	2,509.00	114,059.00	420.96	585.77	98.00	215.00	493.00	89,334.00
BnatMEA	1,334.15	611.25	1,001.00	1,561.00	188,833.00	151.29	307.85	53.00	77.00	136.00	167,108.00
Ehux	996.32	503.00	811.00	1,250.00	134,733.00	293.38	453.99	80.00	167.00	377.00	96,155.00
Ngru	1,280.15	743.00	1,166.00	1,817.00	26,397.00	893.36	987.70	289.00	624.00	1,178.00	10,688.00

Sps	av_intron	sd_intron	q1_intron	q2_intron	q3_intron	nu_intergenic	av_intergenic	sd_intergenic	q1_intergenic	q2_intergenic	q3_intergenic
Hsap	5,335.75	18,061.04	490.00	1,493.00	3,888.00	20,306.00	96,895.29	375,804.21	5,551.25	20,204.00	65,875.50
Mmus	4,364.75	15,904.11	443.00	1,279.00	3,102.00	22,492.00	81,306.72	254,072.79	6,348.75	18,901.50	54,822.25
Mdom	5,960.23	16,152.71	532.00	1,633.00	4,476.00	21,316.00	117,440.00	280,182.60	10,624.00	35,061.50	104,131.50
Oana	2,758.12	7,807.23	377.00	1,036.00	2,383.00	10,705.00	68,391.55	176,033.30	6,282.00	17,277.00	56,273.00
Ggal	2,411.86	7,494.01	326.00	765.00	1,741.00	14,699.00	41,975.95	117,841.59	3,351.00	9,159.00	29,724.50
Xtro	1,990.37	4,849.28	296.00	813.00	1,789.00	16,201.00	46,457.13	89,661.43	7,474.00	18,212.00	47,081.00
Drex	2,628.71	7,494.20	135.00	963.00	2,528.00	26,001.00	28,438.17	66,672.31	3,946.00	9,279.00	25,814.00
Cint	434.76	619.23	197.00	322.00	480.00	15,938.00	3,042.26	5,159.30	758.00	1,775.00	3,935.50
Bflo	1,275.04	2,744.69	339.00	531.00	1,030.00	48,842.00	8,420.33	14,514.58	1,620.00	3,756.00	8,842.50
Brabel	924.45	1,917.22	285.00	445.00	773.00	29,025.00	5,044.04	9,398.77	1,092.00	2,479.00	5,303.00
Spur2	1,440.88	3,035.49	490.00	738.00	1,269.00	24,633.00	14,304.76	33,152.34	2,001.00	7,566.00	19,446.00
Dmel	805.20	4,569.09	59.00	67.00	237.00	13,905.00	5,247.09	19,594.36	479.00	1,188.00	4,359.00
Cele	289.62	606.38	47.00	62.00	315.00	20,440.00	2,113.95	3,848.75	440.00	990.00	2,465.00
Cgig	1,090.49	1,851.91	208.00	403.00	1,058.00	22,775.00	11,369.98	17,395.14	1,845.00	5,042.00	13,262.00
Ctel	360.66	1,037.20	52.00	60.00	256.00	25,092.00	6,038.06	7,986.87	1,509.00	3,567.00	7,370.00
Sman	2,423.04	3,276.50	646.00	1,576.00	2,901.00	10,353.00	18,611.24	25,216.00	4,817.00	11,536.00	22,934.00
Tadh	281.92	667.75	93.00	133.00	256.00	10,952.00	5,438.46	13,402.13	1,496.00	2,804.00	5,602.25
Hmag	2,610.53	4,110.70	146.00	667.00	3,473.00	11,102.00	13,924.14	15,882.14	2,794.00	9,829.50	19,895.00
Nvex	795.90	1,446.68	231.00	436.00	818.00	20,675.00	7,565.90	10,212.98	1,602.00	4,107.00	9,412.50
Aipt	629.94	1,210.73	135.00	283.00	558.00	25,895.00	3,092.29	7,339.09	875.00	2,269.00	4,820.50
Mlei	803.79	1,548.92	172.00	271.00	707.00	14,367.00	3,327.87	6,798.16	793.00	2,020.00	4,992.00
Aque	248.88	708.53	49.00	67.00	202.00	30,899.00	1,441.49	5,293.45	174.00	501.00	1,967.50
Ocar	126.83	206.72	51.00	55.00	65.00	5,664.00	1,091.41	1,195.15	287.00	713.00	1,467.00
Scil	1,074.71	1,538.08	378.00	642.00	1,201.00	22,679.00	4,655.72	9,653.28	833.00	3,070.00	7,574.00
Sros	251.35	324.28	178.00	222.00	274.00	11,474.00	863.93	1,778.55	322.00	540.00	861.00
Cowc	120.21	272.25	82.00	100.00	132.00	8,663.00	-2,447.03	523,862.65	229.50	568.00	3,952.00
Cfra	303.35	171.91	178.00	267.00	387.00	8,642.00	1,563.37	1,436.29	735.25	1,213.00	2,009.00
Sar3	571.41	858.70	133.00	376.00	743.00	13,347.00	3,332.51	4,411.29	495.00	1,821.00	4,503.50
Nk52	154.81	314.27	85.00	99.00	121.00	9,806.00	1,066.58	1,213.54	277.00	614.00	1,520.00
Ncrx	134.92	123.73	64.00	83.00	152.00	9,699.00	2,007.41	2,817.63	797.00	1,350.00	2,339.00
Spom	80.82	71.34	45.00	54.00	84.00	5,138.00	966.50	1,676.75	395.00	681.00	1,154.00
Aory	117.66	223.23	53.00	61.00	82.00	12,063.00	1,567.04	3,158.09	493.50	939.00	1,870.00
Cneo	62.54	43.80	50.00	54.00	62.00	6,948.00	806.75	1,897.31	283.75	488.00	906.25
Umay	126.00	109.88	62.00	94.00	144.25	6,258.00	994.33	940.89	388.00	713.00	1,270.75
Tmel	106.50	604.47	54.00	62.00	78.00	7,172.00	11,442.06	27,570.89	844.50	2,398.00	7,930.25
Rory	78.10	56.83	50.00	56.00	76.00	17,378.00	1,421.37	1,626.59	468.00	954.00	1,841.75
Amac	102.66	136.91	68.00	74.00	85.00	18,681.00	1,213.23	2,024.45	394.00	687.00	1,326.00
Spun	69.13	31.43	59.00	64.00	70.00	8,921.00	774.28	1,360.02	224.00	416.00	892.00
Rirr	121.09	190.10	70.00	78.00	94.00	9,860.00	1,013.37	1,344.92	293.00	624.50	1,319.25
Ccor	84.43	267.69	48.00	51.00	55.00	9,709.00	2,388.92	2,290.65	748.00	1,820.00	3,318.00
Gpro	93.17	120.58	60.00	67.00	84.00	13,707.00	1,032.69	1,410.17	232.00	513.00	1,270.00
Falb	237.57	576.93	118.00	151.00	199.00	5,738.00	2,286.62	3,714.50	632.00	1,240.00	2,578.00
Ddis2	136.95	141.98	85.00	103.00	137.00	13,175.00	812.69	891.53	296.00	571.00	1,015.00
Ppal	183.36	252.34	71.00	87.00	166.00	12,324.00	615.60	700.30	194.00	388.00	762.00
Acas	106.52	85.88	76.00	92.00	117.00	14,591.00	779.90	1,418.86	178.00	353.00	719.00
Atha	156.37	166.89	84.00	97.00	155.00	27,409.00	2,496.83	7,684.07	606.00	1,198.00	2,468.00
Vvin	967.73	2,111.00	104.00	243.00	864.00	29,894.00	11,496.82	22,809.47	1,918.00	5,051.50	12,793.00
Mgut	331.75	879.23	94.00	128.00	354.00	26,241.00	7,163.48	19,265.64	668.00	2,150.00	6,806.00
Osat2	405.03	749.96	94.00	148.00	467.00	35,667.00	8,281.12	14,888.63	1,390.00	3,970.00	9,706.00
Ppat	266.15	312.08	146.00	207.00	304.00	31,155.00	11,801.98	21,197.57	1,468.00	3,634.00	11,836.50
Marpol	340.63	484.79	156.00	229.00	357.00	18,524.00	8,372.93	11,618.86	2,225.75	4,893.50	10,591.25
Smoe	97.50	430.14	51.00	57.00	71.00	34,379.00	4,189.77	10,736.71	435.00	1,141.00	3,109.00
Kfla	488.99	304.75	299.00	426.00	598.00	14,836.00	1,799.57	2,326.97	869.00	1,363.00	2,096.00
Vcar	412.74	444.12	213.00	342.00	501.00	14,047.00	4,098.34	8,553.36	877.50	1,986.00	4,126.00
Crei	371.14	528.55	164.00	237.00	362.00	13,867.00	3,578.40	5,989.83	832.00	1,641.00	3,636.50
Mpus	177.77	109.23	137.00	176.00	196.00	9,866.00	365.04	530.33	146.00	232.00	396.00
Cpar	107.49	145.01	57.00	66.00	85.00	1,750.00	1,048.13	1,537.57	206.00	472.00	1,193.00
Gthe	105.28	190.60	46.00	52.00	115.00	24,334.00	1,660.32	2,092.68	431.25	1,063.00	2,087.75
Esil	701.00	714.96	355.00	530.00	802.00	15,609.00	4,436.86	7,794.87	861.00	2,112.00	4,805.00
Pinf	122.45	129.61	62.00	72.00	104.00	17,009.00	8,369.31	19,180.75	310.00	944.00	6,990.00
Aano	411.34	1,192.48	65.00	154.00	300.00	10,491.00	2,822.94	4,113.47	598.50	1,560.00	3,459.00
Pfal2	165.29	104.66	111.00	141.00	185.25	5,335.00	1,680.99	1,518.63	765.50	1,269.00	2,112.00
Tthe	160.86	203.44	56.00	84.00	183.00	23,921.00	1,580.55	1,672.47	695.00	1,135.00	1,855.00
BnatMEA	181.51	168.48	91.00	151.00	227.00	21,446.00	1,624.97	1,865.99	658.00	1,073.00	1,824.00
Ehux	230.07	519.72	74.00	122.00	224.00	34,698.00	2,556.97	3,419.54	595.00	1,545.00	3,272.00
Ngru	202.00	950.15	44.00	57.00	117.00	15,114.00	870.50	5,774.38	200.00	396.00	820.00

nu=counts
q1,q2,q3=first, second and third quartiles of length distribution
av=average length
sd=standard deviation

transcript=gene excluding introns (one per gene)

Fig S2. Genome architecture statistics of the eukaryotic species used in this study, including counts and length distributions (quartiles Q1-3, mean and standard deviation) of genes, transcripts' CDS, exons, introns and intergenic regions, and the GC content of the whole genome, exons and introns (in %).

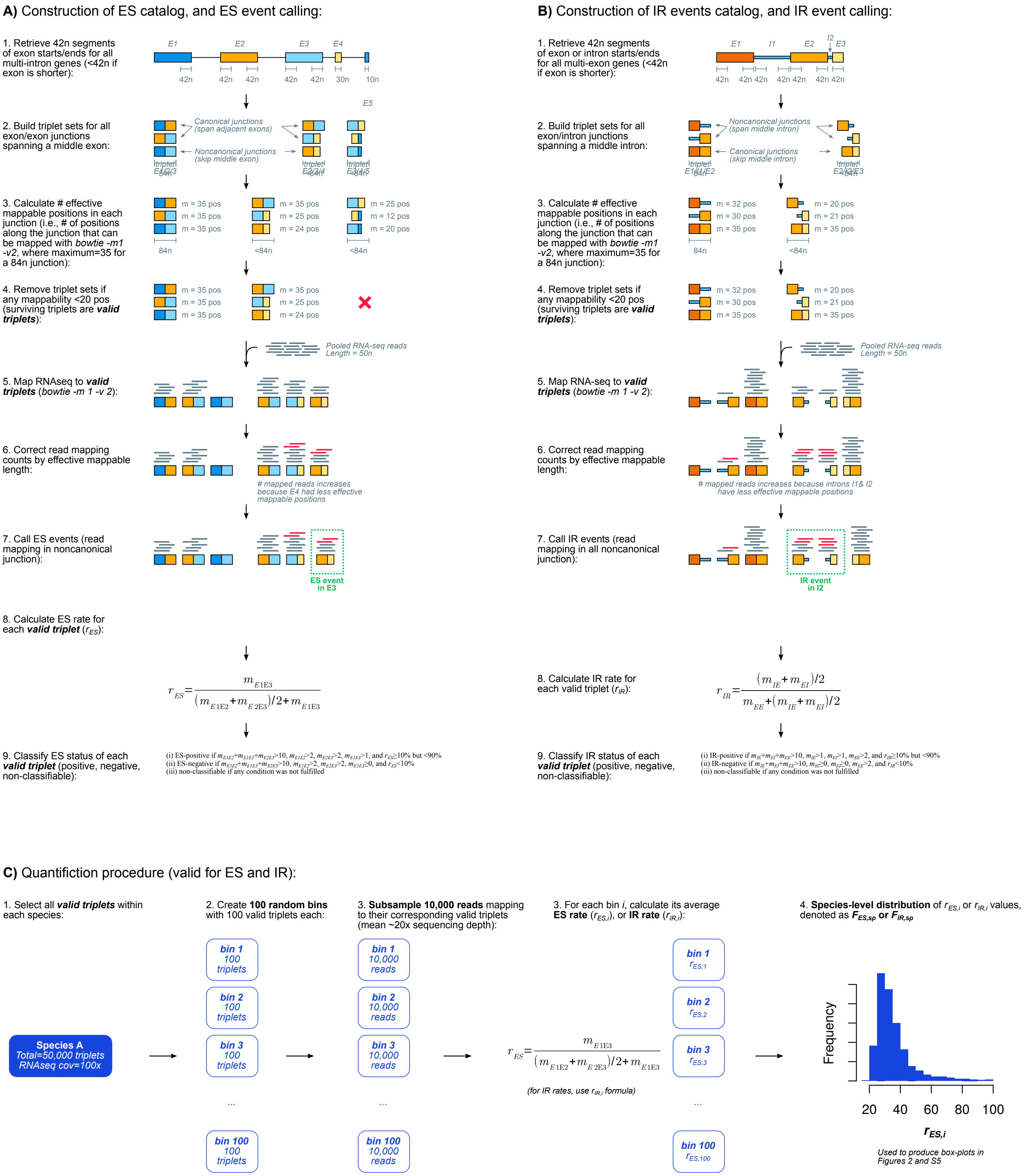

Fig S3. Methods overview.

A-B) Summary of the methodology employed to detect ES (A) and IR events (B), including junction construction, mappability correction and filtering, RNA-seq mapping, AS event calling, and frequency calculations.

C) Quantification procedure followed to analyse ES and IR frequencies at the species level, using valid triplets.

Sps	num total genes	num total introns	num positive genes ES	num positive tripl ES	num negative genes ES	num negative tripl ES	num positive genes IR	num positive tripl IR	num negative genes IR	num negative tripl IR	% positive genes ES	% positive tripl ES	% positive genes IR	% positive tripl IR
Hsap	20,346	177,720	2,144	2,559	10,463	87,405	2,182	4,519	5,480	39,978	10.538	1.440	10.724	2.543
Hsax	20,346	177,720	2,299	2,733	12,390	104,580	5,682	17,646	6,542	37,045	11.300	1.538	27.927	9.929
Mmus	22,533	176,244	1,693	1,978	13,452	121,566	3,321	6,570	7,493	61,161	7.513	1.122	14.738	3.728
Mmux	22,533	176,244	1,167	1,315	11,707	97,783	1,430	2,188	6,852	50,228	5.179	0.746	6.346	1.241
Mdom	21,327	166,254	1,502	1,786	12,062	98,246	1,778	2,974	6,519	50,004	7.043	1.074	8.337	1.789
Oana	21,698	143,476	922	1,015	11,538	64,925	389	456	7,253	37,296	4.249	0.707	1.793	0.318
Ggal	15,508	147,019	1,029	1,195	10,828	90,527	1,923	3,156	5,708	43,245	6.635	0.813	12.400	2.147
Xtro	18,442	173,281	595	652	11,763	83,821	534	636	6,519	47,559	3.226	0.376	2.896	0.367
Xtrx	18,442	173,281	431	471	10,886	72,310	981	1,303	6,255	37,597	2.337	0.272	5.319	0.752
Drex	26,459	218,906	1,370	1,554	17,505	138,745	1,608	2,056	10,223	74,712	5.178	0.710	6.077	0.939
Drer	26,459	218,906	701	774	15,964	124,743	595	712	8,642	64,939	2.649	0.354	2.249	0.325
Cint	16,671	90,676	592	631	6,997	26,701	353	406	3,607	13,358	3.551	0.696	2.117	0.448
Brabel	30,392	234,612	1,279	1,575	16,211	123,136	1,935	2,664	10,087	72,485	4.208	0.671	6.367	1.135
Spur2	28,842	157,677	1,541	1,875	8,863	40,461	246	299	5,490	25,598	5.343	1.189	0.853	0.190
Dmel	13,917	41,189	342	397	6,658	23,632	2,207	4,198	3,805	12,403	2.457	0.964	15.858	10.192
Dmel2	13,917	41,189	350	402	6,593	22,604	616	856	4,305	14,233	2.515	0.976	4.426	2.078
Cele	20,447	104,192	557	616	13,732	63,476	6,517	13,362	6,608	29,035	2.724	0.591	31.873	12.824
Cgig	26,089	160,801	552	657	11,548	79,503	743	993	7,055	46,293	2.116	0.409	2.848	0.618
Ctel	32,175	139,243	425	482	11,147	65,114	1,669	2,193	6,489	35,746	1.321	0.346	5.187	1.575
Sman	10,772	57,702	462	490	5,340	26,280	718	1,036	2,987	14,393	4.289	0.849	6.665	1.795
Tadh	11,520	85,439	20	20	6,613	43,359	402	500	3,494	19,505	0.174	0.023	3.490	0.585
Hmag	20,047	103,994	329	341	9,360	54,471	204	215	5,698	30,965	1.641	0.328	1.018	0.207
Nvec	24,773	106,893	240	250	8,315	49,134	1,288	1,924	4,643	27,336	0.969	0.234	5.199	1.800
Nvex	24,773	106,893	318	335	9,230	53,440	1,229	1,495	5,976	33,802	1.284	0.313	4.961	1.399
Aipt	29,271	183,468	123	124	9,399	54,748	883	1,200	5,273	26,298	0.420	0.068	3.017	0.654
Mlei	16,058	72,492	153	165	7,037	35,668	118	135	3,843	18,998	0.953	0.228	0.735	0.186
Aque	40,122	164,813	101	103	9,914	63,675	1,856	2,827	6,121	32,300	0.252	0.062	4.626	1.715
Ocar	11,152	62,573	12	12	5,571	30,963	790	1,084	3,309	17,622	0.108	0.019	7.084	1.732
Scil	26,105	114,243	85	85	7,771	47,151	523	599	3,839	20,399	0.326	0.074	2.003	0.524
Sros	11,624	86,515	8	8	6,852	38,486	125	127	3,489	15,726	0.069	0.009	1.075	0.147
Cowc	8,741	32,468	9	9	4,357	15,501	592	804	2,250	7,923	0.103	0.028	6.773	2.476
Cfra	8,694	57,266	78	79	6,228	41,670	1,327	2,098	3,282	20,254	0.897	0.138	15.263	3.664
Sar3	16,015	77,911	292	317	6,069	37,679	291	415	3,492	21,533	1.823	0.407	1.817	0.533
Sarc4	33,969	74,290	322	350	7,147	45,274	681	910	4,246	26,269	0.948	0.471	2.005	1.225
Nk52	12,463	7,804	5	5	1,619	2,824	517	721	1,392	2,305	0.040	0.064	4.148	9.239
Ncrx	9,820	17,106	5	5	2,445	3,937	378	425	2,526	4,388	0.051	0.029	3.849	2.485
Spom	5,144	5,066	5	5	1,160	2,230	491	693	845	1,421	0.097	0.099	9.545	13.679
Aory	12,074	23,330	0	0	3,356	6,524	370	461	2,523	5,329	0.000	0.000	3.064	1.976
Cneo	6,962	36,521	6	6	3,694	9,484	394	452	2,225	5,409	0.086	0.016	5.659	1.238
Umay	6,522	4,898	0	0	281	407	193	227	389	530	0.000	0.000	2.959	4.635
Tmel	7,496	21,525	11	11	3,977	10,458	1,013	1,446	2,532	7,114	0.147	0.051	13.514	6.718
Rory	17,459	40,515	4	4	4,865	11,891	826	948	3,468	8,756	0.023	0.010	4.731	2.340
Amac	18,773	43,776	3	3	3,497	5,938	234	245	2,798	4,950	0.016	0.007	1.246	0.560
Spun	8,952	43,038	3	3	4,605	17,308	446	537	2,400	7,261	0.034	0.007	4.982	1.248
Rirr	29,822	72,193	49	49	5,627	21,892	715	908	3,411	12,121	0.164	0.068	2.398	1.258
Rirr2	29,822	72,193	4	4	1,765	4,188	38	41	703	1,506	0.013	0.006	0.127	0.057
Ccor	10,635	18,544	1	1	2,401	6,634	34	37	1,519	3,950	0.009	0.005	0.320	0.200
Gpro	13,902	61,811	4	5	7,501	30,953	411	484	4,259	17,948	0.029	0.008	2.956	0.783
Falb	5,881	21,937	0	0	3,270	9,950	55	57	1,963	5,445	0.000	0.000	0.935	0.260
Ddis2	13,212	16,788	5	5	2,496	3,985	32	32	2,580	4,234	0.038	0.030	0.242	0.191
Ppal	12,367	33,347	0	0	3,651	7,086	86	96	2,871	5,931	0.000	0.000	0.695	0.288
Acas	14,973	90,785	5	5	8,007	40,958	22	23	4,546	24,761	0.033	0.006	0.147	0.025
Atha	27,416	113,157	118	120	13,995	76,060	2,567	3,590	8,243	43,766	0.430	0.106	9.363	3.173
Atha3	27,416	113,157	88	91	10,294	48,586	732	848	5,855	24,626	0.321	0.080	2.670	0.749
Vvin	29,927	112,194	338	351	10,345	56,299	1,529	2,223	5,877	30,872	1.129	0.313	5.109	1.981
Mgut	27,232	116,240	146	150	12,184	67,903	905	1,089	7,310	39,298	0.536	0.129	3.323	0.937
Osat2	35,679	99,011	70	70	9,147	36,854	367	393	4,902	15,525	0.196	0.071	1.029	0.397
Ppat	32,273	117,531	385	396	10,800	56,093	2,022	2,838	5,697	25,914	1.193	0.337	6.265	2.415
Marpol	19,287	73,310	391	423	7,805	50,721	1,386	2,203	4,564	27,604	2.027	0.577	7.186	3.005
Smoe	34,799	162,434	5	5	803	1,574	288	354	788	1,164	0.014	0.003	0.828	0.218
Kfla	16,544	92,208	72	76	8,992	48,544	967	1,214	5,130	28,534	0.435	0.082	5.845	1.317
Vcar	14,247	95,854	421	445	9,018	52,947	2,905	5,380	4,888	24,212	2.955	0.464	20.390	5.613
Crei	14,416	104,675	48	48	8,764	42,403	764	1,001	4,945	25,836	0.333	0.046	5.300	0.956
Mpus	9,886	10,959	13	13	878	1,549	367	532	578	776	0.131	0.119	3.712	4.854
Cpar	11,011	60,591	3	3	1,411	5,681	42	53	731	2,873	0.027	0.005	0.381	0.087
Gthe	24,945	132,687	14	14	11,695	63,250	2,082	4,495	6,453	34,660	0.056	0.011	8.346	3.388
GtheME	24,945	132,687	42	44	10,088	50,474	624	1,044	5,277	24,479	0.168	0.033	2.502	0.787
Esil	16,271	113,633	210	223	11,955	71,691	1,143	1,547	6,813	41,978	1.291	0.196	7.025	1.361
Pinf	17,785	31,359	6	6	2,563	6,125	604	739	1,835	4,140	0.034	0.019	3.396	2.357
Aano	11,520	14,801	2	2	309	684	286	473	274	393	0.017	0.014	2.483	3.196
Pfal2	5,349	8,417	22	24	1,450	4,796	773	1,131	988	2,479	0.411	0.285	14.451	13.437
Tthe	24,725	89,334	48	50	7,841	28,021	969	1,309	4,746	17,065	0.194	0.056	3.919	1.465
BnatMEA	21,706	167,127	258	291	14,630	96,954	4,874	8,715	7,963	49,635	1.189	0.174	22.455	5.215
Bnat	21,706	167,127	114	119	9,994	50,847	1,246	2,076	4,475	19,884	0.525	0.071	5.740	1.242
Bnao	21,706	167,127	501	727	8,456	56,882	2,451	5,623	3,196	10,859	2.308	0.435	11.292	3.365
Ehux	38,544	96,189	5	5	969	1,959	262	365	581	916	0.013	0.005	0.680	0.379
Ngru	15,709	10,688	3	3	1,332	2,091	113	122	1,537	2,534	0.019	0.028	0.719	1.141

Fig S4. Counts of ES and IR events per species, at the gene and exon/intron level.

sp	Median # reads ES	Median # genes ES	# bins ES	Median # reads IR	Median # genes IR	# bins IR
Hsap	10000.0	99.0	100.0	10000	99.00	100
Hsax	10000.0	99.0	100.0	10000	99	100
Mmus	10000.0	99.0	100.0	10000	99	100
Mmux	10000.0	99.0	100.0	10000	99	100
Mdom	10000.0	100.0	100.0	10000	99	100
Oana	10000.0	99.0	100.0	10000	99	100
Ggal	10000.0	99.0	100.0	10000	99	100
Xtro	10000.0	96.0	100.0	10000	99	100
Xtrx	10000.0	99.0	100.0	10000	99	100
Drex	10000.0	99.0	100.0	10000	99	100
Drer	10000.0	96.5	100.0	10000	99	100
Cint	10000.0	99.0	100.0	10000	99	100
Brabel	10000.0	98.0	100.0	10000	99	100
Spur2	10000.0	99.0	100.0	10000	98	100
Dmel	10000.0	98.0	100.0	10000	98	100
Dmel2	10000.0	99.0	100.0	10000	98	100
Cele	10000.0	98.0	100.0	10000	99	100
Cgig	10000.0	99.0	100.0	10000	99	100
Ctel	10000.0	99.0	100.0	10000	99	100
Sman	10000.0	99.0	100.0	10000	98	100
Tadh	7628.0	99.0	100.0	5629	98	100
Hmag	10000.0	99.0	100.0	10000	99	100
Nvec	10000.0	99.0	100.0	9406.5	98	100
Nvex	10000.0	99.0	100.0	10000	99	100
Aipt	10000.0	99.0	100.0	8778.5	98	100
Mlei	10000.0	99.0	100.0	10000	98	100
Aque	10000.0	99.0	100.0	10000	99	100
Ocar	10000.0	98.5	100.0	10000	97.5	100
Scil	10000.0	99.0	100.0	9346.5	98	100
Sros	6653.5	99.0	100.0	4789.5	98	100
Cowc	10000.0	98.5	100.0	10000	98	87
Cfra	10000.0	99.0	100.0	10000	98	100
Sar3	10000.0	99.0	100.0	10000	98	100
Sarc4	10000.0	99.0	100.0	10000	98	100
Nk52	10000.0	97.5	28.0	10000	98	30
Ncrx	10000.0	96.0	39.0	10000	99	48
Spom	10000.0	94.5	22.0	10000	97	21
Aory	10000.0	98.0	65.0	10000	98	57
Cneo	10000.0	98.5	94.0	10000	97	58
Umay	10000.0	87.0	4.0	10000	96	7
Tmel	10000.0	98.0	100.0	10000	98	85
Rory	10000.0	99.0	100.0	10000	98	97
Amac	6187.0	99.0	59.0	5542	99	51
Spun	7202.0	98.0	100.0	5701	97	77
Rirr	10000.0	99.0	100.0	9521	98	100
Rirr2	3141.0	98.0	41.0	2983	94	15
Ccor	10000.0	98.0	66.0	10000	97	39
Gpro	10000.0	99.0	100.0	10000	99	100
Falb	10000.0	98.0	99.0	7628	97	55
Ddis2	10000.0	97.0	39.0	10000	99	42
Ppal	10000.0	99.0	70.0	10000	99	60
Acas	10000.0	99.0	100.0	10000	99	100
Atha	10000.0	99.0	100.0	10000	99	100
Atha3	10000.0	99.0	100.0	10000	99	100
Vvin	10000.0	99.0	100.0	10000	99	100
Mgut	10000.0	99.0	100.0	10000	99	100
Osat2	8999.0	100.0	100.0	6125	99	100
Ppat	9404.0	99.0	100.0	6799	99	100
Marpol	10000.0	99.0	100.0	10000	98	100
Smoe	10000.0	95.0	15.0	10000	96	15
Kfla	10000.0	99.0	100.0	10000	99	100
Vcar	10000.0	98.0	100.0	10000	99	100
Crei	10000.0	99.0	100.0	10000	99	100
Mpus	10000.0	93.0	15.0	10000	92	13
Cpar	10000.0	94.0	56.0	10000	92	29
Gthe	10000.0	100.0	100.0	10000	99	100
GtheME	10000.0	99.0	100.0	7745	99	100
Esil	10000.0	98.0	100.0	10000	99	100
Pinf	10000.0	98.0	61.0	10000	97	48
Aano	10000.0	83.0	6.0	10000	88.5	8
Pfal2	10000.0	93.0	48.0	10000	95	36
Tthe	10000.0	99.0	100.0	10000	99	100
BnatMEA	10000.0	100.0	100.0	10000	99	100
Bnat	6836.0	99.0	100.0	5268.5	99	100
Bnao	4641.5	99.0	100.0	4324.5	98	100
Ehux	9002.0	95.0	19.0	10000	95	12
Ngru	10000.0	96.0	20.0	10000	98	26

Fig S5. Number of bins and sub-sampled reads used in the quantification of ES and IR for each species.

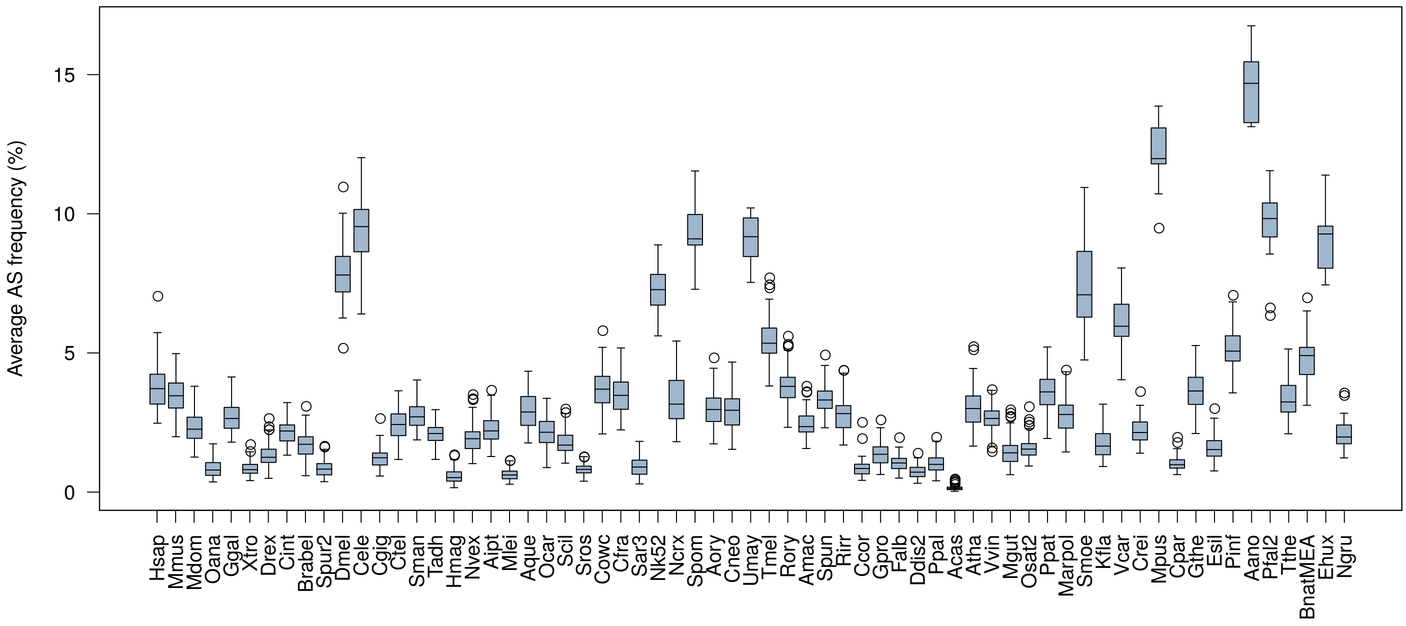

Fig S6. Distribution of IR frequencies across 65 eukaryotes (see Supplementary Figure S1 for complete list of transcriptomic datasets). Each box-plot represents the distribution of IR frequencies (F*IR,sp*) in a given species (as %), drawn from mapping up to 10,000 RNA-seq reads in 100 exon junctions, in up to 100 independent sub-sets (see Methods and Supplementary Figure S3).

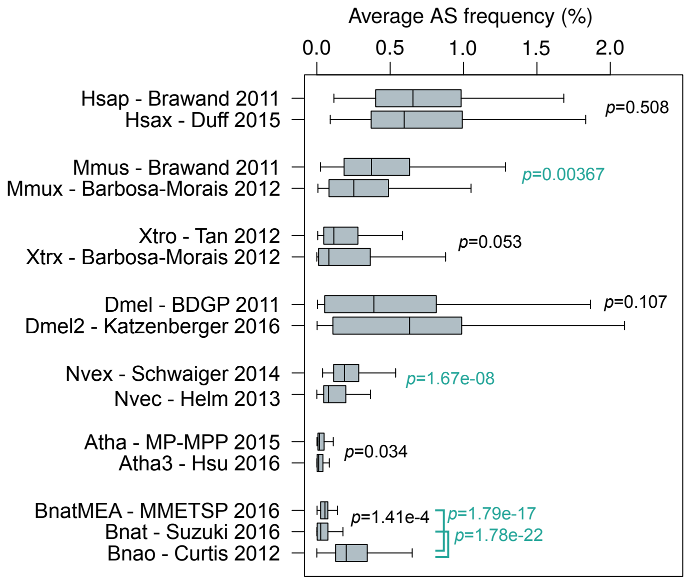

Fig S7. Replicate analyses of ES frequency (*FES,sp*) in a selection of species, using independently-generated RNA-seq datasets (see Supplementary Figure S1 for a complete list of transcriptomic dataset sources). Replicate analyses have been performed for *H. sapiens, M. musculus, X. tropicalis, D. melanogaster, N. vectensis* (animals), *A. thaliana* (plant) and *B. natans* (chlorarachniophyte).

A) ES frequency, per species and tissue

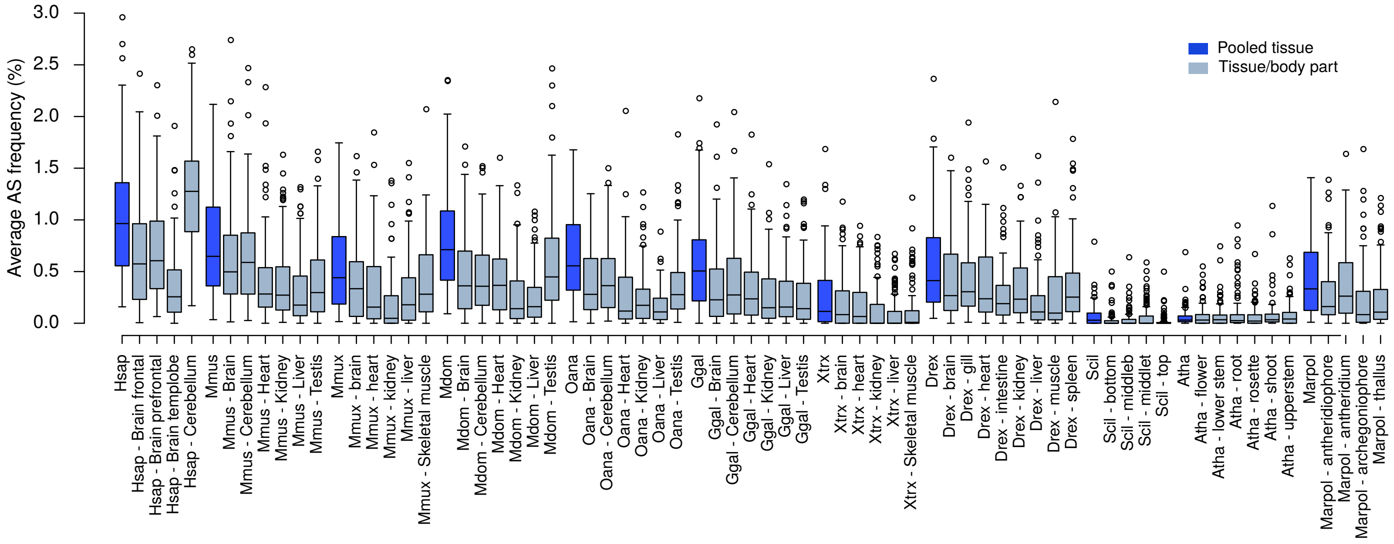

B) ES-positive genes per species and tissue

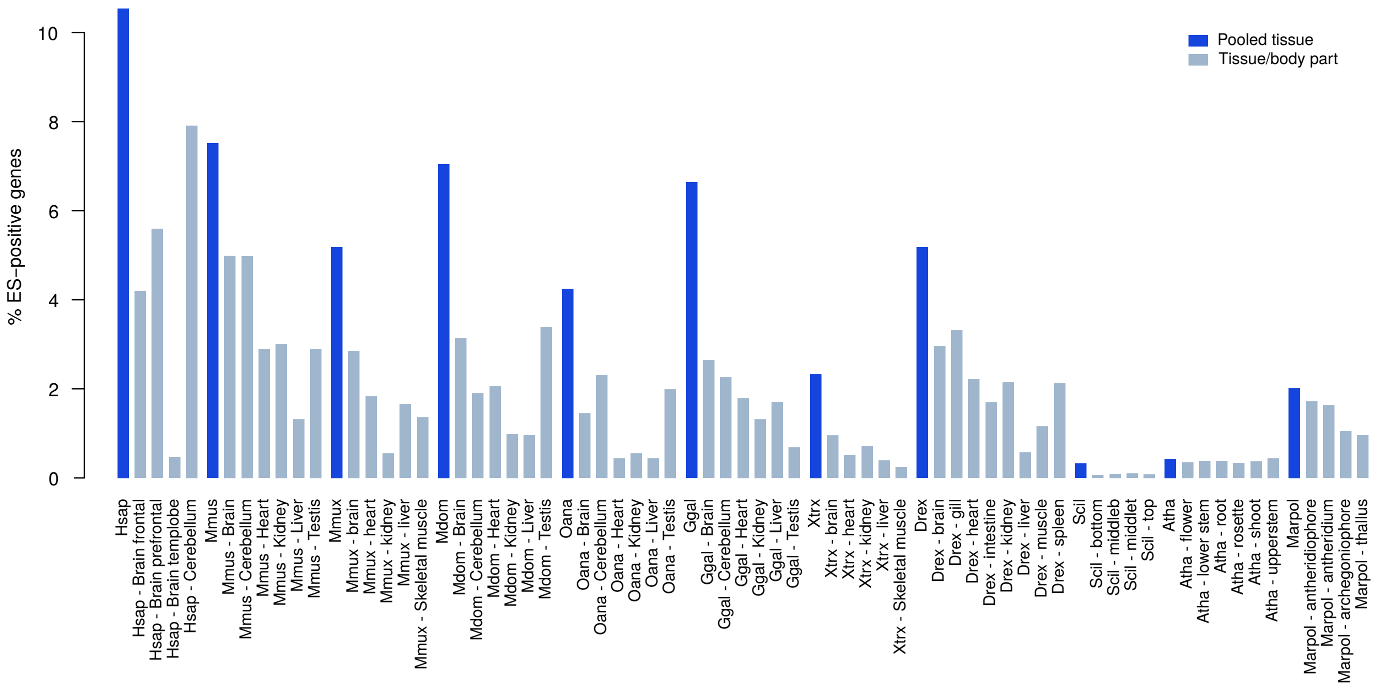

Fig S8. Tissue-level ES quantification in samples from selected animals (*H. sapiens,* 2x *M. musculus, M. domestica, O. anatinus, G. gallus, X. tropicalis, D. rerio, S. ciliatum*) and plants (*A. thaliana* and *M. polymorpha*).

A) Percentage of ES-positive genes per sample, including multi-tissue pool.

B) Distribution of ES frequencies per sample, including multi-tissue pool.

Complement to Fig2 C – Animals only

Group i	Group j	pval	D statistic	Ni	Nj
Verts	Nonvert-bil	1.02E-03	9.61E-02	700	800
Verts	Nonbil	2.73E-114	5.92E-01	700	800
Verts	Unihol	1.56E-95	6.41E-01	700	428
Verts	Uniopi	9.10E-262	8.64E-01	700	948
Nonvert-bil	Verts	1.21E-01	5.32E-02	800	700
Nonvert-bil	Nonbil	5.54E-92	5.13E-01	800	800
Nonvert-bil	Unihol	5.53E-77	5.61E-01	800	428
Nonvert-bil	Uniopi	1.99E-227	7.76E-01	800	948
Nonbil	Verts	1.00E+00	-2.17E-17	800	700
Nonbil	Nonvert-bil	1.00E+00	0.00E+00	800	800
Nonbil	Unihol	4.64E-02	7.42E-02	800	428
Nonbil	Uniopi	6.67E-38	3.14E-01	800	948
Unihol	Verts	1.00E+00	7.37E-17	428	700
Unihol	Nonvert-bil	1.00E+00	9.54E-17	428	800
Unihol	Nonbil	2.40E-02	8.18E-02	428	800
Unihol	Uniopi	9.85E-25	3.06E-01	428	948
Uniopi	Verts	1.00E+00	8.33E-17	948	700
Uniopi	Nonvert-bil	1.00E+00	1.05E-16	948	800
Uniopi	Nonbil	1.00E+00	1.05E-16	948	800
Uniopi	Unihol	1.00E+00	9.54E-18	948	428

Species groupings.

Verts= "Hsap","Mmus","Mdom","Oana","Ggal","Xtro","Drex"
Nonvert-bil= "Cint","Brabel","Spur2","Dmel","Cele","Cgig","Ctel","Sman"
Nonbil= "Tadh","Hmag","Nvec","Aipt","Mlei","Aque","Ocar","Scil"
Unihol= "Sar3","Sros","Cowc","Cfra","Nk52"
Uniopi= "Ncrx","Spom","Aory","Cneo","Umay","Tmel","Rory",
"Amac","Spun","Rirr","Ccor","Gpro","Falb"

Hypothesis:
Alternative hypothesis: the CDF of *i* lies below that of *j*
Ni= num bins in group I
Nj= num bins in group J

Complement to Fig2 D – All holozoans

Group i	Group j	pval	D statistic	Ni	Nj
Bilateria	Cnidaria	6.81E-35	3.97E-01	1500	300
Bilateria	Trichoplax	6.49E-57	8.31E-01	1500	100
Bilateria	Mnemiopsis	9.30E-18	4.57E-01	1500	100
Bilateria	Porifera	1.94E-122	7.49E-01	1500	300
Bilateria	Unicell-Holozoa	1.20E-102	5.94E-01	1500	428
Cnidaria	Bilateria	1.00E+00	-9.76E-17	300	1500
Cnidaria	Trichoplax	3.93E-27	6.37E-01	300	100
Cnidaria	Mnemiopsis	1.55E-02	1.67E-01	300	100
Cnidaria	Porifera	1.07E-28	4.63E-01	300	300
Cnidaria	Unicell-Holozoa	1.15E-16	3.23E-01	300	428
Trichoplax	Bilateria	1.00E+00	-5.42E-17	100	1500
Trichoplax	Cnidaria	1.00E+00	4.34E-17	100	300
Trichoplax	Mnemiopsis	1.00E+00	0.00E+00	100	100
Trichoplax	Porifera	1.00E+00	4.34E-17	100	300
Trichoplax	Unicell-Holozoa	1.00E+00	-9.54E-17	100	428
Mnemiopsis	Bilateria	1.00E+00	-5.42E-17	100	1500
Mnemiopsis	Cnidaria	4.80E-01	7.00E-02	100	300
Mnemiopsis	Trichoplax	4.48E-19	6.50E-01	100	100
Mnemiopsis	Porifera	2.68E-10	3.83E-01	100	300
Mnemiopsis	Unicell-Holozoa	2.06E-04	2.29E-01	100	428
Porifera	Bilateria	1.00E+00	-9.76E-17	300	1500
Porifera	Cnidaria	1.00E+00	0.00E+00	300	300
Porifera	Trichoplax	2.95E-08	3.40E-01	300	100
Porifera	Mnemiopsis	1.00E+00	-4.34E-17	300	100
Porifera	Unicell-Holozoa	1.00E+00	9.97E-04	300	428
Unicell-Holozoa	Bilateria	1.00E+00	4.12E-17	428	1500
Unicell-Holozoa	Cnidaria	1.00E+00	1.39E-16	428	300
Unicell-Holozoa	Trichoplax	2.34E-20	5.28E-01	428	100
Unicell-Holozoa	Mnemiopsis	1.00E+00	9.54E-17	428	100
Unicell-Holozoa	Porifera	1.13E-07	2.13E-01	428	300

Species groupings.

Bilateria= "Hsap","Mmus","Mdom","Oana","Ggal","Xtro","Drex",
"Cint","Brabel","Spur2","Dmel","Cele","Cgig","Ctel","Sman"
Cnidaria-bil= "Hmag","Nvec","Aipt"
Trichoplax= Tadh
Mnemiopsis= Mlei
Porifera= "Aque","Ocar","Scil"
Uc. Holozoa= "Sar3","Sros","Cowc","Cfra","Nk52"

Hypothesis:
Alternative hypothesis: the CDF of *i* lies below that of *j*
Ni= num bins in group I
Nj= num bins in group J

Complement to Fig2 E – Unicell Holozoa

Group i	Group j	pval	D statistic	Ni	Nj
Sros	Cowc	1.00E+00	0.00E+00	100	100
Sros	Sarc	1.00E+00	0.00E+00	100	100
Sros	Cfra	1.00E+00	0.00E+00	100	100
Sros	Cper	1.00E+00	-7.63E-17	100	28
Cowc	Sros	1.78E-26	7.70E-01	100	100
Cowc	Sarc	1.00E+00	0.00E+00	100	100
Cowc	Cfra	5.27E-01	8.00E-02	100	100
Cowc	Cper	9.68E-01	2.71E-02	100	28
Sarc	Sros	2.33E-34	8.80E-01	100	100
Sarc	Cowc	3.78E-27	7.80E-01	100	100
Sarc	Cfra	1.63E-18	6.40E-01	100	100
Sarc	Cper	4.49E-10	7.01E-01	100	28
Cfra	Sros	2.10E-21	6.90E-01	100	100
Cfra	Cowc	6.82E-04	2.70E-01	100	100
Cfra	Sarc	1.00E+00	0.00E+00	100	100
Cfra	Cper	5.49E-01	1.17E-01	100	28
Cper	Sros	6.91E-13	8.00E-01	28	100
Cper	Cowc	3.13E-02	2.81E-01	28	100
Cper	Sarc	9.71E-01	2.57E-02	28	100
Cper	Cfra	5.17E-01	1.23E-01	28	100

Hypothesis:
Alternative hypothesis: the CDF of *i* lies below that of *j*
Ni= num bins in group I
Nj= num bins in group J

Complement to Fig2 F – plants

Group i	Group j	pval	D statistic	Ni	Nj
Plants	V. carteri	9.28E-01	2.09E-02	615	100
Plants	Unicell chlorophytes	4.13E-08	2.31E-01	615	215
Plants	Unicell Bikonta	3.92E-31	3.64E-01	615	462
V. carteri	Plants	9.86E-14	4.17E-01	100	615
V. carteri	Unicell chlorophytes	8.67E-22	5.96E-01	100	215
V. carteri	Unicell Bikonta	3.91E-37	7.14E-01	100	462
Unicell chlorophytes	Plants	1.00E+00	1.30E-17	215	615
Unicell chlorophytes	V. carteri	1.00E+00	3.47E-17	215	100
Unicell chlorophytes	Unicell Bikonta	2.69E-07	2.27E-01	215	462
Unicell Bikonta	Plants	1.00E+00	5.38E-04	462	615
Unicell Bikonta	V. carteri	9.99E-01	2.16E-03	462	100
Unicell Bikonta	Unicell chlorophytes	9.99E-01	2.16E-03	462	215

Species groupings.

Plants= "Atha","Vvin","Mgut","Osat2","Ppat","Marpol","Smoe"
V. carteri-bil= Vcar
Unicell chlorophyta= "Crei","Mpus","Kfla"
Unicell bikonta= "Cpar","Pinf","Aano","Pfal","Tthe","Bnat","Ehux","Gthe","Ngru"

Hypothesis:
Alternative hypothesis: the CDF of *i* lies below that of *j*
Ni= num bins in group I
Nj= num bins in group J

Fig S9. Inter-group comparisons of ES frequencies using one-sided Kolmogorov-Smirnov 2-sample tests. If significant, ES frequencies are higher in species i than j. For each comparison, p-values, D statistic and number of observations are reported.

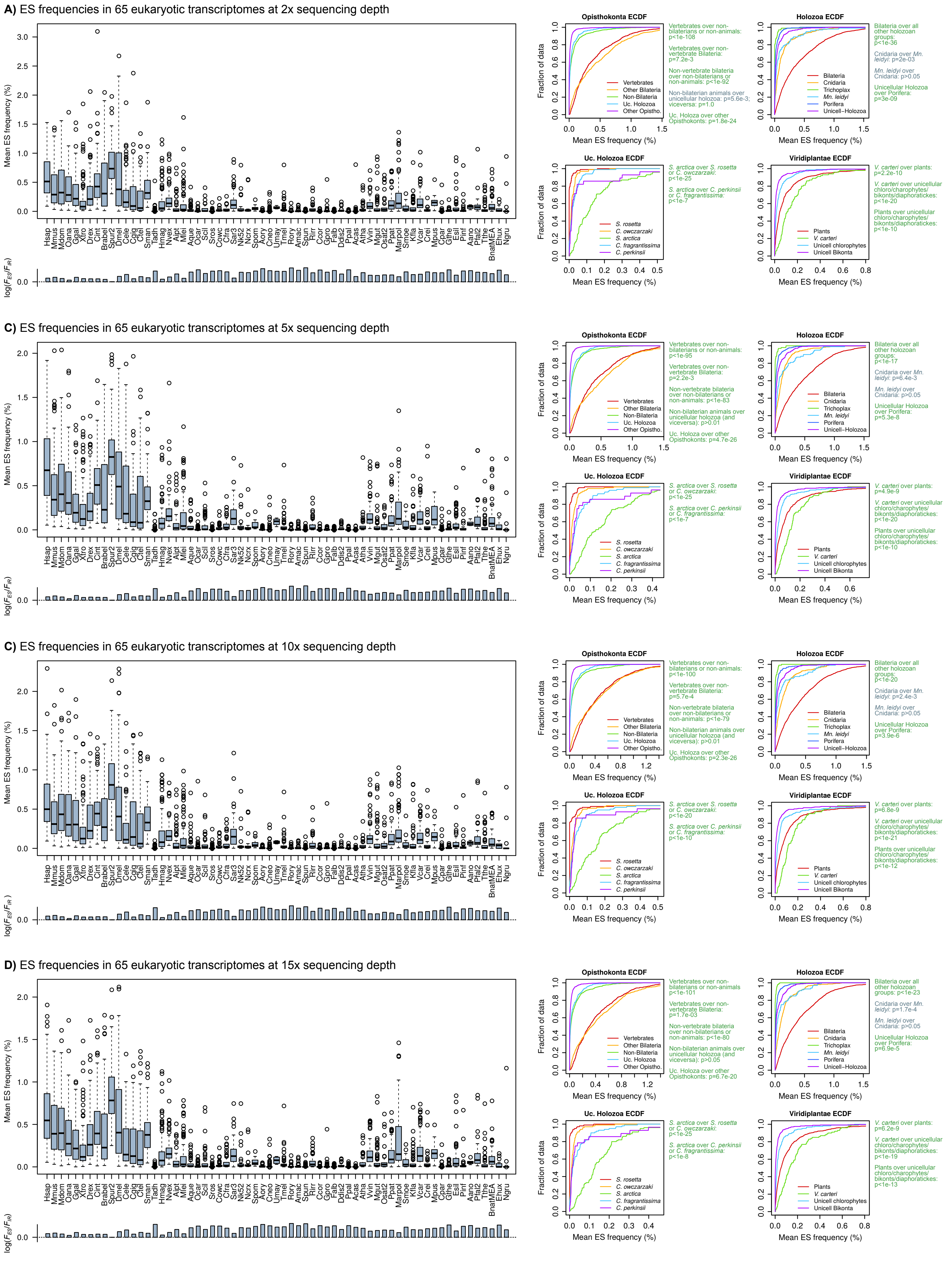

Fig S10. Distribution of ES frequencies across 65 eukaryotic transcriptomes (as in Figure 2), downsampling each RNA-seq sample at 2x (A), 5x (B), 10x (C), and 15x (D).

For each downsampling analysis, we report 1) box-plots representing the distribution of ES frequencies (*FES,sp*) in a given species (as %; upper left panel); 2) log-ratio of mean *FES,sp* (for ES) over mean *FIR,sp* (for IR) in each species (lower left panel); 3) comparisons of ES frequencies among phylogenetically-related eukaryotic groups (right panels) using ECDF plots representing pooled F ES,sp values of multiple species.

Comparison plots also report whether the statistical contrasts described in Figure 2 and S7 (one-sided Kolmogorov-Smirnov 2-sample tests) remain significant (in green) or not (in grey) in each RNA-seq downsampling analysis.

spi	IR-neg 3n-neg	IR-neg 3n-pos	% IR-neg 3n-pos	IR-pos 3n-neg	IR-pos 3n-pos	% IR-pos 3n-pos	Fisher pval	Fisher statistic	Is there any bias? P<0.01	% IR positive
Hsap	26,595	13,383	0.335	3,007	1,512	0.335	9.87E-01	0.999	FALSE	10.724
Mmus	40,705	20,456	0.334	4,413	2,157	0.328	3.22E-01	0.973	FALSE	14.738
Mdom	33,246	16,758	0.335	1,982	992	0.334	8.73E-01	0.993	FALSE	8.337
Oana	24,950	12,346	0.331	296	160	0.351	3.68E-01	1.092	FALSE	1.793
Ggal	28,862	14,383	0.333	2,138	1,018	0.323	2.56E-01	0.955	FALSE	12.400
Xtro	31,706	15,853	0.333	417	219	0.344	5.54E-01	1.050	FALSE	2.896
Drex	49,843	24,869	0.333	1,399	657	0.320	2.09E-01	0.941	FALSE	6.077
Cint	8,870	4,488	0.336	273	133	0.328	7.49E-01	0.963	FALSE	2.117
Brabel	48,424	24,061	0.332	1,780	884	0.332	1.00E+00	0.999	FALSE	6.367
Spur2	17,169	8,429	0.329	202	97	0.324	9.02E-01	0.978	FALSE	0.853
Dmel	8,324	4,079	0.329	2,801	1,397	0.333	6.49E-01	1.018	FALSE	15.858
Cele	19,476	9,559	0.329	8,946	4,416	0.330	7.98E-01	1.006	FALSE	31.873
Cgig	30,939	15,354	0.332	667	326	0.328	8.38E-01	0.985	FALSE	2.848
Ctel	24,212	11,534	0.323	1,489	704	0.321	8.88E-01	0.992	FALSE	5.187
Sman	9,668	4,725	0.328	711	325	0.314	3.55E-01	0.935	FALSE	6.665
Tadh	13,090	6,415	0.329	334	166	0.332	8.85E-01	1.014	FALSE	3.490
Hmag	20,628	10,337	0.334	143	72	0.335	1.00E+00	1.005	FALSE	1.018
Nvex	22,681	11,121	0.329	979	516	0.345	1.96E-01	1.075	FALSE	4.961
Aipt	17,460	8,838	0.336	777	423	0.353	2.48E-01	1.075	FALSE	3.017
Mlei	12,689	6,309	0.332	94	41	0.304	5.22E-01	0.877	FALSE	0.735
Aque	21,605	10,695	0.331	1,933	894	0.316	1.08E-01	0.934	FALSE	4.626
Ocar	11,879	5,743	0.326	731	353	0.326	1.00E+00	0.999	FALSE	7.084
Scil	13,580	6,819	0.334	399	200	0.334	1.00E+00	0.998	FALSE	2.003
Sros	10,602	5,124	0.326	81	46	0.362	3.93E-01	1.175	FALSE	1.075
Cowc	5,347	2,576	0.325	552	252	0.313	5.27E-01	0.948	FALSE	6.773
Cfra	13,520	6,734	0.332	1,411	687	0.327	6.61E-01	0.978	FALSE	15.263
Sar3	14,413	7,120	0.331	286	129	0.311	4.29E-01	0.913	FALSE	1.817
Nk52	1,540	765	0.332	502	219	0.304	1.72E-01	0.878	FALSE	4.148
Ncrx	2,966	1,422	0.324	268	157	0.369	5.85E-02	1.222	FALSE	3.849
Spom	961	460	0.324	451	242	0.349	2.58E-01	1.121	FALSE	9.545
Aory	3,683	1,646	0.309	319	142	0.308	1.00E+00	0.996	FALSE	3.064
Cneo	3,670	1,739	0.322	314	138	0.305	4.96E-01	0.928	FALSE	5.659
Umay	368	162	0.306	168	59	0.260	2.22E-01	0.798	FALSE	2.959
Tmel	4,780	2,334	0.328	987	459	0.317	4.42E-01	0.952	FALSE	13.514
Rory	5,924	2,832	0.323	656	292	0.308	3.42E-01	0.931	FALSE	4.731
Amac	3,361	1,589	0.321	179	66	0.269	9.23E-02	0.780	FALSE	1.246
Spun	4,942	2,319	0.319	376	161	0.300	3.62E-01	0.913	FALSE	4.982
Rirr	8,040	4,081	0.337	606	302	0.333	8.27E-01	0.982	FALSE	2.398
Ccor	2,646	1,304	0.330	27	10	0.270	4.88E-01	0.752	FALSE	0.320
Gpro	12,264	5,684	0.317	328	156	0.322	8.05E-01	1.026	FALSE	2.956
Falb	3,670	1,775	0.326	44	13	0.228	1.54E-01	0.611	FALSE	0.935
Ddis2	2,795	1,439	0.340	22	10	0.313	8.52E-01	0.883	FALSE	0.242
Ppal	3,975	1,956	0.330	73	23	0.240	6.31E-02	0.640	FALSE	0.695
Acas	16,658	8,103	0.327	14	9	0.391	5.11E-01	1.322	FALSE	0.147
Atha	29,349	14,417	0.329	2,395	1,195	0.333	6.71E-01	1.016	FALSE	9.363
Vvin	20,767	10,105	0.327	1,460	763	0.343	1.23E-01	1.074	FALSE	5.109
Mgut	26,074	13,224	0.337	738	351	0.322	3.46E-01	0.938	FALSE	3.323
Osat2	10,286	5,239	0.337	262	131	0.333	9.14E-01	0.982	FALSE	1.029
Ppat	17,338	8,576	0.331	1,864	974	0.343	1.93E-01	1.056	FALSE	6.265
Marpol	18,396	9,208	0.334	1,473	730	0.331	8.51E-01	0.990	FALSE	7.186
Smoe	790	374	0.321	238	116	0.328	8.46E-01	1.030	FALSE	0.828
Kfla	19,087	9,447	0.331	801	413	0.340	5.13E-01	1.042	FALSE	5.845
Vcar	16,235	7,977	0.329	3,642	1,738	0.323	3.69E-01	0.971	FALSE	20.390
Crei	17,232	8,604	0.333	640	361	0.361	7.04E-02	1.130	FALSE	5.300
Mpus	518	258	0.332	375	157	0.295	1.64E-01	0.841	FALSE	3.712
Cpar	1,990	883	0.307	35	18	0.340	6.53E-01	1.159	FALSE	0.381
Gthe	23,553	11,107	0.320	3,038	1,457	0.324	6.22E-01	1.017	FALSE	8.346
Esil	28,134	13,844	0.330	1,052	495	0.320	4.25E-01	0.956	FALSE	7.025
Pinf	2,806	1,334	0.322	494	245	0.332	6.39E-01	1.043	FALSE	3.396
Aano	280	113	0.288	329	144	0.304	6.02E-01	1.084	FALSE	2.483
Pfal2	1,625	854	0.344	762	369	0.326	2.89E-01	0.921	FALSE	14.451
Tthe	12,277	4,788	0.281	939	370	0.283	8.73E-01	1.010	FALSE	3.919
BnatMEA	33,268	16,367	0.330	5,911	2,804	0.322	1.45E-01	0.964	FALSE	22.455
Ehux	613	303	0.331	240	125	0.342	6.94E-01	1.054	FALSE	0.680
Ngru	1,752	782	0.309	75	47	0.385	8.85E-02	1.404	FALSE	0.719

Fig S11. Enrichment in 3n-divisbility of intron lengths in IR events from whole transcriptomes (‘all’), with Fisher’s exact test.

A) Whole transcriptome

spi	ES-neg 3n-neg	ES-neg 3n-pos	% ES-neg 3n-pos	ES-pos 3n-neg	ES-pos 3n-pos	% ES-pos 3n-pos	Fisher pval	Fisher statistic	Is there any bias? P<0.01	ES-positive %
Hsap	52,747	34,658	0.397	1,182	1,377	0.538	5.83E-46	1.773	TRUE	10.538
Mmus	73,063	48,503	0.399	650	1,328	0.671	1.09E-129	3.078	TRUE	7.513
Mdom	59,150	39,096	0.398	558	1,228	0.688	2.15E-132	3.330	TRUE	7.043
Oana	38,757	26,168	0.403	352	663	0.653	4.64E-57	2.790	TRUE	4.249
Ggal	54,658	35,869	0.396	427	768	0.643	4.75E-65	2.741	TRUE	6.635
Xtro	50,021	33,800	0.403	198	454	0.696	4.65E-51	3.393	TRUE	3.226
Drex	82,685	56,060	0.404	400	1,154	0.743	1.60E-159	4.255	TRUE	5.178
Cint	16,557	10,144	0.380	386	245	0.388	6.78E-01	1.036	FALSE	3.551
Brabel	72,694	50,442	0.410	280	1,295	0.822	5.81E-245	6.666	TRUE	4.208
Spur2	23,998	16,463	0.407	1,134	741	0.395	3.24E-01	0.953	FALSE	5.343
Dmel	14,973	8,659	0.366	62	335	0.844	3.41E-84	9.340	TRUE	2.457
Cele	38,663	24,813	0.391	191	425	0.690	3.67E-50	3.467	TRUE	2.724
Cgig	47,042	32,461	0.408	161	496	0.755	5.31E-72	4.464	TRUE	2.116
Ctel	39,205	25,909	0.398	116	366	0.759	7.66E-58	4.774	TRUE	1.321
Sman	15,931	10,349	0.394	303	187	0.382	6.08E-01	0.950	FALSE	4.289
Tadh	25,836	17,523	0.404	8	12	0.600	1.08E-01	2.212	FALSE	0.174
Hmag	32,045	22,426	0.412	181	160	0.469	3.58E-02	1.263	FALSE	1.641
Nvex	31,930	21,510	0.403	166	169	0.504	1.76E-04	1.511	TRUE	1.284
Aipt	31,841	22,907	0.418	47	77	0.621	6.41E-06	2.277	TRUE	0.420
Mlei	22,173	13,495	0.378	38	127	0.770	2.81E-24	5.491	TRUE	0.953
Aque	36,229	27,446	0.431	56	47	0.456	6.20E-01	1.108	FALSE	0.252
Ocar	17,558	13,405	0.433	7	5	0.417	1.00E+00	0.936	FALSE	0.108
Scil	26,985	20,166	0.428	44	41	0.482	3.25E-01	1.247	FALSE	0.326
Sros	NA	NA	NA	NA	NA	NA	NA	NA	NA	0.069
Cowc	NA	NA	NA	NA	NA	NA	NA	NA	NA	0.103
Cfra	25,903	15,767	0.378	52	27	0.342	5.62E-01	0.853	FALSE	0.897
Sar3	23,723	13,956	0.370	226	91	0.287	2.33E-03	0.684	TRUE	1.823
Nk52	NA	NA	NA	NA	NA	NA	NA	NA	NA	0.040
Ncrx	NA	NA	NA	NA	NA	NA	NA	NA	NA	0.051
Spom	NA	NA	NA	NA	NA	NA	NA	NA	NA	0.097
Aory	NA	NA	NA	NA	NA	NA	NA	NA	NA	0.000
Cneo	NA	NA	NA	NA	NA	NA	NA	NA	NA	0.086
Umay	NA	NA	NA	NA	NA	NA	NA	NA	NA	0.000
Tmel	6,774	3,684	0.352	7	4	0.364	1.00E+00	1.051	FALSE	0.147
Rory	NA	NA	NA	NA	NA	NA	NA	NA	NA	0.023
Amac	NA	NA	NA	NA	NA	NA	NA	NA	NA	0.016
Spun	NA	NA	NA	NA	NA	NA	NA	NA	NA	0.034
Rirr	13,729	8,163	0.373	38	11	0.224	3.74E-02	0.487	FALSE	0.164
Ccor	NA	NA	NA	NA	NA	NA	NA	NA	NA	0.009
Gpro	NA	NA	NA	NA	NA	NA	NA	NA	NA	0.029
Falb	NA	NA	NA	NA	NA	NA	NA	NA	NA	0.000
Ddis2	NA	NA	NA	NA	NA	NA	NA	NA	NA	0.038
Ppal	NA	NA	NA	NA	NA	NA	NA	NA	NA	0.000
Acas	NA	NA	NA	NA	NA	NA	NA	NA	NA	0.033
Atha	41,473	34,587	0.455	71	49	0.408	3.15E-01	0.828	FALSE	0.430
Vvin	30,632	25,667	0.456	239	112	0.319	2.13E-07	0.559	TRUE	1.129
Mgut	37,281	30,622	0.451	98	52	0.347	1.08E-02	0.646	FALSE	0.536
Osat2	20,132	16,722	0.454	38	32	0.457	1.00E+00	1.014	FALSE	0.196
Ppat	30,423	25,670	0.458	266	130	0.328	2.11E-07	0.579	TRUE	1.193
Marpol	27,268	23,453	0.462	254	169	0.400	1.08E-02	0.774	FALSE	2.027
Smoe	NA	NA	NA	NA	NA	NA	NA	NA	NA	0.014
Kfla	26,808	21,736	0.448	42	34	0.447	1.00E+00	0.998	FALSE	0.435
Vcar	29,686	23,261	0.439	265	180	0.404	1.50E-01	0.867	FALSE	2.955
Crei	23,980	18,423	0.434	25	23	0.479	5.62E-01	1.197	FALSE	0.333
Mpus	836	713	0.460	9	4	0.308	4.03E-01	0.521	FALSE	0.131
Cpar	NA	NA	NA	NA	NA	NA	NA	NA	NA	0.027
Gthe	35,801	27,449	0.434	8	6	0.429	1.00E+00	0.978	FALSE	0.056
Esil	35,599	36,092	0.503	124	99	0.444	8.12E-02	0.787	FALSE	1.291
Pinf	NA	NA	NA	NA	NA	NA	NA	NA	NA	0.034
Aano	NA	NA	NA	NA	NA	NA	NA	NA	NA	0.017
Pfal2	2,946	1,850	0.386	14	10	0.417	8.34E-01	1.137	FALSE	0.411
Tthe	16,232	11,789	0.421	14	36	0.720	2.19E-05	3.540	TRUE	0.194
BnatMEA	59,443	37,511	0.387	199	92	0.316	1.34E-02	0.733	FALSE	1.189
Ehux	NA	NA	NA	NA	NA	NA	NA	NA	NA	0.013
Ngru	NA	NA	NA	NA	NA	NA	NA	NA	NA	0.019

NA=less than 10 exons in any group; discarded

Fig S12. Enrichment in 3n-divisbility of exon lengths in ES events, measured with Fisher's exact test. Includes data from:

(A) whole transcriptomes ('all')

(B) a sub-set of above-median gene expression and length ('high constraint' or 'HC' dataset)

(C) a sub-set with high ES frequency.

B) 'High constraint' gene sub-set (above median expression and gene length)

spi	ES-neg 3n-neg	ES-neg 3n-pos	% ES-neg 3n-pos	ES-pos 3n-neg	ES-pos 3n-pos	% ES-pos 3n-pos	Fisher pval	Fisher statistic	Is there any bias? P<0.01	ES-positive %
Hsap	26,873	17,662	0.397	436	750	0.632	1.53E-58	2.617	TRUE	10.538
Mmus	37,139	24,095	0.393	194	703	0.784	9.11E-124	5.585	TRUE	7.513
Mdom	29,868	19,763	0.398	184	628	0.773	2.47E-103	5.158	TRUE	7.043
Oana	19,701	13,174	0.401	132	289	0.686	9.50E-32	3.274	TRUE	4.249
Ggal	27,936	17,920	0.391	165	419	0.717	1.29E-56	3.959	TRUE	6.635
Xtro	25,321	17,075	0.403	58	206	0.780	1.97E-35	5.267	TRUE	3.226
Drex	42,245	27,523	0.394	135	525	0.795	2.30E-97	5.969	TRUE	5.178
Cint	8,696	5,366	0.382	90	71	0.441	1.42E-01	1.278	FALSE	3.551
Brabel	36,632	25,511	0.411	74	776	0.913	2.67E-207	15.057	TRUE	4.208
Spur2	12,338	8,526	0.409	367	276	0.429	3.09E-01	1.088	FALSE	5.343
Dmel	7,456	4,384	0.370	19	204	0.915	3.29E-64	18.257	TRUE	2.457
Cele	19,463	12,570	0.392	62	257	0.806	5.83E-51	6.418	TRUE	2.724
Cgig	23,774	16,250	0.406	64	337	0.840	6.10E-71	7.702	TRUE	2.116
Ctel	19,669	12,992	0.398	61	242	0.799	1.36E-45	6.006	TRUE	1.321
Sman	8,155	5,317	0.395	126	97	0.435	2.40E-01	1.181	FALSE	4.289
Tadh	NA	NA	NA	NA	NA	NA	NA	NA	NA	0.174
Hmag	16,787	11,831	0.413	65	75	0.536	4.37E-03	1.637	TRUE	1.641
Nvex	15,828	11,095	0.412	30	62	0.674	6.03E-07	2.948	TRUE	1.284
Aipt	15,824	11,908	0.429	14	39	0.736	9.14E-06	3.702	TRUE	0.420
Mlei	11,223	6,857	0.379	19	75	0.798	1.93E-16	6.460	TRUE	0.953
Aque	19,012	13,773	0.420	14	10	0.417	1.00E+00	0.986	FALSE	0.252
Ocar	NA	NA	NA	NA	NA	NA	NA	NA	NA	0.108
Scil	13,402	10,479	0.439	28	26	0.481	5.84E-01	1.188	FALSE	0.326
Sros	NA	NA	NA	NA	NA	NA	NA	NA	NA	0.069
Cowc	NA	NA	NA	NA	NA	NA	NA	NA	NA	0.103
Cfra	12,952	8,272	0.390	5	9	0.643	5.94E-02	2.818	FALSE	0.897
Sar3	11,882	7,145	0.376	62	27	0.303	1.88E-01	0.724	FALSE	1.823
Nk52	NA	NA	NA	NA	NA	NA	NA	NA	NA	0.040
Ncrx	NA	NA	NA	NA	NA	NA	NA	NA	NA	0.051
Spom	NA	NA	NA	NA	NA	NA	NA	NA	NA	0.097
Aory	NA	NA	NA	NA	NA	NA	NA	NA	NA	0.000
Cneo	NA	NA	NA	NA	NA	NA	NA	NA	NA	0.086
Umay	NA	NA	NA	NA	NA	NA	NA	NA	NA	0.000
Tmel	NA	NA	NA	NA	NA	NA	NA	NA	NA	0.147
Rory	NA	NA	NA	NA	NA	NA	NA	NA	NA	0.023
Amac	NA	NA	NA	NA	NA	NA	NA	NA	NA	0.016
Spun	NA	NA	NA	NA	NA	NA	NA	NA	NA	0.034
Rirr	7,008	4,069	0.367	10	6	0.375	1.00E+00	1.033	FALSE	0.164
Ccor	NA	NA	NA	NA	NA	NA	NA	NA	NA	0.009
Gpro	NA	NA	NA	NA	NA	NA	NA	NA	NA	0.029
Falb	NA	NA	NA	NA	NA	NA	NA	NA	NA	0.000
Ddis2	NA	NA	NA	NA	NA	NA	NA	NA	NA	0.038
Ppal	NA	NA	NA	NA	NA	NA	NA	NA	NA	0.000
Acas	NA	NA	NA	NA	NA	NA	NA	NA	NA	0.033
Atha	20,328	17,810	0.467	16	13	0.448	8.55E-01	0.927	FALSE	0.430
Vvin	15,286	13,034	0.460	69	35	0.337	1.34E-02	0.595	FALSE	1.129
Mgut	18,476	15,699	0.459	18	21	0.538	3.39E-01	1.373	FALSE	0.536
Osat2	10,156	8,556	0.457	8	11	0.579	3.58E-01	1.632	FALSE	0.196
Ppat	15,578	12,986	0.455	121	66	0.353	6.26E-03	0.654	TRUE	1.193
Marpol	13,750	11,719	0.460	66	90	0.577	3.69E-03	1.600	TRUE	2.027
Smoe	NA	NA	NA	NA	NA	NA	NA	NA	NA	0.014
Kfla	13,347	10,987	0.452	13	20	0.606	8.16E-02	1.869	FALSE	0.435
Vcar	14,841	11,731	0.441	124	91	0.423	6.30E-01	0.928	FALSE	2.955
Crei	12,044	9,337	0.437	5	11	0.688	7.41E-02	2.838	FALSE	0.333
Mpus	NA	NA	NA	NA	NA	NA	NA	NA	NA	0.131
Cpar	NA	NA	NA	NA	NA	NA	NA	NA	NA	0.027
Gthe	NA	NA	NA	NA	NA	NA	NA	NA	NA	0.056
Esil	17,890	18,220	0.505	28	25	0.472	6.81E-01	0.877	FALSE	1.291
Pinf	NA	NA	NA	NA	NA	NA	NA	NA	NA	0.034
Aano	NA	NA	NA	NA	NA	NA	NA	NA	NA	0.017
Pfal2	NA	NA	NA	NA	NA	NA	NA	NA	NA	0.411
Tthe	8,446	5,740	0.405	5	7	0.583	2.45E-01	2.060	FALSE	0.194
BnatMEA	29,557	19,009	0.391	122	44	0.265	7.78E-04	0.561	TRUE	1.189
Ehux	NA	NA	NA	NA	NA	NA	NA	NA	NA	0.013
Ngru	NA	NA	NA	NA	NA	NA	NA	NA	NA	0.019

NA=less than 10 exons in any group; discarded

Fig S12 (continued). Enrichment in 3n-divisbility of exon lengths in ES events, measured with Fisher's exact test. Includes data from:

(A) whole transcriptomes ('all')

(B) a sub-set of above-median gene expression and length ('high constraint' or 'HC' dataset)

(C) a sub-set with high ES frequency.

C) High ES frequency (rES = 30-70%)

spi	ES-neg 3n-neg	ES-neg 3n-pos	% ES-neg 3n-pos	ES-pos 3n-neg	ES-pos 3n-pos	% ES-pos 3n-pos	Fisher pval	Fisher statistic	Is there any bias? P<0.01	ES-positive %
Hsap	54,914	36,247	0.398	271	500	0.649	2.57E-44	2.795	TRUE	10.538
Mmus	74,149	49,507	0.400	123	531	0.812	1.66E-102	6.467	TRUE	7.513
Mdom	60,525	40,320	0.400	125	497	0.799	2.04E-91	5.968	TRUE	7.043
Oana	39,746	27,059	0.405	92	244	0.726	2.08E-32	3.896	TRUE	4.249
Ggal	56,017	36,834	0.397	91	340	0.789	1.36E-61	5.682	TRUE	6.635
Xtro	50,891	34,469	0.404	44	163	0.787	4.71E-29	5.469	TRUE	3.226
Drex	83,443	56,945	0.406	90	492	0.845	9.05E-106	8.009	TRUE	5.178
Cint	17,822	10,891	0.379	142	91	0.391	7.35E-01	1.049	FALSE	3.551
Brabel	73,944	51,779	0.412	61	545	0.899	1.98E-139	12.759	TRUE	4.208
Spur2	25,658	17,523	0.406	182	173	0.487	1.97E-03	1.392	TRUE	5.343
Dmel	15,175	8,875	0.369	21	126	0.857	4.44E-34	10.257	TRUE	2.457
Cele	39,278	25,282	0.392	46	153	0.769	5.48E-27	5.167	TRUE	2.724
Cgig	47,913	33,256	0.410	32	189	0.855	2.44E-42	8.508	TRUE	2.116
Ctel	39,746	26,339	0.399	27	172	0.864	6.02E-42	9.612	TRUE	1.321
Sman	16,627	10,820	0.394	65	44	0.404	8.45E-01	1.040	FALSE	4.289
Tadh	NA	NA	NA	NA	NA	NA	NA	NA	NA	0.174
Hmag	34,551	24,141	0.411	61	53	0.465	2.54E-01	1.244	FALSE	1.641
Nvex	32,468	21,892	0.403	25	37	0.597	2.61E-03	2.195	TRUE	1.284
Aipt	32,474	23,339	0.418	5	32	0.865	2.70E-08	8.904	TRUE	0.420
Mlei	22,738	13,899	0.379	15	60	0.800	1.35E-13	6.543	TRUE	0.953
Aque	37,387	28,475	0.432	7	19	0.731	2.56E-03	3.564	TRUE	0.252
Ocar	NA	NA	NA	NA	NA	NA	NA	NA	NA	0.108
Scil	27,546	20,598	0.428	8	16	0.667	2.24E-02	2.675	FALSE	0.326
Sros	NA	NA	NA	NA	NA	NA	NA	NA	NA	0.069
Cowc	NA	NA	NA	NA	NA	NA	NA	NA	NA	0.103
Cfra	26,557	16,135	0.378	11	10	0.476	3.74E-01	1.496	FALSE	0.897
Sar3	24,111	14,136	0.370	33	21	0.389	7.79E-01	1.085	FALSE	1.823
Nk52	NA	NA	NA	NA	NA	NA	NA	NA	NA	0.040
Ncrx	NA	NA	NA	NA	NA	NA	NA	NA	NA	0.051
Spom	NA	NA	NA	NA	NA	NA	NA	NA	NA	0.097
Aory	NA	NA	NA	NA	NA	NA	NA	NA	NA	0.000
Cneo	NA	NA	NA	NA	NA	NA	NA	NA	NA	0.086
Umay	NA	NA	NA	NA	NA	NA	NA	NA	NA	0.000
Tmel	NA	NA	NA	NA	NA	NA	NA	NA	NA	0.147
Rory	NA	NA	NA	NA	NA	NA	NA	NA	NA	0.023
Amac	NA	NA	NA	NA	NA	NA	NA	NA	NA	0.016
Spun	NA	NA	NA	NA	NA	NA	NA	NA	NA	0.034
Rirr	NA	NA	NA	NA	NA	NA	NA	NA	NA	0.164
Ccor	NA	NA	NA	NA	NA	NA	NA	NA	NA	0.009
Gpro	NA	NA	NA	NA	NA	NA	NA	NA	NA	0.029
Falb	NA	NA	NA	NA	NA	NA	NA	NA	NA	0.000
Ddis2	NA	NA	NA	NA	NA	NA	NA	NA	NA	0.038
Ppal	NA	NA	NA	NA	NA	NA	NA	NA	NA	0.000
Acas	NA	NA	NA	NA	NA	NA	NA	NA	NA	0.033
Atha	41,711	34,760	0.455	12	14	0.538	4.34E-01	1.400	FALSE	0.430
Vvin	30,997	25,861	0.455	40	24	0.375	2.11E-01	0.719	FALSE	1.129
Mgut	37,698	30,905	0.450	26	18	0.409	6.51E-01	0.844	FALSE	0.536
Osat2	20,512	17,011	0.453	9	4	0.308	4.06E-01	0.536	FALSE	0.196
Ppat	31,325	26,355	0.457	66	26	0.283	7.41E-04	0.468	TRUE	1.193
Marpol	27,565	23,600	0.461	57	68	0.544	7.22E-02	1.393	FALSE	2.027
Smoe	NA	NA	NA	NA	NA	NA	NA	NA	NA	0.014
Kfla	27,106	21,987	0.448	11	14	0.560	3.16E-01	1.569	FALSE	0.435
Vcar	30,591	23,880	0.438	41	38	0.481	4.96E-01	1.187	FALSE	2.955
Crei	NA	NA	NA	NA	NA	NA	NA	NA	NA	0.333
Mpus	NA	NA	NA	NA	NA	NA	NA	NA	NA	0.131
Cpar	NA	NA	NA	NA	NA	NA	NA	NA	NA	0.027
Gthe	NA	NA	NA	NA	NA	NA	NA	NA	NA	0.056
Esil	36,113	36,546	0.503	25	26	0.510	1.00E+00	1.028	FALSE	1.291
Pinf	NA	NA	NA	NA	NA	NA	NA	NA	NA	0.034
Aano	NA	NA	NA	NA	NA	NA	NA	NA	NA	0.017
Pfal2	NA	NA	NA	NA	NA	NA	NA	NA	NA	0.411
Tthe	16,539	12,056	0.422	3	12	0.800	3.57E-03	5.488	TRUE	0.194
BnatMEA	60,592	38,185	0.387	41	29	0.414	6.26E-01	1.122	FALSE	1.189
Ehux	NA	NA	NA	NA	NA	NA	NA	NA	NA	0.013
Ngru	NA	NA	NA	NA	NA	NA	NA	NA	NA	0.019

NA=less than 10 exons in any group; discarded

Fig S12 (contiuned). Enrichment in 3n-divisbility of exon lengths in ES events, measured with Fisher's exact test. Includes data from:

(A) whole transcriptomes ('all')

(B) a sub-set of above-median gene expression and length ('high constraint' or 'HC' dataset)

(C) a sub-set with high ES frequency.

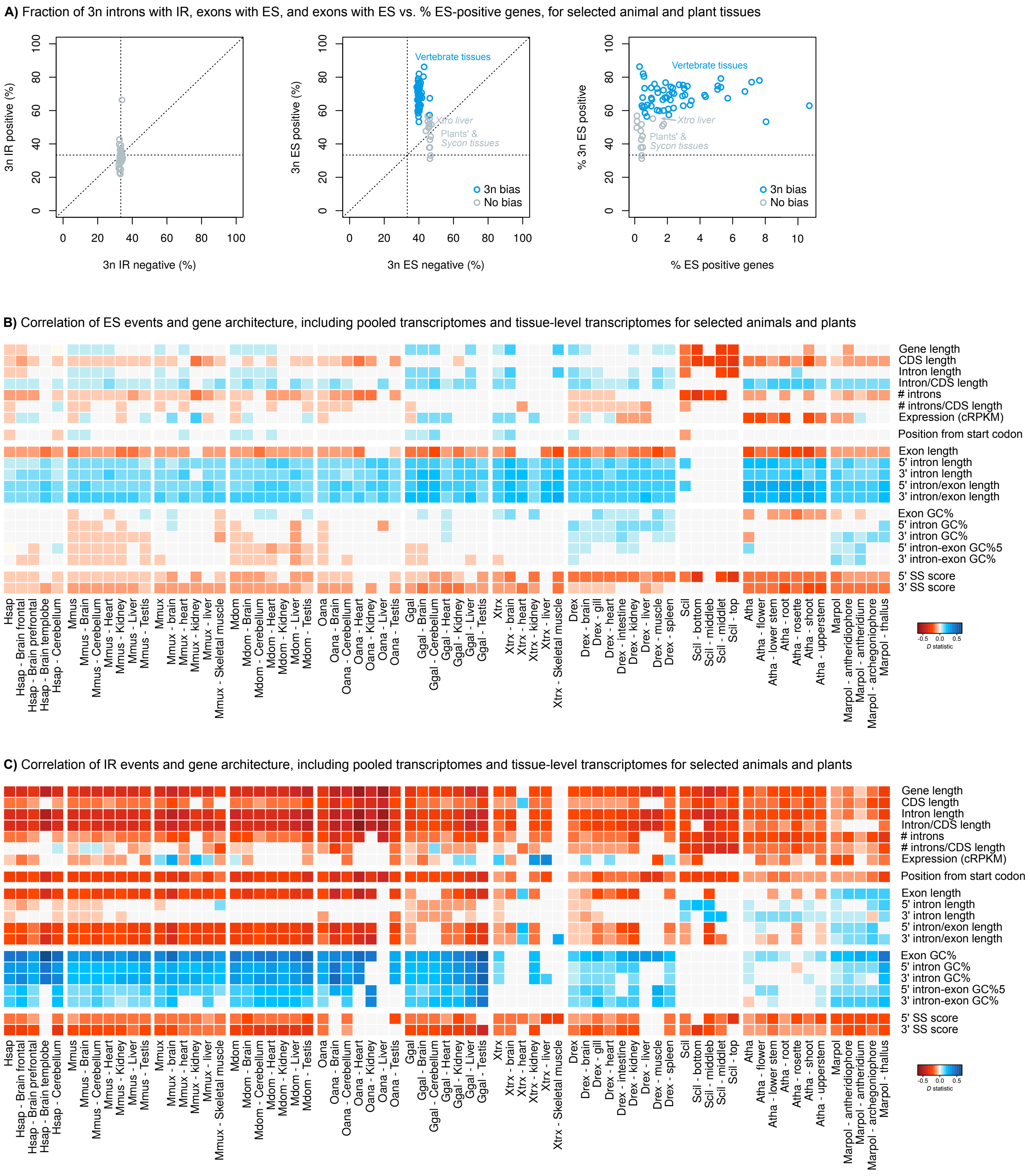

Fig S13. Tissue-level analysis of gene architectural effects on AS, using samples from selected animals (*H. sapiens*, 2x *M. musculus, M. domestica, O. anatinus, G. gallus, X. tropicalis, D. rerio, S. ciliatum*) and plants (*A. thaliana* and *M. polymorpha*).

A) Fraction of 3-divisible introns and exons involved in AS events (as in Figure 3).

B) Relationship between ES and gene architectural traits, as in Figure 4.

C) Relationship between IR and gene architectural traits, as in Figure 5.

Each heatmap represents the distance between distributions of gene architecture values for ES- or IR-positive and -negative exons or introns, measured with the D statistic of the one-sided Kolmogorov-Smirnov two-sample test (significant if $p<0.01$; otherwise grey). D values are recorded as positive/negative (blue/red) according to two one-sided Kolmogorov-Smirnov tests with complementary alternative hypotheses: positive/negative D (blue/red) reflects a positive/negative relationship between ES or IR and the given feature.

A) Fraction of 3n introns with IR, exons with ES, and exons with ES vs. % ES-positive genes, transcriptome at 2x sequencing depth

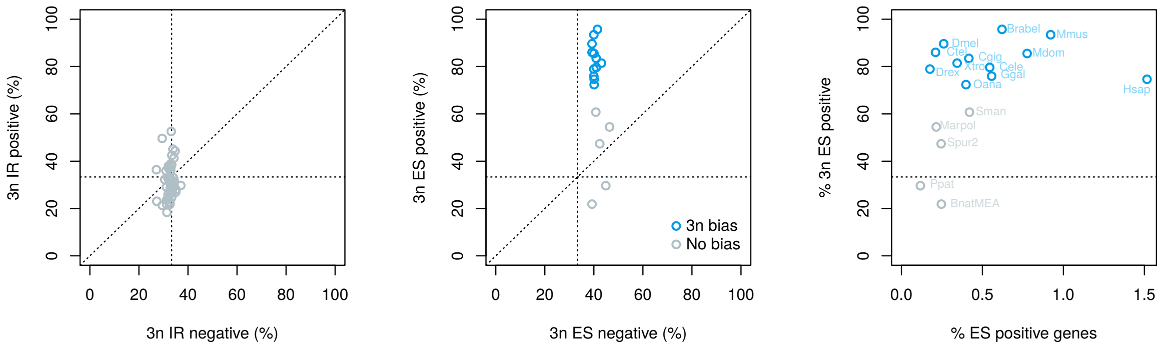

C) Fraction of 3n introns with IR, exons with ES, and exons with ES vs. % ES-positive genes, transcriptome at 5x sequencing depth

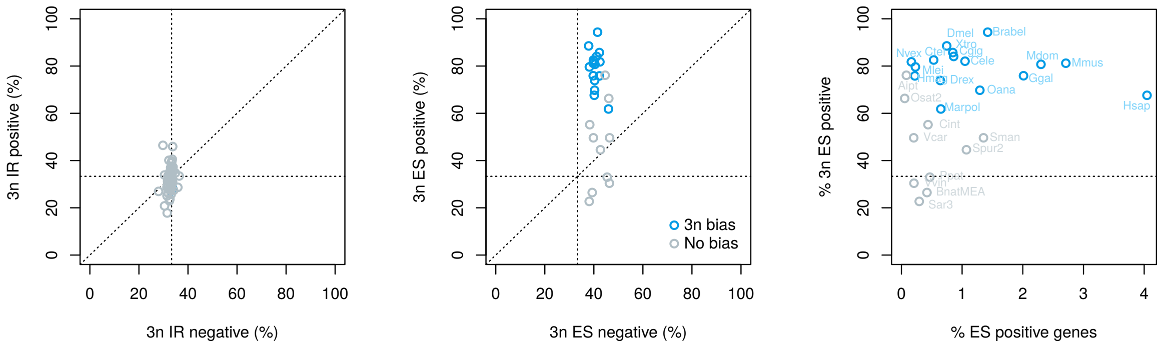

C) Fraction of 3n introns with IR, exons with ES, and exons with ES vs. % ES-positive genes, transcriptome at 10x sequencing depth

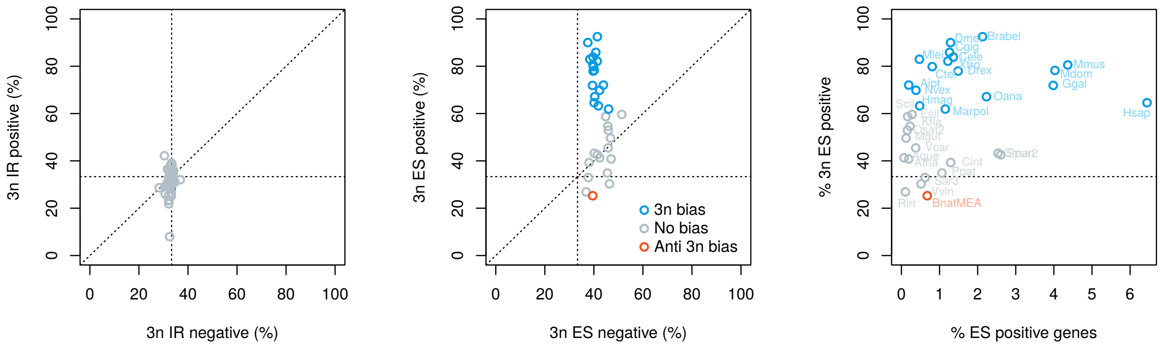

D) Fraction of 3n introns with IR, exons with ES, and exons with ES vs. % ES-positive genes, transcriptome at 15x sequencing depth

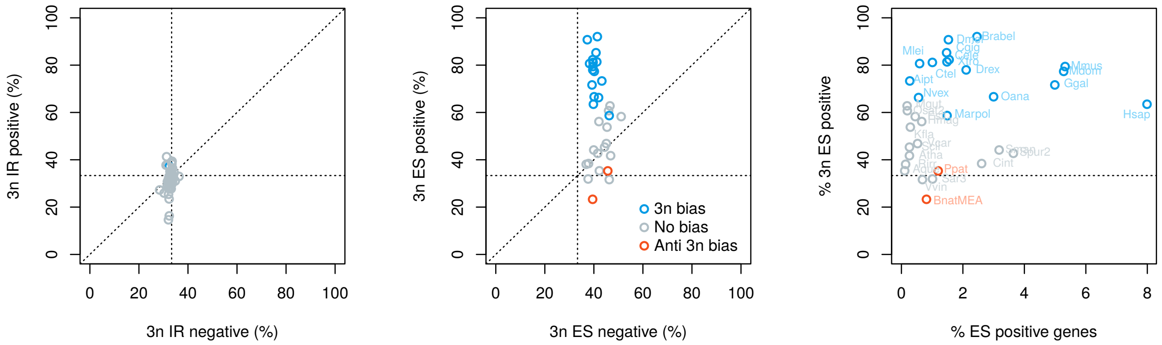

Fig S14. Fraction of 3-divisible introns and exons involved in AS events (as in Figure 3), downsampling each RNA-seq sample at 2x (A), 5x (B), 10x (C), and 15x (D).

In each case, we report 1) % of introns with 3-divisible lengths that were classified as IR-positive (x-axis) and IR-negative (y-axis), 2) % exons with 3-divisible lengths that were classified as ES-negative (x-axis) and ES-negative (y-axis) in each species with >10 ES-positive exons; 3) ES-positive exons that are 3n (y-axis) with respect to the percentage of genes with ES-positive exons (x-axis), per species.

species	Architectural feature	oKS p-value	oKS D statistic
Hsap	lengene	1.89E-05	-0.04677
Hsap	lencds	7.46E-28	-0.11208
Hsap	lenints	4.58E-04	-0.03932
Hsap	ralincd	1.25E-04	0.04251
Hsap	numin	1.78E-31	-0.11933
Hsap	ranincd	2.38E-04	-0.04097
Hsap	crpkm	1.74E-10	-0.06723
Hsap	posg	2.92E-04	-0.04046
Hsap	lenex	5.36E-51	-0.15257
Hsap	lenin1	4.32E-19	0.09222
Hsap	lenin2	4.03E-20	0.09477
Hsap	ralinex1	1.08E-40	0.13605
Hsap	ralinex2	8.98E-45	0.14282
Hsap	gcex	NA	NA
Hsap	gcin1	NA	NA
[illegible]	[illegible]	[illegible]	[illegible]
Ggal	lenin2	9.74E-67	0.25384
Ggal	ralinex1	1.58E-71	0.26289
Ggal	ralinex2	2.19E-82	0.28233
Ggal	gcex	NA	NA
Ggal	gcin1	NA	NA
Ggal	gcin2	NA	NA
Ggal	difginex1	6.10E-04	-0.05602
Ggal	difginex2	2.21E-03	-0.05091
Ggal	ss5score	9.79E-10	-0.09378
Ggal	ss3score	6.73E-30	-0.16875

species	Architectural feature	oKS p-value	oKS D statistic
Xtro	lengene	1.58E-06	0.10161
Xtro	lencds	6.37E-05	-0.08641
Xtro	lenints	2.97E-07	0.10778
Xtro	ralincd	1.01E-15	0.16335
Xtro	numin	9.78E-06	-0.09442
Xtro	ranincd	2.83E-04	-0.07946
Xtro	crpkm	2.26E-08	-0.11665
Xtro	posg	4.05E-04	0.07770
Xtro	lenex	5.06E-26	-0.21217
Xtro	lenin1	2.24E-29	0.22580
Xtro	lenin2	3.26E-35	0.24773
Xtro	ralinex1	1.22E-44	0.27954
Xtro	ralinex2	4.29E-48	0.29033
Xtro	gcex	2.09E-03	0.06905
Xtro	gcin1	1.83E-03	0.06979
[illegible]	[illegible]	[illegible]	[illegible]
Spur2	lenin2	1.01E-03	0.04390
Spur2	ralinex1	2.85E-11	0.08231
Spur2	ralinex2	2.67E-10	0.07845
Spur2	gcex	2.11E-40	-0.15966
Spur2	gcin1	NA	NA
Spur2	gcin2	4.11E-04	-0.04666
Spur2	difginex1	7.36E-26	0.12707
Spur2	difginex2	7.66E-27	0.12957
Spur2	ss5score	4.28E-12	-0.08546
Spur2	ss3score	8.95E-33	-0.14353

species	Architectural feature	oKS p-value	oKS D statistic
[illegible]	[illegible]	[illegible]	[illegible]

One-sided two-sample Kolmogorov-Smirnov test
Alternative hypothesis 1: the CDF of positive events lies **below** that of negative events
Alternative hypothesis 2: the CDF of positive events lies **above** that of negative events
If AH1 is TRUE, D>0
If AH2 is TRUE, D<0
If both are FALSE, D=NA
If both are TRUE, D=NA (marginal pvalues)

lengene	gene length
lencds	CDS length
lenints	total intron length
ralincd	ration intron length/CDS length
numin	number of introns in gene
ranincd	number of introns/CDS length
lenex	exon length
lenin1	5' intron length
lenin2	3' intron length
ralinex1	5' intron length/exon length
ralinex2	3' intron length/exon length
gcex	exon GC content
gcin1	5' intron GC content
gcin2	3' intron GC content
difginex1	Exon-5' intron GC content
difginex2	Exon-3' intron GC content
ss5score	5' SS heterogeneity score
ss3score	3' SS heterogeneity score
crpkm	expression level of transcript (cRPKM)
posg	position since start of gene

Fig S15. Species-specific comparisons of ES events with gene architectural features using dual Kolmogorov-Smirnov 2-sample tests with complementary alternative hypothesis (see Methods). D statistic encoded as positive/negative if either hypothesis is significant (p<0.01), NA if otherwise.

species	Architectural feature	oKS p-value	oKS D statistic
Hsap	lengene	0.00E+00	-0.31197
Hsap	lencds	4.93E-47	-0.11459
Hsap	lenints	0.00E+00	-0.31337
Hsap	ralincd	1.7292297604	-0.30206
Hsap	numin	7.29E-50	-0.11804
Hsap	ranincd	NA	NA
Hsap	crpkm	4.99E-06	-0.03877
Hsap	posg	1.26E-180	-0.22586
Hsap	lenin	1.04E-153	-0.20828
Hsap	lenex1	5.13E-04	-0.03054
Hsap	lenex2	6.39E-03	-0.02495
Hsap	ralinex1	5.93E-136	-0.19582
Hsap	ralinex2	2.16E-142	-0.20043
Hsap	gcin	0.00E+00	0.36595
Hsap	gcex1	5.27E-293	0.28789
Hsap	gcex2	1.78E-289	0.28615
Hsap	difginex1	2.68E-66	0.13636
Hsap	difginex2	1.24E-61	0.13142
Hsap	ss5score	8.96E-49	-0.11672
Hsap	ss3score	2.02E-157	-0.21079
Hsax	lengene	3.75E-25	-0.04850
Hsax	lencds	NA	NA
Hsax	lenints	1.72E-23	-0.04683
Hsax	ralincd	1.68E-22	-0.04580
Hsax	numin	1.27E-08	-0.02758
Hsax	ranincd	3.09E-22	-0.04552
Hsax	crpkm	7.24E-104	-0.09967
Hsax	posg	4.77E-09	-0.02831
Hsax	lenin	6.61E-07	0.02440
Hsax	lenex1	NA	NA
Hsax	lenex2	1.03E-13	-0.03537
Hsax	ralinex1	9.17E-04	0.01711
Hsax	ralinex2	3.94E-06	0.02282
Hsax	gcin	NA	NA
Hsax	gcex1	2.95E-20	0.04337
Hsax	gcex2	1.52E-19	0.04258
Hsax	difginex1	1.93E-07	-0.02543
Hsax	difginex2	5.94E-05	-0.02018
Hsax	ss5score	8.51E-11	-0.03114
Hsax	ss3score	1.55E-19	-0.04256
Mmus	lengene	0.00E+00	-0.29046
Mmus	lencds	1.16E-69	-0.11566
Mmus	lenints	0.00E+00	-0.29199
Mmus	ralincd	0.00E+00	-0.29238
Mmus	numin	3.51E-60	-0.10741
Mmus	ranincd	8.05E-08	-0.03710
Mmus	crpkm	1.30E-26	-0.07088
Mmus	posg	1.01E-252	-0.22114
Mmus	lenin	3.51E-179	-0.18609
Mmus	lenex1	2.48E-08	-0.03842
Mmus	lenex2	2.75E-06	-0.03285
Mmus	ralinex1	5.37E-154	-0.17246
Mmus	ralinex2	8.26E-149	-0.16952
Mmus	gcin	0.00E+00	0.28469
Mmus	gcex1	9.25E-197	0.19504
Mmus	gcex2	3.93E-198	0.19573
Mmus	difginex1	8.73E-30	0.07509
Mmus	difginex2	4.87E-32	0.07795
Mmus	ss5score	1.44E-47	-0.09534
Mmus	ss3score	3.61E-133	-0.16032
Mmux	lengene	8.48E-194	-0.32560
Mmux	lencds	1.98E-34	-0.13604
Mmux	lenints	3.19E-199	-0.33014
Mmux	ralincd	1.09E-185	-0.31869
Mmux	numin	2.23E-30	-0.12760
Mmux	ranincd	NA	NA
Mmux	crpkm	1.56E-14	0.08707
Mmux	posg	2.83E-107	-0.24188
Mmux	lenin	3.47E-109	-0.24404
Mmux	lenex1	1.77E-03	-0.03888
Mmux	lenex2	NA	NA
Mmux	ralinex1	1.42E-96	-0.22941
Mmux	ralinex2	6.28E-96	-0.22864
Mmux	gcin	1.42E-173	0.30808
Mmux	gcex1	6.85E-69	0.19347
Mmux	gcex2	2.91E-82	0.21159
Mmux	difginex1	5.33E-28	0.12238
Mmux	difginex2	4.67E-27	0.12024
Mmux	ss5score	4.49E-25	-0.11563
Mmux	ss3score	3.82E-48	-0.16136
Mdom	lengene	1.63E-272	-0.33387
Mdom	lencds	4.98E-47	-0.13781
Mdom	lenints	2.16E-277	-0.33686
Mdom	ralincd	5.58E-265	-0.32921
Mdom	numin	3.88E-61	-0.15741
Mdom	ranincd	1.41E-08	-0.05675
Mdom	crpkm	1.15E-05	0.04501
Mdom	posg	1.70E-157	-0.25357
Mdom	lenin	5.99E-144	-0.24237
Mdom	lenex1	NA	NA
Mdom	lenex2	NA	NA
Mdom	ralinex1	2.21E-123	-0.22429
Mdom	ralinex2	8.49E-107	-0.20858
Mdom	gcin	2.09E-300	0.35059
Mdom	gcex1	5.37E-186	0.27566
Mdom	gcex2	9.07E-196	0.28283
Mdom	difginex1	1.22E-39	0.12633
Mdom	difginex2	1.37E-43	0.13259
Mdom	ss5score	2.06E-42	-0.13076
Mdom	ss3score	6.69E-144	-0.24233
Oana	lengene	8.66E-32	-0.28175
Oana	lencds	7.60E-13	-0.17599
Oana	lenints	3.52E-32	-0.28352
Oana	ralincd	9.23E-34	-0.29056
Oana	numin	3.55E-15	-0.19217
Oana	ranincd	3.62E-05	-0.10654
Oana	crpkm	NA	NA
Oana	posg	6.56E-13	-0.17645
Oana	lenin	3.70E-13	-0.17825
Oana	lenex1	NA	NA
Oana	lenex2	3.13E-04	-0.09465
Oana	ralinex1	3.37E-14	-0.18556
Oana	ralinex2	3.35E-10	-0.15561
Oana	gcin	2.23E-26	0.25604
Oana	gcex1	9.52E-22	0.23178
Oana	gcex2	4.79E-23	0.23883
Oana	difginex1	2.75E-05	0.10797
Oana	difginex2	7.28E-03	0.07391
Oana	ss5score	1.55E-07	-0.13193
Oana	ss3score	7.16E-07	-0.12532
Ggal	lengene	1.22E-174	-0.26091
Ggal	lencds	1.89E-69	-0.16401
Ggal	lenints	8.32E-176	-0.26178
Ggal	ralincd	5.79E-135	-0.22922
Ggal	numin	1.28E-59	-0.15183
Ggal	ranincd	4.67E-06	-0.04568
Ggal	crpkm	2.10E-06	-0.04715
Ggal	posg	1.37E-99	-0.19671
Ggal	lenin	5.06E-32	-0.11068
Ggal	lenex1	1.84E-07	-0.05135
Ggal	lenex2	6.71E-05	-0.04042
Ggal	ralinex1	3.34E-23	-0.09380
Ggal	ralinex2	1.83E-24	-0.09639
Ggal	gcin	9.48E-155	0.24553
Ggal	gcex1	2.36E-100	0.19747
Ggal	gcex2	2.10E-101	0.19851
Ggal	difginex1	2.10E-48	0.13661
Ggal	difginex2	1.81E-55	0.14638
Ggal	ss5score	3.90E-35	-0.11605
Ggal	ss3score	1.42E-93	-0.19064

species	Architectural feature	oKS p-value	oKS D statistic
Xtro	lengene	9.20E-23	-0.20106
Xtro	lencds	7.91E-05	-0.08674
Xtro	lenints	3.74E-23	-0.20283
Xtro	ralincd	4.20E-16	-0.16795
Xtro	numin	8.66E-10	-0.12893
Xtro	ranincd	2.10E-09	-0.12618
Xtro	crpkm	1.02E-03	-0.07405
Xtro	posg	1.00E-10	-0.13544
Xtro	lenin	4.26E-06	-0.09925
Xtro	lenex1	1.14E-05	0.09523
Xtro	lenex2	4.03E-06	0.09948
Xtro	ralinex1	7.10E-07	-0.10620
Xtro	ralinex2	5.16E-07	-0.10740
Xtro	gcin	4.55E-31	0.23592
Xtro	gcex1	3.64E-25	0.21173
Xtro	gcex2	2.91E-22	0.19876
Xtro	difginex1	7.95E-05	0.08672
Xtro	difginex2	1.02E-06	0.10482
Xtro	ss5score	2.16E-11	-0.13987
Xtro	ss3score	4.14E-26	-0.21578
Xtrx	lengene	2.52E-31	-0.16725
Xtrx	lencds	7.70E-11	-0.09615
Xtrx	lenints	9.79E-32	-0.16837
Xtrx	ralincd	6.91E-17	-0.12155
Xtrx	numin	1.50E-14	-0.11242
Xtrx	ranincd	5.30E-04	-0.05472
Xtrx	crpkm	5.32E-21	0.13614
Xtrx	posg	9.44E-12	-0.10039
Xtrx	lenin	2.69E-07	-0.07750
Xtrx	lenex1	5.46E-03	-0.04549
Xtrx	lenex2	3.86E-03	-0.04697
Xtrx	ralinex1	2.18E-06	-0.07195
Xtrx	ralinex2	8.53E-06	-0.06807
Xtrx	gcin	1.03E-15	0.11705
Xtrx	gcex1	2.13E-14	0.11180
Xtrx	gcex2	5.36E-12	0.10151
Xtrx	difginex1	NA	NA
Xtrx	difginex2	NA	NA
Xtrx	ss5score	2.62E-19	-0.13034
Xtrx	ss3score	1.10E-19	-0.13165
Drex	lengene	9.62E-59	-0.18271
Drex	lencds	1.75E-21	-0.10929
Drex	lenints	2.76E-62	-0.18820
Drex	ralincd	3.05E-30	-0.13032
Drex	numin	4.90E-34	-0.13844
Drex	ranincd	7.50E-10	-0.07246
Drex	crpkm	NA	NA
Drex	posg	6.34E-16	-0.09351
Drex	lenin	2.13E-06	-0.05712
Drex	lenex1	5.62E-06	-0.05496
Drex	lenex2	4.44E-04	-0.04392
Drex	ralinex1	7.76E-05	-0.04863
Drex	ralinex2	1.57E-03	-0.04016
Drex	gcin	3.21E-24	0.11626
Drex	gcex1	3.23E-05	0.05083
Drex	gcex2	1.71E-04	0.04656
Drex	difginex1	2.63E-11	0.07802
Drex	difginex2	1.54E-12	0.08244
Drex	ss5score	2.63E-09	-0.07026
Drex	ss3score	3.73E-30	-0.13012
Drer	lengene	1.26E-46	-0.27392
Drer	lencds	6.50E-21	-0.18166
Drer	lenints	1.14E-49	-0.28286
Drer	ralincd	6.85E-39	-0.24978
Drer	numin	3.77E-41	-0.25706
Drer	ranincd	8.33E-16	-0.15700
Drer	crpkm	6.16E-37	-0.24330
Drer	posg	2.38E-18	-0.16973
Drer	lenin	1.50E-08	-0.11309
Drer	lenex1	1.08E-07	0.10672
Drer	lenex2	2.07E-03	0.06624
Drer	ralinex1	1.08E-09	-0.12108
Drer	ralinex2	1.60E-06	-0.09733
Drer	gcin	5.53E-10	0.12302
Drer	gcex1	1.53E-03	0.06784
Drer	gcex2	1.24E-04	0.07992
Drer	difginex1	9.03E-05	0.08131
Drer	difginex2	7.52E-05	0.08211
Drer	ss5score	3.39E-08	-0.11050
Drer	ss3score	4.70E-15	-0.15304
Cint	lengene	1.38E-03	-0.09143
Cint	lencds	NA	NA
Cint	lenints	1.55E-05	-0.11854
Cint	ralincd	8.98E-06	-0.12144
Cint	numin	NA	NA
Cint	ranincd	NA	NA
Cint	crpkm	4.54E-23	-0.25551
Cint	posg	NA	NA
Cint	lenin	6.53E-12	-0.18078
Cint	lenex1	NA	NA
Cint	lenex2	NA	NA
Cint	ralinex1	2.37E-06	-0.12821
Cint	ralinex2	6.75E-10	-0.16369
Cint	gcin	1.20E-07	-0.14219
Cint	gcex1	2.29E-05	-0.11645
Cint	gcex2	NA	NA
Cint	difginex1	NA	NA
Cint	difginex2	1.28E-04	-0.10665
Cint	ss5score	4.28E-08	-0.14673
Cint	ss3score	NA	NA
Brabel	lengene	8.96E-10	-0.06367
Brabel	lencds	4.62E-22	-0.09777
Brabel	lenints	9.68E-08	-0.05606
Brabel	ralincd	NA	NA
Brabel	numin	1.29E-21	-0.09675
Brabel	ranincd	4.01E-08	-0.05757
Brabel	crpkm	NA	NA
Brabel	posg	9.32E-03	-0.03016
Brabel	lenin	9.68E-56	0.15700
Brabel	lenex1	NA	NA
Brabel	lenex2	NA	NA
Brabel	ralinex1	2.32E-55	0.15646
Brabel	ralinex2	9.40E-56	0.15702
Brabel	gcin	1.80E-28	0.11150
Brabel	gcex1	NA	NA
Brabel	gcex2	NA	NA
Brabel	difginex1	3.49E-10	0.06509
Brabel	difginex2	1.05E-15	0.08193
Brabel	ss5score	2.59E-20	-0.09368
Brabel	ss3score	2.21E-27	-0.10928
Spur2	lengene	6.38E-03	-0.09247
Spur2	lencds	NA	NA
Spur2	lenints	3.94E-03	-0.09679
Spur2	ralincd	5.21E-05	-0.12917
Spur2	numin	2.84E-04	-0.11754
Spur2	ranincd	7.56E-06	-0.14124
Spur2	crpkm	NA	NA
Spur2	posg	1.85E-03	0.10319
Spur2	lenin	7.44E-07	0.15451
Spur2	lenex1	NA	NA
Spur2	lenex2	3.89E-03	0.09690
Spur2	ralinex1	5.48E-04	0.11272
Spur2	ralinex2	4.53E-04	0.11412
Spur2	gcin	NA	NA
Spur2	gcex1	NA	NA
Spur2	gcex2	NA	NA
Spur2	difginex1	NA	NA
Spur2	difginex2	NA	NA
Spur2	ss5score	2.04E-06	-0.14887
Spur2	ss3score	2.41E-03	-0.10097

species	Architectural feature	oKS p-value	oKS D statistic
Dmel	lengene	5.73E-13	0.06704
Dmel	lencds	4.84E-03	-0.02915
Dmel	lenints	8.58E-22	0.08794
Dmel	ralincd	3.88E-21	0.08656
Dmel	numin	NA	NA
Dmel	ranincd	4.03E-05	0.04016
Dmel	crpkm	9.10E-201	-0.27098
Dmel	posg	6.20E-04	-0.03431
Dmel	lenin	2.71E-175	0.25314
Dmel	lenex1	1.43E-19	-0.08317
Dmel	lenex2	9.76E-09	-0.05423
Dmel	ralinex1	1.40E-166	0.24674
Dmel	ralinex2	4.55E-145	0.23018
Dmel	gcin	5.49E-47	0.13031
Dmel	gcex1	6.01E-13	0.06698
Dmel	gcex2	1.28E-09	0.05713
Dmel	difginex1	1.02E-21	0.08778
Dmel	difginex2	4.41E-21	0.08644
Dmel	ss5score	3.50E-17	-0.07772
Dmel	ss3score	1.70E-36	-0.11458
Dmel2	lengene	6.42E-15	0.14226
Dmel2	lencds	2.16E-03	0.06165
Dmel2	lenints	3.34E-24	0.18296
Dmel2	ralincd	2.66E-25	0.18719
Dmel2	numin	2.26E-13	0.13428
Dmel2	ranincd	9.68E-09	0.10690
Dmel2	crpkm	NA	NA
Dmel2	posg	4.64E-14	0.13788
Dmel2	lenin	6.13E-104	0.38362
Dmel2	lenex1	8.98E-09	-0.10711
Dmel2	lenex2	1.70E-13	-0.13493
Dmel2	ralinex1	1.04E-82	0.34190
Dmel2	ralinex2	1.42E-86	0.34987
Dmel2	gcin	4.43E-21	0.17036
Dmel2	gcex1	NA	NA
Dmel2	gcex2	1.24E-03	-0.06436
Dmel2	difginex1	1.17E-08	0.10636
Dmel2	difginex2	2.03E-10	0.11756
Dmel2	ss5score	6.44E-18	-0.15656
Dmel2	ss3score	3.70E-35	-0.22157
Cele	lengene	5.74E-109	-0.11670
Cele	lencds	4.74E-122	-0.12355
Cele	lenints	1.60E-56	-0.08378
Cele	ralincd	NA	NA
Cele	numin	3.59E-36	-0.06678
Cele	ranincd	2.13E-87	0.10442
Cele	crpkm	0.00E+00	-0.36402
Cele	posg	1.51E-48	-0.07757
Cele	lenin	NA	NA
Cele	lenex1	7.49E-70	-0.09326
Cele	lenex2	7.34E-55	-0.08253
Cele	ralinex1	3.52E-25	0.05547
Cele	ralinex2	3.67E-21	0.05071
Cele	gcin	1.99E-89	0.10564
Cele	gcex1	2.82E-70	-0.09354
Cele	gcex2	6.14E-66	-0.09058
Cele	difginex1	1.00E-166	0.14452
Cele	difginex2	2.01E-165	0.14395
Cele	ss5score	1.07E-20	-0.05013
Cele	ss3score	6.07E-99	-0.11116
Cgig	lengene	NA	NA
Cgig	lencds	1.00E-04	-0.06882
Cgig	lenints	NA	NA
Cgig	ralincd	7.67E-10	0.10390
Cgig	numin	8.93E-07	-0.08464
Cgig	ranincd	1.00E-05	-0.07694
Cgig	crpkm	7.50E-04	0.06083
Cgig	posg	1.33E-03	0.05837
Cgig	lenin	2.32E-93	0.33122
Cgig	lenex1	4.30E-04	-0.06314
Cgig	lenex2	NA	NA
Cgig	ralinex1	8.40E-70	0.28602
Cgig	ralinex2	7.20E-71	0.28822
Cgig	gcin	2.12E-39	0.21401
Cgig	gcex1	5.02E-08	0.09297
Cgig	gcex2	3.66E-13	0.12136
Cgig	difginex1	NA	NA
Cgig	difginex2	NA	NA
Cgig	ss5score	NA	NA
Cgig	ss3score	4.01E-18	-0.14354
Ctel	lengene	7.77E-03	-0.03429
Ctel	lencds	8.54E-09	-0.06705
Ctel	lenints	NA	NA
Ctel	ralincd	1.49E-09	0.07013
Ctel	numin	1.40E-08	-0.06616
Ctel	ranincd	NA	NA
Ctel	crpkm	NA	NA
Ctel	posg	4.12E-03	0.03646
Ctel	lenin	6.64E-147	0.28539
Ctel	lenex1	1.05E-05	-0.05268
Ctel	lenex2	4.03E-04	-0.04349
Ctel	ralinex1	1.52E-103	0.23935
Ctel	ralinex2	3.24E-99	0.23426
Ctel	gcin	3.84E-12	0.07975
Ctel	gcex1	1.41E-03	0.03986
Ctel	gcex2	5.89E-04	0.04242
Ctel	difginex1	2.74E-04	0.04455
Ctel	difginex2	6.30E-04	0.04223
Ctel	ss5score	4.78E-12	-0.07942
Ctel	ss3score	2.24E-08	-0.06528
Sman	lengene	4.80E-71	-0.28943
Sman	lencds	7.76E-50	-0.24188
Sman	lenints	5.41E-70	-0.28726
Sman	ralincd	3.59E-27	-0.17749
Sman	numin	1.63E-36	-0.20648
Sman	ranincd	5.17E-09	0.09935
Sman	crpkm	3.38E-08	0.09434
Sman	posg	8.22E-89	-0.32393
Sman	lenin	2.27E-114	-0.36794
Sman	lenex1	4.29E-15	-0.13083
Sman	lenex2	4.08E-07	-0.08725
Sman	ralinex1	1.39E-105	-0.35343
Sman	ralinex2	1.87E-106	-0.35490
Sman	gcin	2.10E-03	-0.05648
Sman	gcex1	6.76E-08	0.09242
Sman	gcex2	8.95E-17	0.13827
Sman	difginex1	1.69E-08	-0.09623
Sman	difginex2	8.42E-12	-0.11486
Sman	ss5score	4.54E-44	-0.22723
Sman	ss3score	5.53E-49	-0.23977
Tadh	lengene	9.84E-06	-0.10874
Tadh	lencds	1.08E-07	-0.12827
Tadh	lenints	9.62E-04	-0.08441
Tadh	ralincd	1.42E-03	0.08200
Tadh	numin	6.59E-11	-0.15506
Tadh	ranincd	1.66E-06	-0.11683
Tadh	crpkm	1.45E-09	-0.14448
Tadh	posg	1.31E-03	-0.08252
Tadh	lenin	5.08E-20	0.21346
Tadh	lenex1	NA	NA
Tadh	lenex2	NA	NA
Tadh	ralinex1	2.38E-14	0.17937
Tadh	ralinex2	8.36E-14	0.17574
Tadh	gcin	2.65E-09	0.14233
Tadh	gcex1	NA	NA
Tadh	gcex2	NA	NA
Tadh	difginex1	6.65E-07	0.12078
Tadh	difginex2	6.95E-06	0.11037
Tadh	ss5score	4.02E-08	-0.13216
Tadh	ss3score	2.69E-03	-0.07792

species	Architectural feature	oKS p-value	oKS D statistic
Hmag	lengene	1.97E-21	-0.33413
Hmag	lencds	2.00E-06	-0.17529
Hmag	lenints	1.24E-20	-0.32761
Hmag	ralincd	1.11E-11	-0.24303
Hmag	numin	1.07E-15	-0.28411
Hmag	ranincd	3.33E-11	-0.23768
Hmag	crpkm	NA	NA
Hmag	posg	2.10E-10	-0.22844
Hmag	lenin	3.84E-03	-0.11414
Hmag	lenex1	1.33E-03	0.12456
Hmag	lenex2	2.32E-04	0.13997
Hmag	ralinex1	1.71E-03	-0.12213
Hmag	ralinex2	1.47E-03	-0.12358
Hmag	gcin	4.50E-03	0.11249
Hmag	gcex1	NA	NA
Hmag	gcex2	NA	NA
Hmag	difginex1	1.07E-05	0.16373
Hmag	difginex2	NA	NA
Hmag	ss5score	NA	NA
Hmag	ss3score	2.34E-04	-0.13992
Nvec	lengene	2.87E-14	-0.09313
Nvec	lencds	7.01E-17	-0.10172
Nvec	lenints	1.10E-13	-0.09110
Nvec	ralincd	8.20E-03	-0.03656
Nvec	numin	2.25E-25	-0.12565
Nvec	ranincd	1.38E-13	-0.09076
Nvec	crpkm	5.13E-132	-0.28999
Nvec	posg	2.01E-15	-0.09702
Nvec	lenin	2.20E-34	0.14683
Nvec	lenex1	NA	NA
Nvec	lenex2	1.58E-03	0.04235
Nvec	ralinex1	2.56E-23	0.12029
Nvec	ralinex2	1.44E-16	0.10073
Nvec	gcin	1.51E-13	0.09061
Nvec	gcex1	NA	NA
Nvec	gcex2	2.04E-08	-0.07019
Nvec	difginex1	8.75E-13	0.08788
Nvec	difginex2	1.29E-21	0.11567
Nvec	ss5score	2.82E-10	-0.07821
Nvec	ss3score	1.64E-06	-0.06087
Nvex	lengene	2.27E-27	-0.14638
Nvex	lencds	1.55E-15	-0.10913
Nvex	lenints	1.16E-27	-0.14718
Nvex	ralincd	1.35E-11	-0.09349
Nvex	numin	2.88E-35	-0.16666
Nvex	ranincd	4.59E-17	-0.11462
Nvex	crpkm	1.17E-41	-0.18142
Nvex	posg	2.50E-07	-0.07286
Nvex	lenin	1.69E-10	0.08865
Nvex	lenex1	1.87E-03	0.04685
Nvex	lenex2	8.15E-04	0.04984
Nvex	ralinex1	1.91E-08	0.07878
Nvex	ralinex2	3.08E-07	0.07236
Nvex	gcin	1.17E-10	0.08937
Nvex	gcex1	4.04E-03	-0.04387
Nvex	gcex2	5.12E-04	-0.05144
Nvex	difginex1	2.42E-10	0.08793
Nvex	difginex2	1.38E-13	0.10169
Nvex	ss5score	3.58E-07	-0.07200
Nvex	ss3score	1.88E-08	-0.07882
Aipt	lengene	6.78E-13	-0.11049
Aipt	lencds	2.40E-08	-0.08743
Aipt	lenints	3.53E-17	-0.12847
Aipt	ralincd	5.07E-07	-0.07947
Aipt	numin	8.18E-18	-0.13093
Aipt	ranincd	3.23E-16	-0.12466
Aipt	crpkm	3.01E-30	-0.17209
Aipt	posg	8.35E-09	-0.09002
Aipt	lenin	1.46E-10	0.09934
Aipt	lenex1	8.83E-03	0.04540
Aipt	lenex2	3.06E-03	0.05022
Aipt	ralinex1	7.56E-06	0.07168
Aipt	ralinex2	3.41E-05	0.06695
Aipt	gcin	1.39E-04	0.06220
Aipt	gcex1	2.61E-05	-0.06780
Aipt	gcex2	NA	NA
Aipt	difginex1	2.05E-05	0.06857
Aipt	difginex2	6.14E-03	0.04711
Aipt	ss5score	3.73E-03	-0.04935
Aipt	ss3score	2.99E-04	-0.05946
Mlei	lengene	4.71E-04	-0.16905
Mlei	lencds	NA	NA
Mlei	lenints	2.59E-05	-0.19848
Mlei	ralincd	4.88E-04	-0.16865
Mlei	numin	5.37E-04	-0.16758
Mlei	ranincd	8.59E-03	-0.13321
Mlei	crpkm	NA	NA
Mlei	posg	NA	NA
Mlei	lenin	NA	NA
Mlei	lenex1	NA	NA
Mlei	lenex2	NA	NA
Mlei	ralinex1	NA	NA
Mlei	ralinex2	NA	NA
Mlei	gcin	NA	NA
Mlei	gcex1	3.60E-03	-0.14488
Mlei	gcex2	NA	NA
Mlei	difginex1	NA	NA
Mlei	difginex2	NA	NA
Mlei	ss5score	2.21E-05	-0.19996
Mlei	ss3score	NA	NA
Aque	lengene	9.84E-92	-0.20076
Aque	lencds	1.26E-88	-0.19731
Aque	lenints	4.79E-73	-0.17897
Aque	ralincd	1.29E-14	-0.07843
Aque	numin	6.27E-72	-0.17758
Aque	ranincd	2.21E-07	0.05429
Aque	crpkm	8.89E-04	-0.03676
Aque	posg	2.13E-38	-0.12917
Aque	lenin	NA	NA
Aque	lenex1	1.55E-10	-0.06591
Aque	lenex2	9.47E-05	-0.04221
Aque	ralinex1	6.09E-03	0.03133
Aque	ralinex2	NA	NA
Aque	gcin	1.27E-25	0.10501
Aque	gcex1	1.80E-07	-0.05466
Aque	gcex2	8.92E-04	-0.03675
Aque	difginex1	1.00E-27	0.10935
Aque	difginex2	9.48E-24	0.10098
Aque	ss5score	4.63E-33	-0.11967
Aque	ss3score	7.27E-27	-0.10759
Ocar	lengene	3.87E-16	-0.13182
Ocar	lencds	1.44E-16	-0.13364
Ocar	lenints	8.01E-09	-0.09554
Ocar	ralincd	NA	NA
Ocar	numin	8.70E-18	-0.13869
Ocar	ranincd	NA	NA
Ocar	crpkm	2.90E-07	-0.08585
Ocar	posg	1.64E-09	-0.09952
Ocar	lenin	6.22E-10	0.10188
Ocar	lenex1	9.81E-03	-0.04758
Ocar	lenex2	NA	NA
Ocar	ralinex1	4.74E-07	0.08444
Ocar	ralinex2	2.06E-05	0.07269
Ocar	gcin	4.15E-07	0.08482
Ocar	gcex1	NA	NA
Ocar	gcex2	NA	NA
Ocar	difginex1	1.53E-05	0.07368
Ocar	difginex2	1.52E-06	0.08099
Ocar	ss5score	1.79E-15	-0.12894
Ocar	ss3score	1.49E-03	-0.05645

species	Architectural feature	oKS p-value	oKS D statistic
Scil	lengene	6.34E-23	-0.20957
Scil	lencds	9.68E-11	-0.14076
Scil	lenints	1.36E-25	-0.22181
Scil	ralincd	5.37E-19	-0.19012
Scil	numin	1.65E-27	-0.23019
Scil	ranincd	3.49E-25	-0.21997
Scil	crpkm	1.73E-04	-0.08627
Scil	posg	4.96E-16	-0.17401
Scil	lenin	NA	NA
Scil	lenex1	2.65E-08	0.12244
Scil	lenex2	2.60E-06	0.10512
Scil	ralinex1	2.64E-04	-0.08415
Scil	ralinex2	4.27E-03	-0.06847
Scil	gcin	NA	NA
Scil	gcex1	NA	NA
Scil	gcex2	NA	NA
Scil	difginex1	NA	NA
Scil	difginex2	NA	NA
Scil	ss5score	1.67E-12	-0.15265
Scil	ss3score	5.63E-08	-0.11976
Sros	lengene	1.35E-06	-0.23159
Sros	lencds	1.27E-04	-0.18869
Sros	lenints	3.42E-06	-0.22349
Sros	ralincd	NA	NA
Sros	numin	5.57E-05	-0.19718
Sros	ranincd	NA	NA
Sros	crpkm	NA	NA
Sros	posg	NA	NA
Sros	lenin	NA	NA
Sros	lenex1	NA	NA
Sros	lenex2	NA	NA
Sros	ralinex1	NA	NA
Sros	ralinex2	NA	NA
Sros	gcin	NA	NA
Sros	gcex1	NA	NA
Sros	gcex2	NA	NA
Sros	difginex1	7.46E-03	0.13944
Sros	difginex2	NA	NA
Sros	ss5score	NA	NA
Sros	ss3score	NA	NA
Cowc	lengene	7.95E-12	-0.13232
Cowc	lencds	1.39E-06	-0.09611
Cowc	lenints	5.01E-10	-0.12111
Cowc	ralincd	NA	NA
Cowc	numin	4.36E-06	-0.09195
Cowc	ranincd	4.70E-03	0.06059
Cowc	crpkm	3.73E-07	-0.10070
Cowc	posg	3.59E-42	-0.25567
Cowc	lenin	8.03E-18	0.16421
Cowc	lenex1	1.00E-04	-0.07942
Cowc	lenex2	3.87E-03	-0.06168
Cowc	ralinex1	9.10E-15	0.14882
Cowc	ralinex2	3.16E-07	0.10125
Cowc	gcin	1.07E-04	0.07912
Cowc	gcex1	5.19E-04	0.07198
Cowc	gcex2	NA	NA
Cowc	difginex1	NA	NA
Cowc	difginex2	NA	NA
Cowc	ss5score	3.45E-16	-0.15617
Cowc	ss3score	1.22E-04	-0.07858
Cfra	lengene	6.47E-58	-0.18610
Cfra	lencds	1.51E-57	-0.18550
Cfra	lenints	1.98E-53	-0.17865
Cfra	ralincd	NA	NA
Cfra	numin	1.36E-56	-0.18394
Cfra	ranincd	2.43E-05	0.05286
Cfra	crpkm	3.09E-60	-0.18984
Cfra	posg	4.13E-08	-0.06687
Cfra	lenin	NA	NA
Cfra	lenex1	7.53E-11	-0.07830
Cfra	lenex2	1.77E-08	-0.06852
Cfra	ralinex1	1.70E-07	0.06403
Cfra	ralinex2	8.28E-05	0.04972
Cfra	gcin	7.83E-14	0.08909
Cfra	gcex1	9.18E-03	-0.03512
Cfra	gcex2	4.22E-06	-0.05705
Cfra	difginex1	2.75E-11	0.07997
Cfra	difginex2	1.10E-15	0.09517
Cfra	ss5score	9.97E-29	-0.13022
Cfra	ss3score	2.09E-10	-0.07657
Sar3	lengene	2.18E-18	-0.22347
Sar3	lencds	1.81E-06	-0.12743
Sar3	lenints	1.47E-21	-0.24271
Sar3	ralincd	1.86E-20	-0.23620
Sar3	numin	1.65E-07	-0.13849
Sar3	ranincd	8.96E-06	-0.11947
Sar3	crpkm	5.38E-24	-0.25651
Sar3	posg	4.27E-07	-0.13421
Sar3	lenin	1.03E-10	-0.16805
Sar3	lenex1	NA	NA
Sar3	lenex2	1.69E-03	0.08853
Sar3	ralinex1	9.13E-08	-0.14109
Sar3	ralinex2	1.22E-10	-0.16743
Sar3	gcin	2.31E-12	0.18140
Sar3	gcex1	NA	NA
Sar3	gcex2	NA	NA
Sar3	difginex1	2.70E-13	0.18852
Sar3	difginex2	4.23E-12	0.17934
Sar3	ss5score	4.87E-07	-0.13361
Sar3	ss3score	1.33E-06	-0.12891
Sarc4	lengene	7.65E-83	-0.32785
Sarc4	lencds	3.08E-24	-0.17543
Sarc4	lenints	5.33E-83	-0.32817
Sarc4	ralincd	2.51E-41	-0.23053
Sarc4	numin	5.68E-63	-0.28544
Sarc4	ranincd	1.41E-27	-0.18748
Sarc4	crpkm	9.47E-31	-0.19824
Sarc4	posg	4.39E-17	-0.14633
Sarc4	lenin	4.67E-16	-0.14166
Sarc4	lenex1	NA	NA
Sarc4	lenex2	7.58E-12	0.12065
Sarc4	ralinex1	1.29E-10	-0.11378
Sarc4	ralinex2	1.46E-23	-0.17289
Sarc4	gcin	9.06E-10	0.10880
Sarc4	gcex1	2.10E-03	-0.05920
Sarc4	gcex2	7.11E-03	-0.05303
Sarc4	difginex1	1.19E-10	0.11398
Sarc4	difginex2	5.30E-14	0.13183
Sarc4	ss5score	5.17E-05	-0.07491
Sarc4	ss3score	1.25E-04	-0.07147
Nk52	lengene	NA	NA
Nk52	lencds	NA	NA
Nk52	lenints	2.47E-03	0.07392
Nk52	ralincd	NA	NA
Nk52	numin	6.83E-05	0.09345
Nk52	ranincd	6.70E-03	0.06751
Nk52	crpkm	9.99E-25	-0.22430
Nk52	posg	6.91E-12	0.15296
Nk52	lenin	NA	NA
Nk52	lenex1	NA	NA
Nk52	lenex2	6.64E-07	-0.11380
Nk52	ralinex1	NA	NA
Nk52	ralinex2	1.31E-05	0.10118
Nk52	gcin	NA	NA
Nk52	gcex1	NA	NA
Nk52	gcex2	6.14E-03	-0.06810
Nk52	difginex1	NA	NA
Nk52	difginex2	6.75E-04	0.08153
Nk52	ss5score	1.26E-04	-0.09040
Nk52	ss3score	1.12E-04	-0.09100

species	Architectural feature	oKS p-value	oKS D statistic
Ncrx	lengene	1.88E-04	-0.10521
Ncrx	lencds	5.17E-05	-0.11286
Ncrx	lenints	NA	NA
Ncrx	ralincd	1.19E-03	0.09319
Ncrx	numin	7.21E-03	-0.07978
Ncrx	ranincd	NA	NA
Ncrx	crpkm	1.85E-21	-0.24820
Ncrx	posg	NA	NA
Ncrx	lenin	5.82E-10	0.16565
Ncrx	lenex1	6.10E-03	-0.08112
Ncrx	lenex2	NA	NA
Ncrx	ralinex1	1.10E-05	0.12140
Ncrx	ralinex2	9.05E-03	0.07792
Ncrx	gcin	6.30E-04	0.09752
Ncrx	gcex1	NA	NA
Ncrx	gcex2	9.12E-03	0.07786
Ncrx	difginex1	2.49E-03	0.08795
Ncrx	difginex2	NA	NA
Ncrx	ss5score	5.99E-05	-0.11201
Ncrx	ss3score	NA	NA
Spom	lengene	NA	NA
Spom	lencds	NA	NA
Spom	lenints	NA	NA
Spom	ralincd	NA	NA
Spom	numin	NA	NA
Spom	ranincd	NA	NA
Spom	crpkm	1.03E-26	-0.25343
Spom	posg	NA	NA
Spom	lenin	NA	NA
Spom	lenex1	NA	NA
Spom	lenex2	NA	NA
Spom	ralinex1	NA	NA
Spom	ralinex2	NA	NA
Spom	gcin	1.16E-05	0.11044
Spom	gcex1	NA	NA
Spom	gcex2	2.83E-05	-0.10602
Spom	difginex1	3.17E-08	0.13614
Spom	difginex2	1.20E-08	0.13993
Spom	ss5score	2.04E-05	-0.10766
Spom	ss3score	1.19E-05	-0.11031
Aory	lengene	3.68E-05	-0.10969
Aory	lencds	1.51E-04	-0.10182
Aory	lenints	6.03E-03	-0.07760
Aory	ralincd	NA	NA
Aory	numin	1.82E-03	-0.08624
Aory	ranincd	NA	NA
Aory	crpkm	3.29E-28	-0.27308
Aory	posg	1.36E-03	-0.08819
Aory	lenin	3.24E-05	0.11038
Aory	lenex1	NA	NA
Aory	lenex2	4.61E-05	0.10848
Aory	ralinex1	2.63E-03	0.08366
Aory	ralinex2	NA	NA
Aory	gcin	1.01E-07	0.13777
Aory	gcex1	NA	NA
Aory	gcex2	NA	NA
Aory	difginex1	1.12E-06	0.12709
Aory	difginex2	3.04E-05	0.11071
Aory	ss5score	2.99E-03	-0.08277
Aory	ss3score	3.98E-03	-0.08070
Cneo	lengene	1.01E-07	-0.13895
Cneo	lencds	1.07E-07	-0.13869
Cneo	lenints	1.66E-03	-0.08759
Cneo	ralincd	2.13E-04	0.10067
Cneo	numin	4.36E-05	-0.10970
Cneo	ranincd	NA	NA
Cneo	crpkm	9.59E-04	0.09127
Cneo	posg	4.56E-09	-0.15172
Cneo	lenin	2.48E-15	0.20078
Cneo	lenex1	4.29E-04	-0.09640
Cneo	lenex2	NA	NA
Cneo	ralinex1	1.06E-05	0.11716
Cneo	ralinex2	NA	NA
Cneo	gcin	NA	NA
Cneo	gcex1	NA	NA
Cneo	gcex2	NA	NA
Cneo	difginex1	4.72E-03	0.08012
Cneo	difginex2	NA	NA
Cneo	ss5score	1.07E-05	-0.11714
Cneo	ss3score	NA	NA
Umay	lengene	1.26E-06	0.20672
Umay	lencds	1.06E-06	0.20801
Umay	lenints	NA	NA
Umay	ralincd	9.36E-05	-0.17083
Umay	numin	NA	NA
Umay	ranincd	1.25E-03	-0.14503
Umay	crpkm	2.07E-29	-0.45584
Umay	posg	5.46E-08	0.22937
Umay	lenin	NA	NA
Umay	lenex1	2.11E-03	0.13924
Umay	lenex2	NA	NA
Umay	ralinex1	1.00E-03	-0.14738
Umay	ralinex2	NA	NA
Umay	gcin	NA	NA
Umay	gcex1	NA	NA
Umay	gcex2	NA	NA
Umay	difginex1	NA	NA
Umay	difginex2	NA	NA
Umay	ss5score	NA	NA
Umay	ss3score	1.05E-03	-0.14689
Tmel	lengene	5.10E-05	-0.06412
Tmel	lencds	9.95E-08	-0.08190
Tmel	lenints	5.53E-06	-0.07097
Tmel	ralincd	6.84E-05	0.06317
Tmel	numin	1.75E-09	-0.09160
Tmel	ranincd	NA	NA
Tmel	crpkm	1.83E-12	-0.10605
Tmel	posg	1.86E-14	-0.11470
Tmel	lenin	2.36E-40	0.19485
Tmel	lenex1	2.70E-04	-0.05846
Tmel	lenex2	NA	NA
Tmel	ralinex1	1.36E-14	0.11526
Tmel	ralinex2	2.80E-07	0.07923
Tmel	gcin	1.31E-28	0.16344
Tmel	gcex1	5.25E-13	0.10846
Tmel	gcex2	7.90E-11	0.09838
Tmel	difginex1	6.07E-08	0.08315
Tmel	difginex2	1.77E-10	0.09665
Tmel	ss5score	3.03E-21	-0.14021
Tmel	ss3score	7.16E-20	-0.13543
Rory	lengene	5.64E-10	-0.11157
Rory	lencds	9.98E-09	-0.10377
Rory	lenints	1.99E-05	-0.07955
Rory	ralincd	NA	NA
Rory	numin	7.97E-09	-0.10440
Rory	ranincd	NA	NA
Rory	crpkm	1.29E-08	-0.10305
Rory	posg	2.01E-09	-0.10819
Rory	lenin	2.09E-05	-0.07937
Rory	lenex1	5.91E-03	-0.05477
Rory	lenex2	NA	NA
Rory	ralinex1	2.71E-03	0.05878
Rory	ralinex2	NA	NA
Rory	gcin	9.70E-12	0.12175
Rory	gcex1	NA	NA
Rory	gcex2	1.48E-03	-0.06170
Rory	difginex1	1.43E-09	0.10911
Rory	difginex2	2.93E-13	0.12988
Rory	ss5score	6.96E-07	-0.09104
Rory	ss3score	1.35E-03	-0.06215

species	Architectural feature	oKS p-value	oKS D statistic
Amac	lengene	1.39E-07	-0.18391
Amac	lencds	3.19E-07	-0.17899
Amac	lenints	4.48E-04	-0.12852
Amac	ralincd	NA	NA
Amac	numin	3.15E-06	-0.16473
Amac	ranincd	NA	NA
Amac	crpkm	NA	NA
Amac	posg	4.18E-05	-0.14696
Amac	lenin	4.78E-04	0.12796
Amac	lenex1	NA	NA
Amac	lenex2	NA	NA
Amac	ralinex1	5.81E-03	0.10501
Amac	ralinex2	NA	NA
Amac	gcin	4.19E-04	0.12907
Amac	gcex1	NA	NA
Amac	gcex2	NA	NA
Amac	difginex1	NA	NA
Amac	difginex2	NA	NA
Amac	ss5score	3.83E-15	-0.26665
Amac	ss3score	NA	NA
Spun	lengene	4.04E-05	-0.10058
Spun	lencds	2.04E-05	-0.10393
Spun	lenints	1.05E-03	-0.08280
Spun	ralincd	NA	NA
Spun	numin	4.61E-03	-0.07334
Spun	ranincd	NA	NA
Spun	crpkm	1.78E-11	-0.15732
Spun	posg	NA	NA
Spun	lenin	2.73E-06	0.11318
Spun	lenex1	5.87E-03	-0.07168
Spun	lenex2	NA	NA
Spun	ralinex1	4.24E-05	0.10034
Spun	ralinex2	NA	NA
Spun	gcin	NA	NA
Spun	gcex1	NA	NA
Spun	gcex2	NA	NA
Spun	difginex1	NA	NA
Spun	difginex2	NA	NA
Spun	ss5score	4.50E-16	-0.18798
Spun	ss3score	NA	NA
Rirr	lengene	1.07E-09	-0.11058
Rirr	lencds	4.17E-12	-0.12454
Rirr	lenints	1.17E-06	-0.08991
Rirr	ralincd	NA	NA
Rirr	numin	5.85E-08	-0.09929
Rirr	ranincd	NA	NA
Rirr	crpkm	2.70E-15	-0.14091
Rirr	posg	1.54E-06	-0.08901
Rirr	lenin	NA	NA
Rirr	lenex1	NA	NA
Rirr	lenex2	NA	NA
Rirr	ralinex1	1.89E-03	0.06094
Rirr	ralinex2	4.49E-03	0.05657
Rirr	gcin	2.43E-27	0.19045
Rirr	gcex1	NA	NA
Rirr	gcex2	NA	NA
Rirr	difginex1	3.73E-22	0.17090
Rirr	difginex2	8.03E-18	0.15264
Rirr	ss5score	1.55E-10	-0.11563
Rirr	ss3score	3.66E-07	-0.09366
Rirr2	lengene	NA	NA
Rirr2	lencds	8.04E-04	-0.29877
Rirr2	lenints	NA	NA
Rirr2	ralincd	NA	NA
Rirr2	numin	NA	NA
Rirr2	ranincd	NA	NA
Rirr2	crpkm	1.30E-03	-0.28846
Rirr2	posg	2.56E-04	-0.32188
Rirr2	lenin	NA	NA
Rirr2	lenex1	NA	NA
Rirr2	lenex2	NA	NA
Rirr2	ralinex1	NA	NA
Rirr2	ralinex2	NA	NA
Rirr2	gcin	4.98E-04	0.30864
Rirr2	gcex1	NA	NA
Rirr2	gcex2	NA	NA
Rirr2	difginex1	6.17E-03	0.25247
Rirr2	difginex2	1.73E-04	0.32937
Rirr2	ss5score	5.60E-03	-0.25487
Rirr2	ss3score	NA	NA
Ccor	lengene	NA	NA
Ccor	lencds	NA	NA
Ccor	lenints	NA	NA
Ccor	ralincd	NA	NA
Ccor	numin	3.21E-03	-0.27987
Ccor	ranincd	NA	NA
Ccor	crpkm	6.41E-03	-0.26246
Ccor	posg	NA	NA
Ccor	lenin	NA	NA
Ccor	lenex1	NA	NA
Ccor	lenex2	NA	NA
Ccor	ralinex1	NA	NA
Ccor	ralinex2	NA	NA
Ccor	gcin	2.82E-07	0.45353
Ccor	gcex1	NA	NA
Ccor	gcex2	NA	NA
Ccor	difginex1	1.04E-03	0.30612
Ccor	difginex2	1.67E-03	0.29541
Ccor	ss5score	NA	NA
Ccor	ss3score	NA	NA
Gpro	lengene	8.49E-18	-0.20421
Gpro	lencds	3.20E-13	-0.17471
Gpro	lenints	1.27E-17	-0.20316
Gpro	ralincd	4.36E-04	-0.09060
Gpro	numin	1.20E-12	-0.17064
Gpro	ranincd	5.54E-03	-0.07425
Gpro	crpkm	1.99E-22	-0.23024
Gpro	posg	1.05E-09	-0.14810
Gpro	lenin	7.36E-04	0.08748
Gpro	lenex1	NA	NA
Gpro	lenex2	NA	NA
Gpro	ralinex1	NA	NA
Gpro	ralinex2	NA	NA
Gpro	gcin	8.17E-07	0.12195
Gpro	gcex1	NA	NA
Gpro	gcex2	NA	NA
Gpro	difginex1	3.56E-04	0.09178
Gpro	difginex2	6.44E-05	0.10118
Gpro	ss5score	2.60E-11	-0.16080
Gpro	ss3score	1.99E-03	-0.08123
Falb	lengene	NA	NA
Falb	lencds	NA	NA
Falb	lenints	1.28E-03	-0.24302
Falb	ralincd	NA	NA
Falb	numin	3.38E-03	-0.22459
Falb	ranincd	NA	NA
Falb	crpkm	NA	NA
Falb	posg	NA	NA
Falb	lenin	NA	NA
Falb	lenex1	NA	NA
Falb	lenex2	NA	NA
Falb	ralinex1	NA	NA
Falb	ralinex2	NA	NA
Falb	gcin	NA	NA
Falb	gcex1	NA	NA
Falb	gcex2	NA	NA
Falb	difginex1	NA	NA
Falb	difginex2	2.06E-03	-0.23411
Falb	ss5score	NA	NA
Falb	ss3score	4.08E-03	-0.22081

species	Architectural feature	oKS p-value	oKS D statistic
Ddis2	lengene	8.03E-04	-0.33497
Ddis2	lencds	1.41E-04	-0.37361
Ddis2	lenints	NA	NA
Ddis2	ralincd	1.94E-04	0.36685
Ddis2	numin	NA	NA
Ddis2	ranincd	8.78E-04	0.33286
Ddis2	crpkm	NA	NA
Ddis2	posg	NA	NA
Ddis2	lenin	2.74E-03	0.30478
Ddis2	lenex1	NA	NA
Ddis2	lenex2	7.02E-04	-0.33811
Ddis2	ralinex1	1.35E-03	0.32254
Ddis2	ralinex2	8.96E-05	0.38306
Ddis2	gcin	6.23E-06	0.43439
Ddis2	gcex1	NA	NA
Ddis2	gcex2	NA	NA
Ddis2	difginex1	3.41E-04	0.35453
Ddis2	difginex2	9.44E-05	0.38197
Ddis2	ss5score	NA	NA
Ddis2	ss3score	1.16E-03	-0.32617
Ppal	lengene	NA	NA
Ppal	lencds	NA	NA
Ppal	lenints	NA	NA
Ppal	ralincd	7.94E-04	0.19437
Ppal	numin	NA	NA
Ppal	ranincd	NA	NA
Ppal	crpkm	6.23E-05	-0.22638
Ppal	posg	NA	NA
Ppal	lenin	3.62E-10	0.33920
Ppal	lenex1	1.22E-03	-0.18845
Ppal	lenex2	NA	NA
Ppal	ralinex1	5.51E-06	0.25316
Ppal	ralinex2	7.21E-06	0.25033
Ppal	gcin	1.48E-04	0.21602
Ppal	gcex1	2.98E-03	-0.17543
Ppal	gcex2	NA	NA
Ppal	difginex1	2.91E-04	0.20757
Ppal	difginex2	5.98E-03	0.16462
Ppal	ss5score	3.05E-03	-0.17511
Ppal	ss3score	2.53E-03	-0.17789
Acas	lengene	2.32E-06	-0.53129
Acas	lencds	3.60E-05	-0.47183
Acas	lenints	5.24E-07	-0.56098
Acas	ralincd	NA	NA
Acas	numin	2.83E-06	-0.52723
Acas	ranincd	NA	NA
Acas	crpkm	NA	NA
Acas	posg	1.09E-04	-0.44568
Acas	lenin	NA	NA
Acas	lenex1	NA	NA
Acas	lenex2	NA	NA
Acas	ralinex1	NA	NA
Acas	ralinex2	NA	NA
Acas	gcin	NA	NA
Acas	gcex1	NA	NA
Acas	gcex2	NA	NA
Acas	difginex1	NA	NA
Acas	difginex2	NA	NA
Acas	ss5score	NA	NA
Acas	ss3score	NA	NA
Atha	lengene	8.59E-78	-0.16353
Atha	lencds	2.11E-65	-0.14981
Atha	lenints	7.04E-87	-0.17290
Atha	ralincd	5.29E-24	-0.08987
Atha	numin	3.19E-95	-0.18108
Atha	ranincd	6.53E-38	-0.11359
Atha	crpkm	NA	NA
Atha	posg	1.16E-32	-0.10527
Atha	lenin	NA	NA
Atha	lenex1	6.16E-07	0.04642
Atha	lenex2	2.15E-13	0.06630
Atha	ralinex1	NA	NA
Atha	ralinex2	NA	NA
Atha	gcin	4.20E-04	0.03423
Atha	gcex1	3.92E-05	0.03911
Atha	gcex2	9.98E-09	0.05269
Atha	difginex1	NA	NA
Atha	difginex2	3.63E-06	-0.04345
Atha	ss5score	8.47E-27	-0.09512
Atha	ss3score	4.75E-14	-0.06799
Atha3	lengene	3.83E-28	-0.19623
Atha3	lencds	1.34E-20	-0.16706
Atha3	lenints	9.15E-32	-0.20878
Atha3	ralincd	1.83E-09	-0.11078
Atha3	numin	1.51E-30	-0.20465
Atha3	ranincd	1.16E-14	-0.13989
Atha3	crpkm	1.80E-03	0.06208
Atha3	posg	1.69E-10	-0.11714
Atha3	lenin	8.17E-03	-0.05415
Atha3	lenex1	8.10E-03	0.05419
Atha3	lenex2	3.99E-04	0.06909
Atha3	ralinex1	5.34E-03	-0.05650
Atha3	ralinex2	5.35E-03	-0.05648
Atha3	gcin	7.25E-04	0.06641
Atha3	gcex1	2.04E-03	0.06146
Atha3	gcex2	7.89E-03	0.05434
Atha3	difginex1	NA	NA
Atha3	difginex2	NA	NA
Atha3	ss5score	1.14E-18	-0.15874
Atha3	ss3score	2.59E-05	-0.08025
Vvin	lengene	1.46E-99	-0.23425
Vvin	lencds	1.30E-31	-0.13095
Vvin	lenints	1.92E-97	-0.23172
Vvin	ralincd	9.08E-75	-0.20275
Vvin	numin	1.34E-57	-0.17769
Vvin	ranincd	4.10E-25	-0.11636
Vvin	crpkm	2.62E-26	-0.11918
Vvin	posg	7.95E-32	-0.13140
Vvin	lenin	5.65E-23	-0.11114
Vvin	lenex1	5.06E-04	0.04277
Vvin	lenex2	9.19E-16	0.09137
Vvin	ralinex1	7.42E-16	-0.09165
Vvin	ralinex2	3.93E-22	-0.10902
Vvin	gcin	3.55E-11	0.07617
Vvin	gcex1	NA	NA
Vvin	gcex2	7.14E-05	0.04798
Vvin	difginex1	2.12E-03	0.03853
Vvin	difginex2	NA	NA
Vvin	ss5score	9.57E-25	-0.11548
Vvin	ss3score	7.27E-26	-0.11814
Mgut	lengene	2.73E-28	-0.17306
Mgut	lencds	3.64E-12	-0.11148
Mgut	lenints	7.31E-36	-0.19539
Mgut	ralincd	2.23E-25	-0.16366
Mgut	numin	9.92E-34	-0.18936
Mgut	ranincd	2.47E-28	-0.17319
Mgut	crpkm	6.99E-33	-0.18691
Mgut	posg	2.46E-19	-0.14219
Mgut	lenin	2.93E-03	0.05246
Mgut	lenex1	2.84E-08	0.09055
Mgut	lenex2	3.11E-15	0.12555
Mgut	ralinex1	1.48E-06	-0.07959
Mgut	ralinex2	1.74E-09	-0.09755
Mgut	gcin	2.99E-14	0.12122
Mgut	gcex1	8.61E-08	-0.08761
Mgut	gcex2	1.63E-05	-0.07212
Mgut	difginex1	2.75E-20	0.14578
Mgut	difginex2	8.57E-14	0.11915
Mgut	ss5score	9.64E-16	-0.12773
Mgut	ss3score	1.65E-10	-0.10310

species	Architectural feature	oKS p-value	oKS D statistic
Osat2	lengene	1.45E-17	-0.22490
Osat2	lencds	6.76E-08	-0.14675
Osat2	lenints	3.60E-21	-0.24781
Osat2	ralincd	2.07E-16	-0.21705
Osat2	numin	6.38E-21	-0.24629
Osat2	ranincd	1.12E-16	-0.21889
Osat2	crpkm	8.22E-12	-0.18247
Osat2	posg	1.79E-16	-0.21748
Osat2	lenin	NA	NA
Osat2	lenex1	3.58E-07	0.13914
Osat2	lenex2	1.56E-08	0.15312
Osat2	ralinex1	1.89E-03	-0.09045
Osat2	ralinex2	3.28E-04	-0.10231
Osat2	gcin	7.15E-18	0.22693
Osat2	gcex1	2.59E-20	0.24256
Osat2	gcex2	3.80E-20	0.24152
Osat2	difginex1	8.78E-07	-0.13488
Osat2	difginex2	2.30E-06	-0.13014
Osat2	ss5score	2.33E-04	-0.10446
Osat2	ss3score	3.64E-07	-0.13907
Ppat	lengene	5.91E-54	-0.15478
Ppat	lencds	1.82E-39	-0.13205
Ppat	lenints	1.63E-48	-0.14666
Ppat	ralincd	1.36E-06	-0.05139
Ppat	numin	6.32E-53	-0.15328
Ppat	ranincd	6.53E-10	-0.06430
Ppat	crpkm	4.66E-07	-0.05339
Ppat	posg	4.03E-35	-0.12442
Ppat	lenin	1.59E-11	0.06971
Ppat	lenex1	NA	NA
Ppat	lenex2	2.17E-05	0.04581
Ppat	ralinex1	6.62E-08	0.05684
Ppat	ralinex2	6.08E-08	0.05699
Ppat	gcin	6.94E-26	0.10641
Ppat	gcex1	4.33E-10	0.06492
Ppat	gcex2	2.87E-05	0.04521
Ppat	difginex1	9.31E-03	0.03024
Ppat	difginex2	1.01E-07	0.05611
Ppat	ss5score	3.41E-43	-0.13826
Ppat	ss3score	3.03E-39	-0.13167
Marpol	lengene	2.24E-19	-0.10259
Marpol	lencds	1.36E-20	-0.10589
Marpol	lenints	3.57E-21	-0.10742
Marpol	ralincd	6.05E-07	-0.05924
Marpol	numin	1.61E-35	-0.14012
Marpol	ranincd	4.50E-11	-0.07641
Marpol	crpkm	1.10E-62	-0.18699
Marpol	posg	1.17E-21	-0.10869
Marpol	lenin	9.01E-23	0.11154
Marpol	lenex1	1.15E-03	0.04072
Marpol	lenex2	3.96E-05	0.04984
Marpol	ralinex1	2.07E-13	0.08460
Marpol	ralinex2	1.72E-08	0.06620
Marpol	gcin	2.52E-58	0.18029
Marpol	gcex1	8.38E-07	0.05856
Marpol	gcex2	3.79E-06	0.05531
Marpol	difginex1	2.38E-16	0.09390
Marpol	difginex2	3.54E-30	0.12892
Marpol	ss5score	1.31E-57	-0.17916
Marpol	ss3score	2.26E-43	-0.15513
Smoe	lengene	1.55E-05	-0.14283
Smoe	lencds	7.57E-04	-0.11505
Smoe	lenints	8.56E-06	-0.14660
Smoe	ralincd	2.73E-03	-0.10427
Smoe	numin	3.34E-07	-0.16573
Smoe	ranincd	1.06E-03	-0.11231
Smoe	crpkm	8.78E-03	0.09340
Smoe	posg	1.84E-04	-0.12585
Smoe	lenin	NA	NA
Smoe	lenex1	NA	NA
Smoe	lenex2	NA	NA
Smoe	ralinex1	NA	NA
Smoe	ralinex2	NA	NA
Smoe	gcin	NA	NA
Smoe	gcex1	NA	NA
Smoe	gcex2	7.37E-03	0.09510
Smoe	difginex1	NA	NA
Smoe	difginex2	NA	NA
Smoe	ss5score	4.71E-06	-0.15031
Smoe	ss3score	NA	NA
Kfla	lengene	8.86E-35	-0.18349
Kfla	lencds	3.35E-23	-0.14907
Kfla	lenints	2.55E-28	-0.16517
Kfla	ralincd	NA	NA
Kfla	numin	8.37E-21	-0.14089
Kfla	ranincd	NA	NA
Kfla	crpkm	7.83E-09	-0.08952
Kfla	posg	1.93E-05	-0.06827
Kfla	lenin	6.16E-06	-0.07177
Kfla	lenex1	1.55E-08	-0.08788
Kfla	lenex2	NA	NA
Kfla	ralinex1	4.26E-06	0.07287
Kfla	ralinex2	NA	NA
Kfla	gcin	1.30E-04	0.06200
Kfla	gcex1	5.58E-07	-0.07863
Kfla	gcex2	4.37E-06	-0.07279
Kfla	difginex1	4.40E-10	0.09618
Kfla	difginex2	8.26E-11	0.09985
Kfla	ss5score	1.21E-10	-0.09902
Kfla	ss3score	1.13E-08	-0.08865
Vcar	lengene	6.76E-06	0.03677
Vcar	lencds	1.51E-43	0.10583
Vcar	lenints	2.82E-05	-0.03450
Vcar	ralincd	8.00E-65	-0.12948
Vcar	numin	NA	NA
Vcar	ranincd	2.13E-75	-0.13975
Vcar	crpkm	3.36E-04	-0.03014
Vcar	posg	1.65E-09	-0.04793
Vcar	lenin	NA	NA
Vcar	lenex1	NA	NA
Vcar	lenex2	5.54E-13	0.05662
Vcar	ralinex1	4.31E-15	-0.05913
Vcar	ralinex2	4.71E-24	-0.07811
Vcar	gcin	4.57E-38	-0.09882
Vcar	gcex1	9.99E-22	-0.07411
Vcar	gcex2	1.31E-09	-0.04820
Vcar	difginex1	NA	NA
Vcar	difginex2	1.11E-07	-0.04265
Vcar	ss5score	1.18E-25	-0.08074
Vcar	ss3score	1.38E-36	-0.09685
Crei	lengene	1.91E-11	-0.11316
Crei	lencds	1.09E-10	-0.10910
Crei	lenints	2.41E-10	-0.10719
Crei	ralincd	NA	NA
Crei	numin	4.02E-14	-0.12651
Crei	ranincd	8.83E-06	-0.07771
Crei	crpkm	2.98E-13	-0.12233
Crei	posg	8.91E-03	-0.04949
Crei	lenin	NA	NA
Crei	lenex1	8.45E-06	-0.07785
Crei	lenex2	3.92E-03	-0.05362
Crei	ralinex1	1.11E-06	0.08435
Crei	ralinex2	9.75E-04	0.05998
Crei	gcin	1.17E-20	-0.15432
Crei	gcex1	NA	NA
Crei	gcex2	7.25E-03	-0.05056
Crei	difginex1	1.33E-15	-0.13331
Crei	difginex2	7.15E-12	-0.11539
Crei	ss5score	3.64E-16	-0.13581
Crei	ss3score	2.07E-20	-0.15335

species	Architectural feature	oKS p-value	oKS D statistic
Mpus	lengene	NA	NA
Mpus	lencds	NA	NA
Mpus	lenints	NA	NA
Mpus	ralincd	4.11E-03	-0.09330
Mpus	numin	1.05E-05	0.13477
Mpus	ranincd	9.02E-05	0.12146
Mpus	crpkm	NA	NA
Mpus	posg	1.18E-03	0.10337
Mpus	lenin	1.34E-11	-0.19915
Mpus	lenex1	NA	NA
Mpus	lenex2	2.19E-03	-0.09851
Mpus	ralinex1	1.09E-04	-0.12025
Mpus	ralinex2	NA	NA
Mpus	gcin	2.76E-21	-0.27385
Mpus	gcex1	3.19E-16	-0.23775
Mpus	gcex2	3.99E-18	-0.25193
Mpus	difginex1	7.48E-04	-0.10678
Mpus	difginex2	4.52E-07	-0.15213
Mpus	ss5score	1.80E-12	-0.20699
Mpus	ss3score	1.19E-17	-0.24847
Cpar	lengene	NA	NA
Cpar	lencds	NA	NA
Cpar	lenints	NA	NA
Cpar	ralincd	6.89E-03	-0.21868
Cpar	numin	NA	NA
Cpar	ranincd	NA	NA
Cpar	crpkm	NA	NA
Cpar	posg	NA	NA
Cpar	lenin	9.33E-03	-0.21192
Cpar	lenex1	NA	NA
Cpar	lenex2	NA	NA
Cpar	ralinex1	NA	NA
Cpar	ralinex2	NA	NA
Cpar	gcin	1.59E-06	-0.35814
Cpar	gcex1	1.20E-04	-0.29454
Cpar	gcex2	4.93E-05	-0.30870
Cpar	difginex1	2.14E-03	-0.24300
Cpar	difginex2	3.62E-04	-0.27592
Cpar	ss5score	4.62E-07	-0.37436
Cpar	ss3score	4.26E-05	-0.31096
Gthe	lengene	9.94E-36	-0.10064
Gthe	lencds	5.40E-24	-0.08205
Gthe	lenints	2.18E-50	-0.11987
Gthe	ralincd	4.89E-24	-0.08213
Gthe	numin	6.05E-29	-0.09036
Gthe	ranincd	2.36E-04	-0.03240
Gthe	crpkm	NA	NA
Gthe	posg	3.63E-05	0.03584
Gthe	lenin	5.96E-16	-0.06637
Gthe	lenex1	9.38E-07	-0.04176
Gthe	lenex2	NA	NA
Gthe	ralinex1	NA	NA
Gthe	ralinex2	NA	NA
Gthe	gcin	NA	NA
Gthe	gcex1	NA	NA
Gthe	gcex2	NA	NA
Gthe	difginex1	NA	NA
Gthe	difginex2	NA	NA
Gthe	ss5score	3.65E-26	-0.08579
Gthe	ss3score	NA	NA
GtheME	lengene	2.56E-14	-0.12501
GtheME	lencds	2.24E-11	-0.11066
GtheME	lenints	6.59E-18	-0.14055
GtheME	ralincd	8.69E-12	-0.11277
GtheME	numin	1.22E-07	-0.08917
GtheME	ranincd	1.77E-03	-0.05625
GtheME	crpkm	NA	NA
GtheME	posg	3.66E-04	0.06286
GtheME	lenin	2.46E-10	-0.10511
GtheME	lenex1	8.06E-04	-0.05964
GtheME	lenex2	NA	NA
GtheME	ralinex1	NA	NA
GtheME	ralinex2	NA	NA
GtheME	gcin	NA	NA
GtheME	gcex1	NA	NA
GtheME	gcex2	NA	NA
GtheME	difginex1	NA	NA
GtheME	difginex2	NA	NA
GtheME	ss5score	2.86E-07	-0.08674
GtheME	ss3score	NA	NA
Esil	lengene	8.18E-33	-0.15735
Esil	lencds	9.03E-25	-0.13621
Esil	lenints	2.96E-29	-0.14837
Esil	ralincd	5.38E-07	-0.06955
Esil	numin	4.23E-33	-0.15805
Esil	ranincd	1.68E-05	-0.06069
Esil	crpkm	7.39E-46	-0.18661
Esil	posg	5.06E-15	-0.10503
Esil	lenin	NA	NA
Esil	lenex1	NA	NA
Esil	lenex2	6.45E-03	0.04111
Esil	ralinex1	NA	NA
Esil	ralinex2	NA	NA
Esil	gcin	NA	NA
Esil	gcex1	2.13E-17	-0.11342
Esil	gcex2	1.27E-10	-0.08739
Esil	difginex1	7.35E-13	0.09676
Esil	difginex2	7.45E-11	0.08840
Esil	ss5score	3.58E-17	-0.11265
Esil	ss3score	2.99E-11	-0.09012
Pinf	lengene	8.45E-06	-0.09651
Pinf	lencds	2.68E-08	-0.11790
Pinf	lenints	3.12E-03	-0.06783
Pinf	ralincd	NA	NA
Pinf	numin	1.20E-03	-0.07324
Pinf	ranincd	NA	NA
Pinf	crpkm	1.04E-18	-0.18171
Pinf	posg	NA	NA
Pinf	lenin	NA	NA
Pinf	lenex1	NA	NA
Pinf	lenex2	NA	NA
Pinf	ralinex1	NA	NA
Pinf	ralinex2	NA	NA
Pinf	gcin	1.49E-08	0.11987
Pinf	gcex1	NA	NA
Pinf	gcex2	NA	NA
Pinf	difginex1	1.47E-07	0.11201
Pinf	difginex2	1.37E-04	0.08423
Pinf	ss5score	4.65E-09	-0.12369
Pinf	ss3score	1.90E-03	-0.07069
Aano	lengene	1.53E-03	0.12286
Aano	lencds	4.46E-03	0.11228
Aano	lenints	6.19E-03	0.10882
Aano	ralincd	NA	NA
Aano	numin	2.20E-06	0.17420
Aano	ranincd	NA	NA
Aano	crpkm	8.17E-05	-0.14807
Aano	posg	1.07E-11	0.24256
Aano	lenin	7.00E-10	-0.22159
Aano	lenex1	NA	NA
Aano	lenex2	NA	NA
Aano	ralinex1	2.53E-03	-0.11803
Aano	ralinex2	NA	NA
Aano	gcin	2.70E-15	-0.27953
Aano	gcex1	1.04E-11	-0.24269
Aano	gcex2	4.32E-12	-0.24688
Aano	difginex1	3.78E-09	-0.21255
Aano	difginex2	6.48E-09	-0.20957
Aano	ss5score	3.68E-04	-0.13573
Aano	ss3score	1.67E-14	-0.27182

species	Architectural feature	oKS p-value	oKS D statistic
Pfal2	lengene	NA	NA
Pfal2	lencds	6.26E-04	0.06891
Pfal2	lenints	4.80E-04	-0.07014
Pfal2	ralincd	4.80E-06	-0.08880
Pfal2	numin	1.21E-04	-0.07619
Pfal2	ranincd	2.03E-09	-0.11352
Pfal2	crpkm	1.05E-21	-0.17635
Pfal2	posg	1.69E-04	0.07477
Pfal2	lenin	5.98E-06	0.08799
Pfal2	lenex1	3.57E-10	0.11834
Pfal2	lenex2	1.45E-06	0.09302
Pfal2	ralinex1	9.27E-09	-0.10912
Pfal2	ralinex2	7.31E-03	-0.05627
Pfal2	gcin	5.42E-20	0.16899
Pfal2	gcex1	2.44E-07	-0.09900
Pfal2	gcex2	9.65E-03	0.05466
Pfal2	difginex1	9.20E-20	0.16798
Pfal2	difginex2	9.74E-04	0.06681
Pfal2	ss5score	NA	NA
Pfal2	ss3score	1.53E-10	-0.12062
Tthe	lengene	1.57E-08	-0.08597
Tthe	lencds	3.08E-26	-0.15543
Tthe	lenints	4.60E-05	0.06409
Tthe	ralincd	4.25E-25	0.15192
Tthe	numin	5.38E-11	-0.09862
Tthe	ranincd	4.50E-07	0.07753
Tthe	crpkm	7.39E-08	-0.08218
Tthe	posg	5.79E-12	-0.10316
Tthe	lenin	1.64E-40	0.19410
Tthe	lenex1	5.27E-15	-0.11628
Tthe	lenex2	1.27E-10	-0.09681
Tthe	ralinex1	1.71E-35	0.18145
Tthe	ralinex2	1.65E-27	0.15926
Tthe	gcin	4.75E-41	0.19541
Tthe	gcex1	1.43E-11	0.10134
Tthe	gcex2	3.53E-08	0.08401
Tthe	difginex1	3.85E-12	0.10397
Tthe	difginex2	1.76E-14	0.11413
Tthe	ss5score	NA	NA
Tthe	ss3score	1.76E-11	-0.10092
BnatMEA	lengene	1.50E-132	-0.14308
BnatMEA	lencds	7.83E-55	-0.09167
BnatMEA	lenints	6.16E-158	-0.15625
BnatMEA	ralincd	1.08E-147	-0.15108
BnatMEA	numin	3.67E-95	-0.12110
BnatMEA	ranincd	8.02E-59	-0.09499
BnatMEA	crpkm	2.86E-38	0.07636
BnatMEA	posg	3.13E-10	-0.03842
BnatMEA	lenin	NA	NA
BnatMEA	lenex1	NA	NA
BnatMEA	lenex2	8.59E-24	0.05985
BnatMEA	ralinex1	2.14E-33	-0.07123
BnatMEA	ralinex2	1.08E-45	-0.08357
BnatMEA	gcin	8.95E-33	-0.07055
BnatMEA	gcex1	1.17E-68	-0.10271
BnatMEA	gcex2	4.78E-80	-0.11099
BnatMEA	difginex1	5.79E-08	0.03352
BnatMEA	difginex2	5.24E-15	0.04709
BnatMEA	ss5score	5.85E-101	-0.12476
BnatMEA	ss3score	1.76E-21	-0.05677
Bnat	lengene	5.43E-13	-0.08667
Bnat	lencds	NA	NA
Bnat	lenints	8.34E-22	-0.11362
Bnat	ralincd	1.38E-48	-0.17121
Bnat	numin	3.22E-04	-0.04625
Bnat	ranincd	4.00E-13	-0.08714
Bnat	crpkm	6.08E-04	0.04438
Bnat	posg	7.59E-04	0.04371
Bnat	lenin	1.97E-22	-0.11530
Bnat	lenex1	3.38E-04	0.04611
Bnat	lenex2	3.42E-04	0.04607
Bnat	ralinex1	6.48E-15	-0.09322
Bnat	ralinex2	5.78E-19	-0.10569
Bnat	gcin	9.89E-28	-0.12861
Bnat	gcex1	6.55E-56	-0.18384
Bnat	gcex2	8.62E-58	-0.18695
Bnat	difginex1	9.40E-06	0.05549
Bnat	difginex2	8.10E-07	0.06108
Bnat	ss5score	1.29E-33	-0.14193
Bnat	ss3score	1.40E-03	-0.04181
Bnao	lengene	4.09E-82	-0.15904
Bnao	lencds	2.59E-09	-0.05166
Bnao	lenints	1.19E-122	-0.19466
Bnao	ralincd	1.86E-175	-0.23302
Bnao	numin	7.68E-65	-0.14116
Bnao	ranincd	2.88E-88	-0.16494
Bnao	crpkm	3.77E-17	0.07144
Bnao	posg	8.63E-08	-0.04685
Bnao	lenin	9.35E-98	-0.17365
Bnao	lenex1	8.68E-22	0.08090
Bnao	lenex2	4.07E-25	0.08706
Bnao	ralinex1	1.39E-85	-0.16239
Bnao	ralinex2	7.12E-95	-0.17105
Bnao	gcin	3.62E-21	-0.07970
Bnao	gcex1	2.03E-35	-0.10383
Bnao	gcex2	1.92E-49	-0.12304
Bnao	difginex1	6.79E-05	0.03599
Bnao	difginex2	4.87E-07	0.04429
Bnao	ss5score	4.71E-24	-0.08514
Bnao	ss3score	5.45E-12	-0.05916
Ehux	lengene	3.43E-03	0.10426
Ehux	lencds	1.52E-03	0.11148
Ehux	lenints	NA	NA
Ehux	ralincd	8.94E-03	-0.09507
Ehux	numin	NA	NA
Ehux	ranincd	3.34E-03	-0.10451
Ehux	crpkm	2.86E-10	0.20517
Ehux	posg	NA	NA
Ehux	lenin	NA	NA
Ehux	lenex1	NA	NA
Ehux	lenex2	NA	NA
Ehux	ralinex1	NA	NA
Ehux	ralinex2	NA	NA
Ehux	gcin	5.78E-20	-0.29131
Ehux	gcex1	1.59E-04	0.12946
Ehux	gcex2	NA	NA
Ehux	difginex1	3.60E-17	-0.26933
Ehux	difginex2	6.01E-20	-0.29118
Ehux	ss5score	4.31E-05	-0.13877
Ehux	ss3score	3.07E-11	-0.21534
Ngru	lengene	4.75E-05	-0.20680
Ngru	lencds	3.76E-04	-0.18406
Ngru	lenints	NA	NA
Ngru	ralincd	NA	NA
Ngru	numin	NA	NA
Ngru	ranincd	NA	NA
Ngru	crpkm	7.42E-07	-0.24623
Ngru	posg	4.33E-03	-0.15288
Ngru	lenin	NA	NA
Ngru	lenex1	NA	NA
Ngru	lenex2	9.05E-03	-0.14216
Ngru	ralinex1	NA	NA
Ngru	ralinex2	NA	NA
Ngru	gcin	7.61E-10	0.30032
Ngru	gcex1	NA	NA
Ngru	gcex2	NA	NA
Ngru	difginex1	1.07E-05	0.22169
Ngru	difginex2	8.22E-06	0.22427
Ngru	ss5score	NA	NA
Ngru	ss3score	2.50E-03	-0.16044

One-sided two-sample Kolmogorov-Smirnov test
Alternative hypothesis 1: the CDF of positive events lies **below** that of negative events
Alternative hypothesis 2: the CDF of positive events lies **above** that of negative events
If AH1 is TRUE, D>0
If AH2 is TRUE, D<0
If both are FALSE, D=NA
If both are TRUE, D=NA (marginal pvalues)

lengene	gene length
lencds	CDS length
lenints	total intron length
ralincd	ration intron length/CDS length
numin	number of introns in gene
ranincd	number of introns/CDS length
lenex	exon length
lenin1	5' intron length
lenin2	3' intron length
ralinex1	5' intron length/exon length
ralinex2	3' intron length/exon length
gcex	exon GC content
gcin1	5' intron GC content
gcin2	3' intron GC content
difginex1	Exon-5' intron GC content
difginex2	Exon-3' intron GC content
ss5score	5' SS heterogeneity score
ss3score	3' SS heterogeneity score
crpkm	expression level of transcript (cRPKM)
posg	position since start of gene

Fig S16. Species-specific comparisons of IR events with gene architectural features using dual Kolmogorov-Smirnov 2-sample tests with complementary alternative hypothesis (see Methods). D statistic encoded as positive/negative if either hypothesis is significant (p<0.01), NA if otherwise.

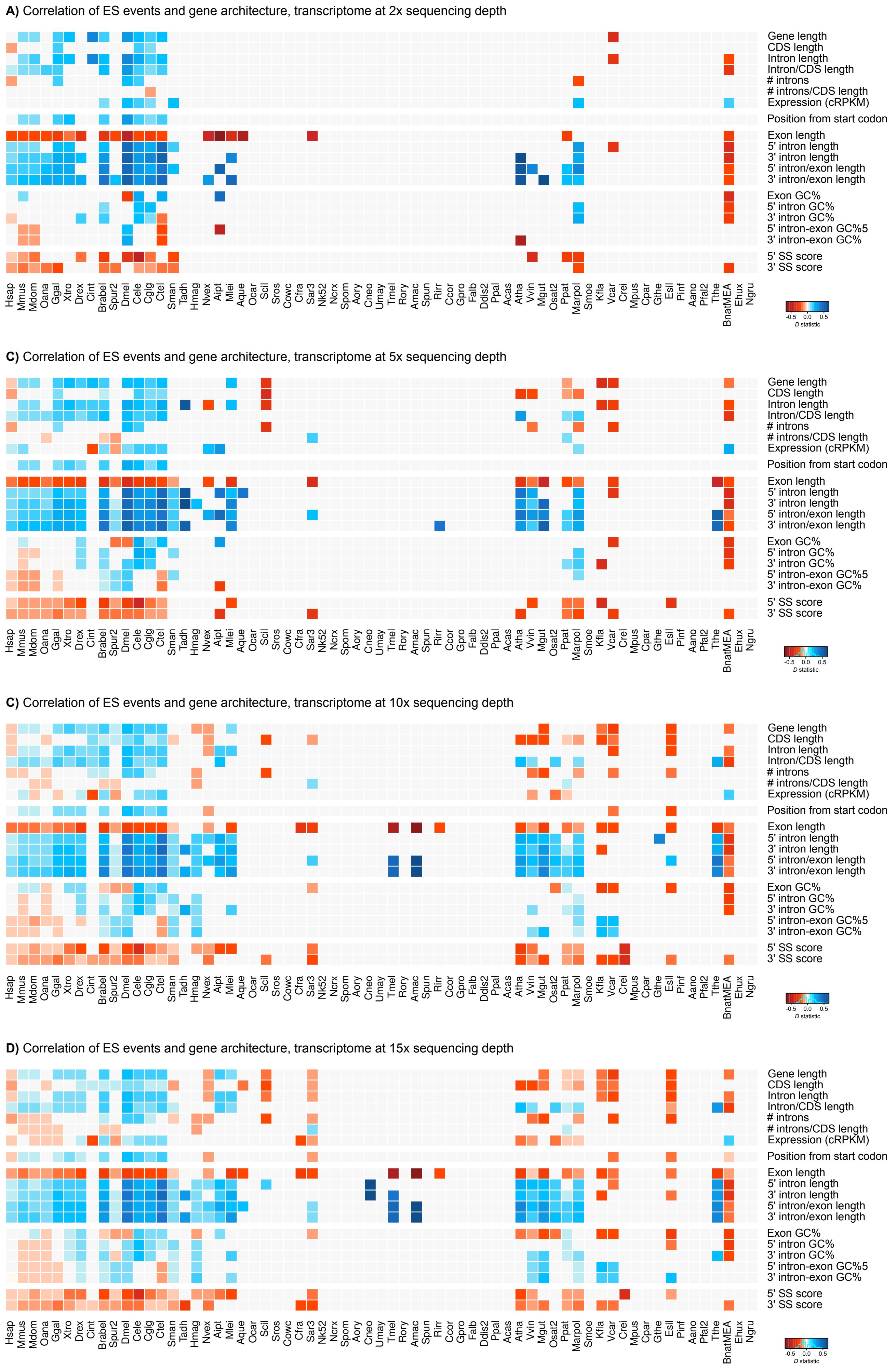

Fig S17. Relationship between gene structure and sequence composition and ES (as in Figure 4), downsampling each RNA-seq sample at 2x (A), 5x (B), 10x (C), and 15x (D).

Each heatmap represents the distance between distributions of gene architecture values for ES-positive and ES-negative exons, measured with the D statistic of the one-sided Kolmogorov-Smirnov two-sample test (significant if p<0.01; otherwise grey). D values are recorded as positive/negative (blue/red) according to two one-sided Kolmogorov-Smirnov tests with complementary alternative hypotheses: positive/negative D (blue/red) reflects a positive/negative relationship between ES and the given feature.

A) Correlation of IR events and gene architecture, transcriptome at 2x sequencing depth

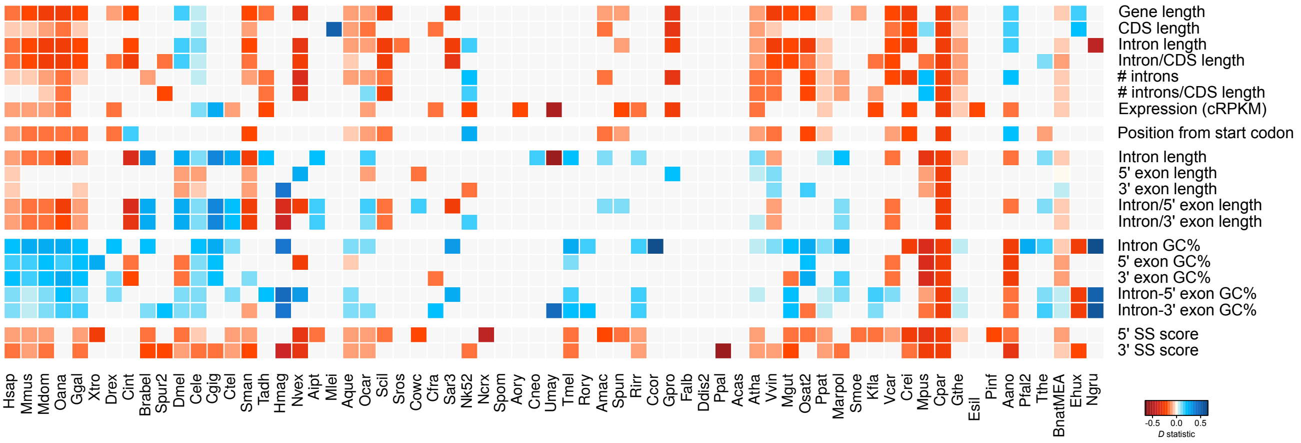

C) Correlation of IR events and gene architecture, transcriptome at 5x sequencing depth

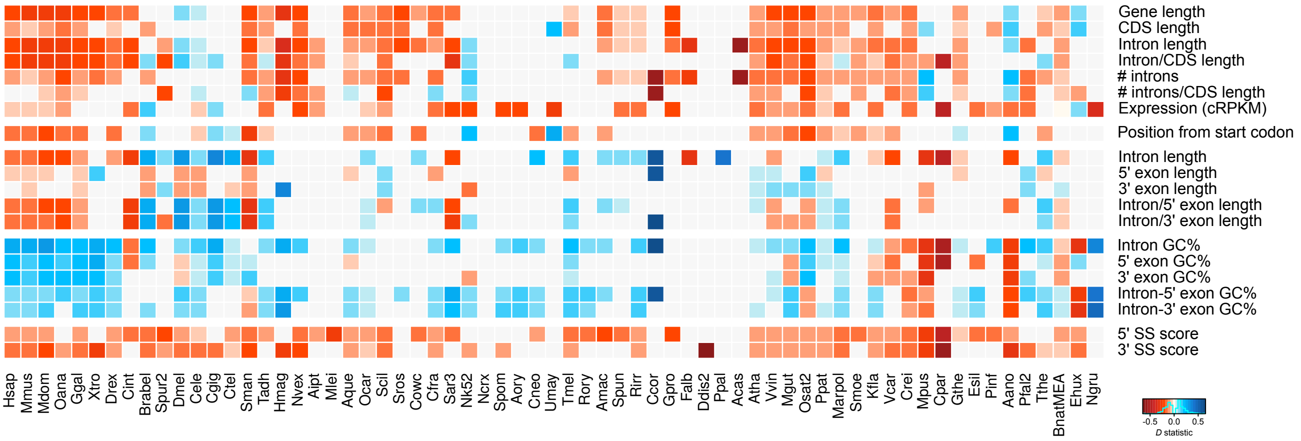

C) Correlation of IR events and gene architecture, transcriptome at 10x sequencing depth

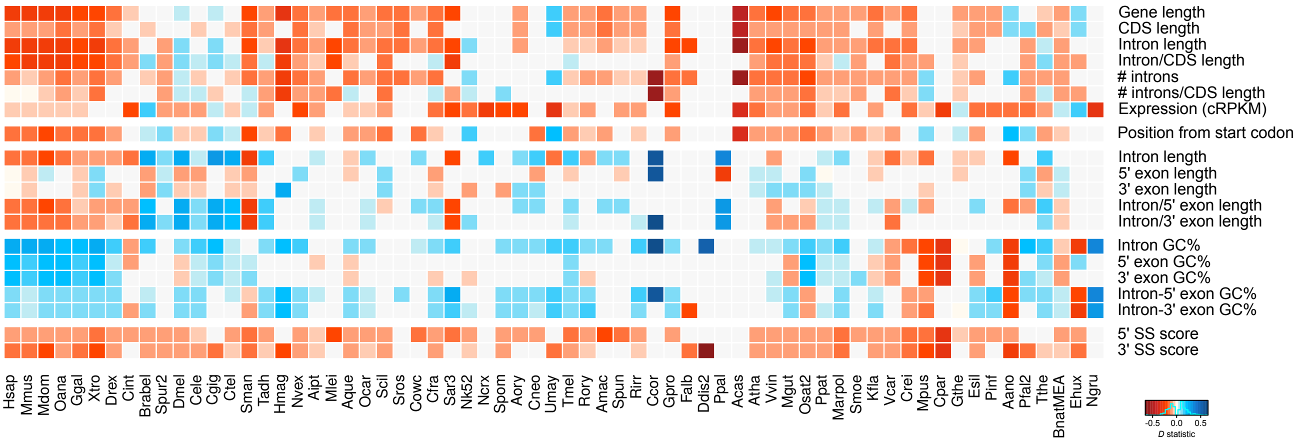

D) Correlation of IR events and gene architecture, transcriptome at 15x sequencing depth

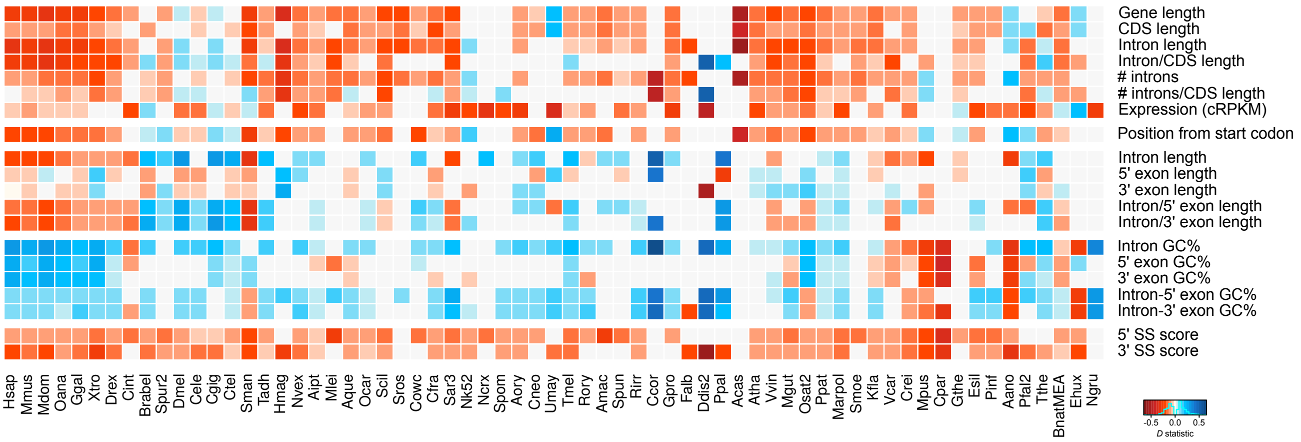

Fig S18. Relationship between gene structure and sequence composition and IR (as in Figure 5), downsampling each RNA-seq sample at 2x (A), 5x (B), 10x (C), and 15x (D).

Each heatmap represents the distance between distributions of gene architecture values for IR-positive and IR-negative introns, measured with the D statistic of the one-sided Kolmogorov-Smirnov two-sample test (significant if $p<0.01$; otherwise grey). D values are recorded as positive/negative (blue/red) according to two one-sided Kolmogorov-Smirnov tests with complementary alternative hypotheses: positive/negative D (blue/red) reflects a positive/negative relationship between IR and the given feature.

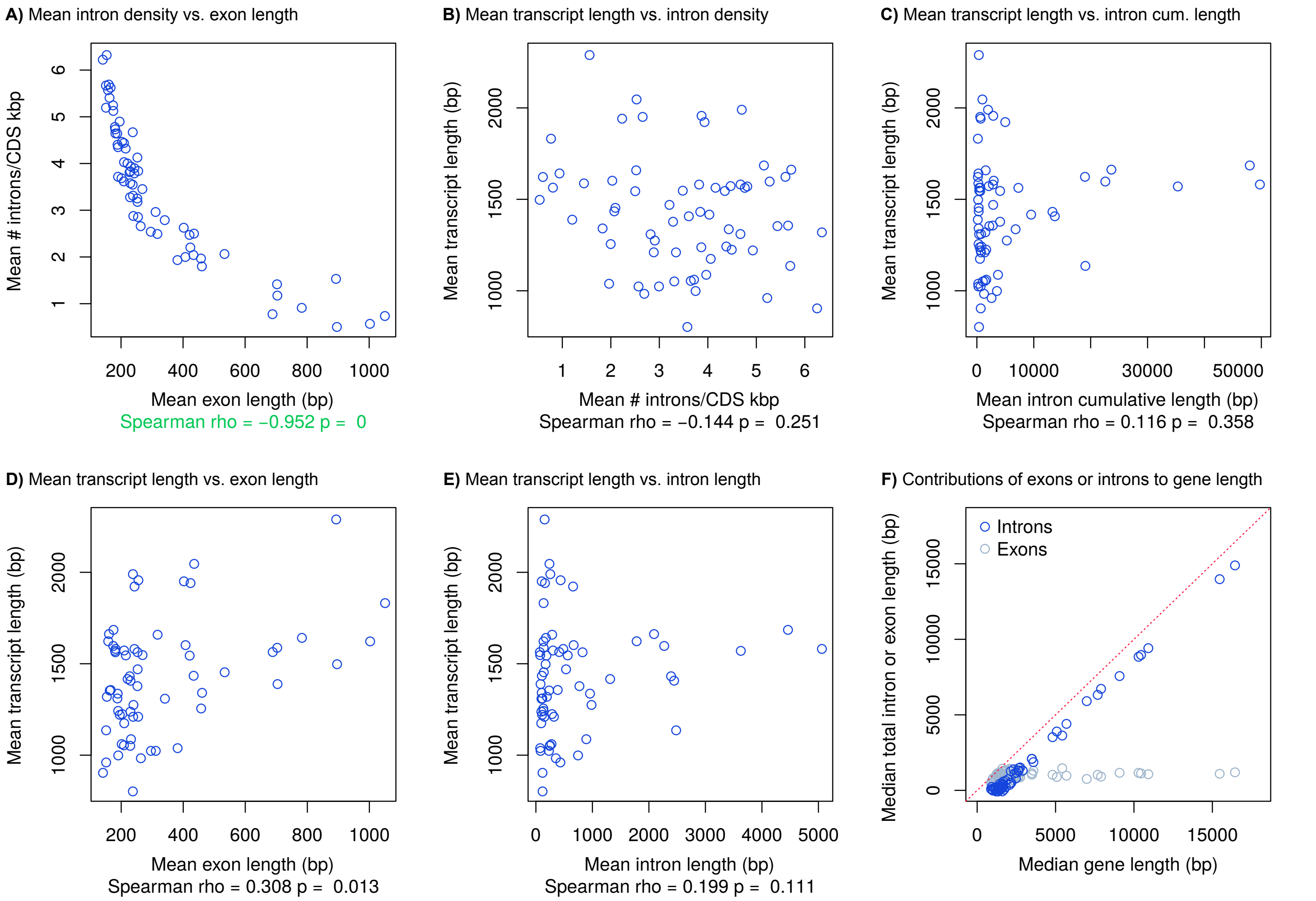

Fig S19. Signatures of intron gain processes in the genome architecture of 65 eukaryotic species.

A) Correlation of mean exon length (bp) and mean intron density (measured as introns/CDS kbp).

B) Correlation of mean intron density (measured as introns/CDS kbp) and mean transcript length (bp).

C) Correlation of mean intron cumulative length (all introns within gene, in bp) and mean transcript length (bp).

D) Correlation of mean exon length (bp) and mean transcript length (bp).

E) Correlation of mean intron length (bp) and mean transcript length (bp).

F) Contribution of cumulative exon and intron lengths to gene length (using median values).

For plots A-E, rho parameter and p-value of Spearman's rank correlation test are indicated. Note that all correlations are non-significant (at the $p<0.01$ level) except for exon length and intron density (panel A). This plot indicates that transcript size remains relatively constant across eukaryotes and does not increase in organisms that have undergone more intron gains (panel B). On the other hand, as new introns are inserted in transcripts of quasi-constant mean length, exons become distinctly shorter (panel A).

species	Architectural feature	Spearman correlation pval	Rho statistic
Hsap	crpkm	5.36E-84	-0.2413
Hsax	crpkm	2.53E-188	-0.1878
Mmus	crpkm	9.28E-32	-0.1138
Mmux	crpkm	2.60E-36	-0.2209
Mdom	crpkm	3.63E-30	-0.1670
Oana	crpkm	4.69E-02	-0.0755
Ggal	crpkm	1.81E-55	-0.2257
Xtro	crpkm	8.07E-01	0.0074
Xtrx	crpkm	2.09E-25	-0.2413
Drex	crpkm	6.13E-01	0.0088
Drer	crpkm	1.83E-08	-0.1736
Cint	crpkm	2.09E-12	-0.3077
Bflo	crpkm	4.36E-03	-0.1309
Spur2	crpkm	4.56E-07	-0.2382
Dmel	crpkm	2.43E-85	-0.2300
Dmel2	crpkm	2.58E-03	-0.0878
Cele	crpkm	0.00E+00	-0.2796
Cgig	crpkm	9.62E-02	0.0432
Ctel	crpkm	1.15E-03	-0.0544
Sman	crpkm	9.18E-22	-0.2332
Tadh	crpkm	2.52E-29	-0.3873
Hmag	crpkm	3.67E-01	-0.0547
Nvec	crpkm	3.24E-131	-0.4115
Nvex	crpkm	7.43E-05	0.0812
Aipt	crpkm	4.56E-67	-0.3963
Mlei	crpkm	8.11E-01	-0.0181
Aque	crpkm	1.11E-86	-0.2950
Ocar	crpkm	5.26E-18	-0.2139
Scil	crpkm	3.44E-37	-0.4087
Sros	crpkm	6.27E-06	-0.3244
Cowc	crpkm	4.51E-02	-0.0562
Cfra	crpkm	2.06E-60	-0.2939
Sar3	crpkm	1.09E-20	-0.3590
Nk52	crpkm	6.20E-21	-0.2852
Ncrx	crpkm	3.99E-06	-0.1911
Spom	crpkm	3.34E-12	-0.2235
Aory	crpkm	3.12E-28	-0.4089
Cneo	crpkm	2.07E-16	-0.3309
Umay	crpkm	1.99E-11	-0.3438
Tmel	crpkm	4.77E-02	-0.0396
Rory	crpkm	7.76E-17	-0.2076
Amac	crpkm	2.39E-05	-0.2320
Spun	crpkm	1.71E-33	-0.4273
Rirr	crpkm	1.44E-41	-0.3523
Rirr2	crpkm	1.92E-05	-0.5855
Ccor	crpkm	9.48E-01	-0.0096
Gpro	crpkm	6.01E-15	-0.2849
Falb	crpkm	1.14E-03	-0.3323
Ddis2	crpkm	1.12E-01	-0.2519
Ppal	crpkm	3.57E-02	-0.1777
Acas	crpkm	8.00E-02	-0.2838
Atha	crpkm	7.51E-20	0.1186
Atha3	crpkm	8.25E-08	-0.1607
Vvin	crpkm	1.80E-52	-0.2406
Mgut	crpkm	4.90E-07	-0.1147
Osat2	crpkm	2.43E-19	-0.3509
Ppat	crpkm	1.38E-134	-0.3727
Smoe	crpkm	4.58E-01	-0.0379
Kfla	crpkm	3.37E-05	-0.0951
Vcar	crpkm	1.60E-01	-0.0159
Crei	crpkm	7.25E-13	-0.1689
Mpus	crpkm	1.25E-04	-0.1552
Cpar	crpkm	5.05E-03	-0.3014
Gthe	crpkm	6.34E-27	-0.1286
GtheME	crpkm	7.74E-55	-0.3847
Esil	crpkm	3.70E-20	-0.1812
Pinf	crpkm	7.40E-17	-0.2557
Aano	crpkm	2.55E-10	-0.2615
Pfal2	crpkm	3.90E-04	-0.0925
Tthe	crpkm	2.41E-57	-0.3247
BnatMEA	crpkm	2.89E-14	**-0.0652**
Bnat	crpkm	8.10E-103	-0.3806
Bnao	crpkm	4.37E-67	-0.2128
Ehux	crpkm	5.14E-01	0.0336
Ngru	crpkm	1.15E-06	-0.3426
Marpol	crpkm	1.96E-19	-0.1559
Brabel	crpkm	1.51E-01	0.0230

Spearman correlation between IR levels in IR-positive introns and crpkm
Negative rho=negative association
Significant if p<0.01

Fig S20. Correlation analysis of IR and expression rates among IR-positive events, with Spearman correlation test.

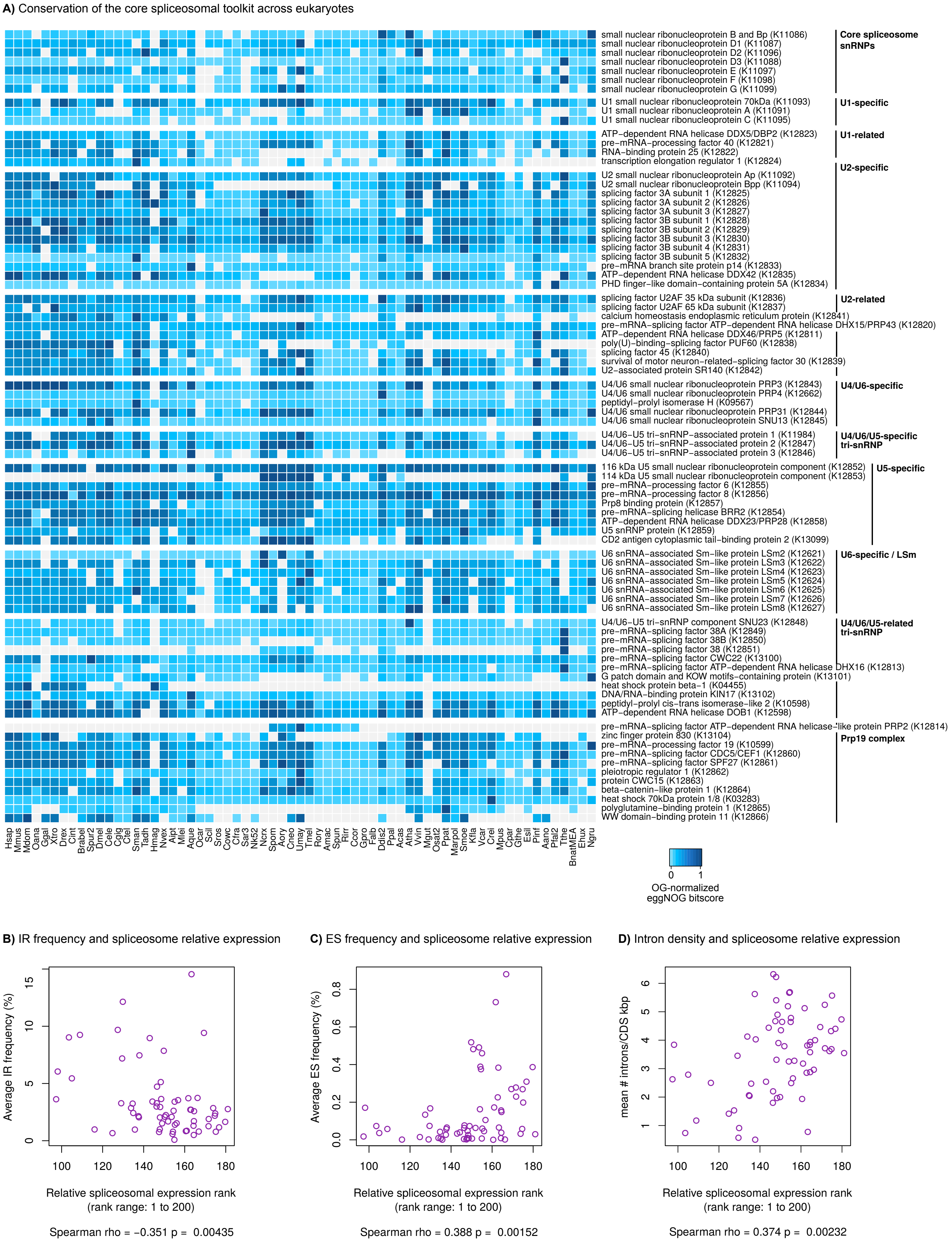

Fig S21. Evolutionary conservation of the core spliceosome.

A) Heatmap representing presence/abscence of core spliceosomal genes across eukaryotes, as identified by eggNOG mapper and KEGG orthologous groups (KEGG codes in brackets). Blue shadings indicate ortholog presence, and a color-scale has been added to indicate the OG- normalized bitscore value as obtained in eggNOG.

B) Correlation between average IR frequencies (as in Supplementary Figure S6) and the relative rank of expression of the spliceosomal genes, per species.

C) Correlation between average ES frequencies (as in Figure 2) and the relative rank of expression of the spliceosomal genes, per species.

D) Correlation between intron content (introns/CDS kbp) and the relative rank of expression of the spliceosomal genes, per species.

See Methods for details on the calculation of relative rank of expression.

Coefficients summary and significance (Wald test)

Variable	Estimate	Std. Error	z statistic (Wald test)	P-value (Wald test)
(Intercept)	6.00E+00	2.63E-01	22.80	4.56E-115
lengene	2.46E-06	6.16E-07	3.99	6.60E-05
lencds	2.81E-05	1.04E-05	2.71	6.79E-03
ralincd	8.14E-03	1.86E-03	4.36	1.28E-05
numin	-8.92E-03	2.35E-03	-3.80	1.43E-04
ranincd	-2.88E-02	8.63E-03	-3.33	8.60E-04
lenex	-6.90E-05	6.55E-05	-1.05	2.92E-01
lenin1	-5.44E-06	8.57E-06	-0.64	5.25E-01
lenin2	-5.18E-05	1.03E-05	-5.02	5.20E-07
ralinex1	6.30E-03	1.00E-03	6.28	3.40E-10
ralinex2	1.41E-02	1.21E-03	11.69	1.51E-31
ss5score	-3.98E-01	1.94E-02	-20.50	2.27E-93
ss3score	-5.91E-01	3.45E-02	-17.12	1.12E-65
is3n	8.86E-01	3.23E-02	27.41	2.36E-165

ANOVA deviance table (variables added sequentially)

Variable	Df	Deviance	Resid. Df	Resid. Dev	Pr(>Chi)
NULL	NA	NA	18635	25834.98	NA
lengene	1	898.05	18634	24936.93	2.60E-197
lencds	1	18.93	18633	24918.00	1.35E-05
lenints	0	0.00	18633	24918.00	NA
ralincd	1	157.51	18632	24760.49	3.97E-36
numin	1	26.33	18631	24734.16	2.87E-07
ranincd	1	7.27	18630	24726.89	7.03E-03
lenex	1	38.66	18629	24688.24	5.05E-10
lenin1	1	54.61	18628	24633.63	1.47E-13
lenin2	1	81.37	18627	24552.26	1.87E-19
ralinex1	1	249.31	18626	24302.95	3.67E-56
ralinex2	1	183.87	18625	24119.08	6.91E-42
ss5score	1	475.83	18624	23643.25	1.73E-105
ss3score	1	334.52	18623	23308.73	9.95E-75
is3n	1	761.47	18622	22547.26	1.29E-167

**NULL deviance**	25834.98171
**Model deviance**	22547.2616

Gene features

lengene	gene length
lencds	CDS length
ralincd	ration intron length/CDS length
ranincd	number of introns/CDS length
lenex	exon length
lenin1	5’ intron length
lenin2	3’ intron length
ralinex1	5’ intron length/exon length
ralinex2	3’ intron length/exon length
ss5score	5’ SS heterogeneity score
ss3score	3’ SS heterogeneity score
is3n	boolean: is exon length 3-divisble?

Species list **Total=24**

Mmus
Mdom
Ggal
Xtro
Drex
Brabel
Dmel
Cele
Cgig
Ctel
Hmag
Nvex
Aipt
Mlei
Aque
Scil
Cfra
Sar3
Atha
Vvin
Mgut
Marpol
Vcar
Crei

Fig S22. Coefficients of the logistic binomial regression model of ES prediction, plus their significance according to Wald test and its corresponding ANOVA deviance table with sequential Chi-square tests.

Variable	Mean	Standard deviation	Distribution type
Gene length	9.29036105	1.09385885	Log-normal
CDS length	7.575259591	0.638614526	Log-normal
Exon length	4.750703651	0.475946827	Log-normal
Intron length	Input-dependent	1.21862551	Log-normal
5’ SS heterogeneity score	9.267095764	0.850434144	Normal
3’ SS heterogeneity score	5.146840410	0.479275742	Normal

Species list	Total=24
Mmus	
Mdom	
Ggal	
Xtro	
Drex	
Brabel	
Dmel	
Cele	
Cgig	
Ctel	
Hmag	
Nvex	
Aipt	
Mlei	
Aque	
Scil	
Cfra	
Sar3	
Atha	
Vvin	
Mgut	
Marpol	
Vcar	
Crei	

Fig S23. Summary of input parameters for the simulation of genome architectures, including the distribution shape, means and standard deviations for each variable (gene, CDS, exon and intron lengths, and 5’ and 3’ SS heterogeneity). Includes the list of species employed in this analysis.

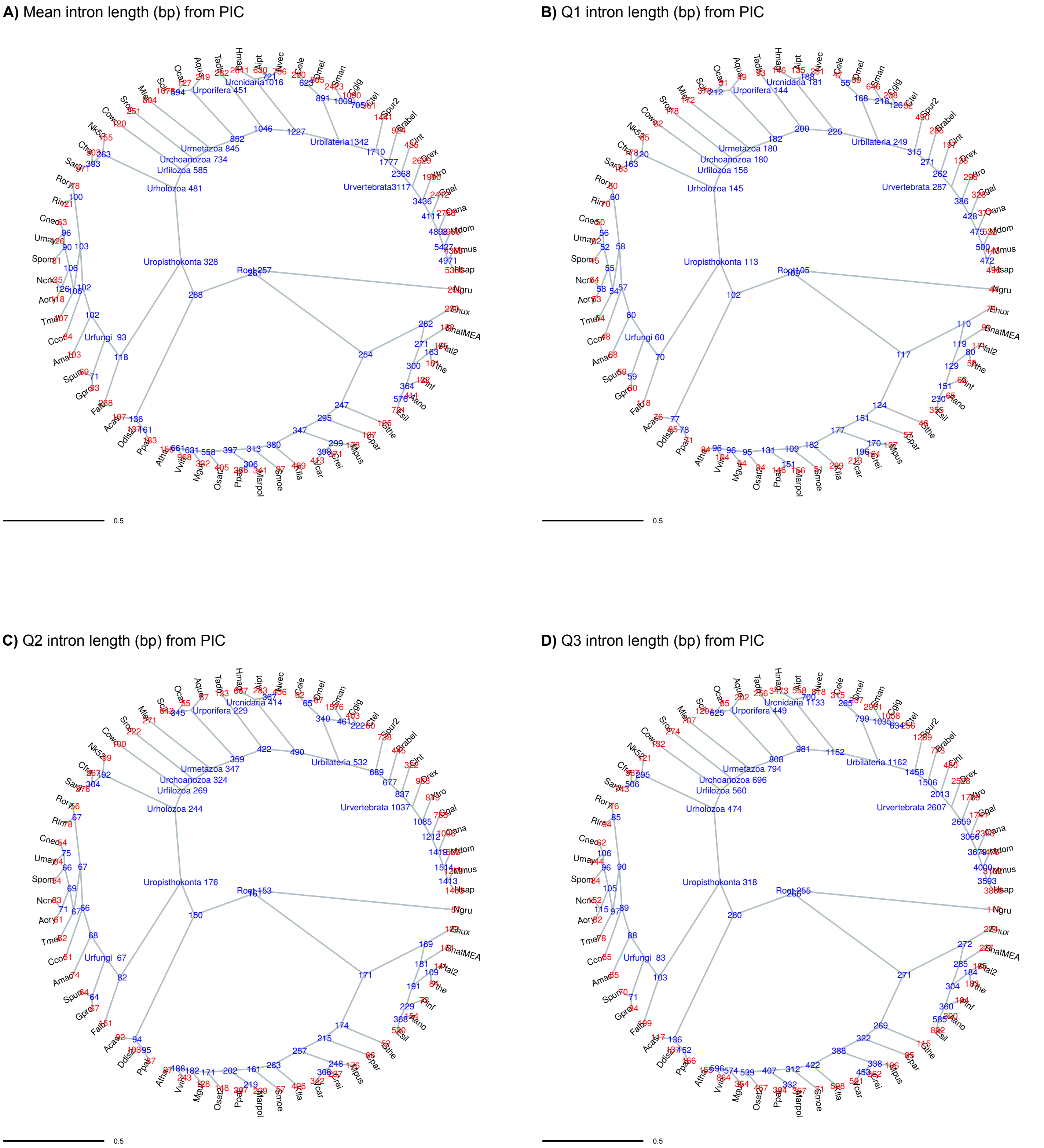

Fig S24. Estimations of ancestral intron length distributions using phylogenetically independent contrasts (PIC) under a 65-species phylogenetic tree obtained from the concatenation of 429 single-copy orthologs, using a constrained topology (see Methods).

Each tree displays the following statistics in ancestors (blue) and extant (red) organisms: A) mean, B) first quartile, C) second quartile and D) third quartile.

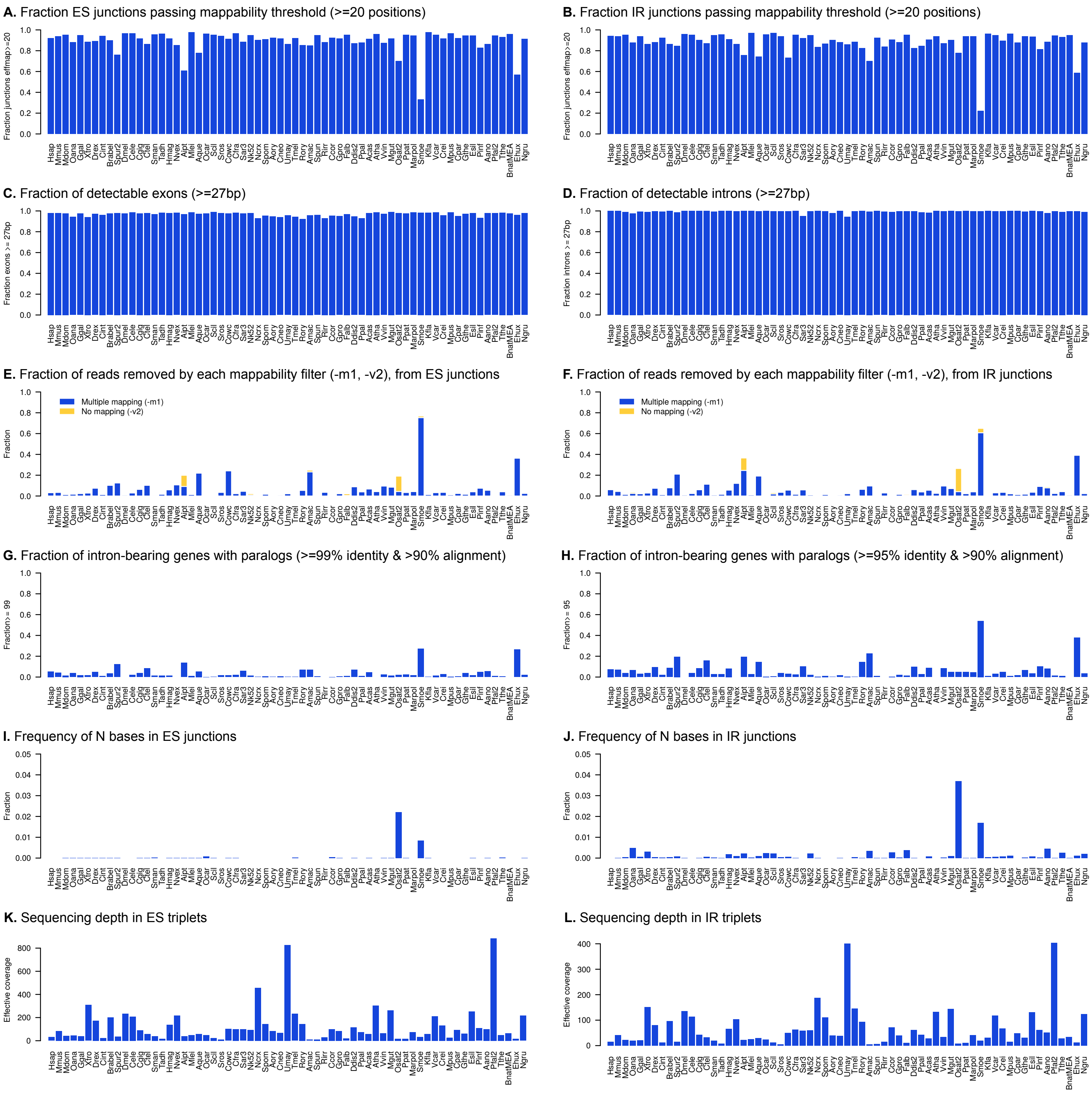

Fig S25. Quality control of the ES and IR detection procedures, analysing possible effects of genome architecture.

A-B) Fraction of exons and introns that pass the mappability filtering step (≥20 mappable positions) in the ES and IR analyses, respectively.

C-D) Fraction of exons and introns with lengths above the minimal detection length imposed by the mappability filter (≥27 bp for ≥20 mappable positions in a 84 bp junction).

E-F) Fraction of reads discarded in the RNA-seq mapping filters due to multiple mapping (-m1) or excess of mismatches (-v2), for ES and IR junctions respectively.

G-H) Fraction of intron-bearing genes with at least one close paralog (which can hinder RNA-seq mapping by multiple mapping problems), as measured using two thresholds: >99% or >95% amino acidic sequence identity, and >90% reciprocal sequence alignment.

I-J) Fraction of uncalled bases (N) in ES and IR junctions of each species, respectively (not scaled from 0-1).

K-L) Sequencing depths for the triplets of ES and IR junctions of each species, respectively (using mappability-corrected number of reads).
